# Pharmacological Effects of NADPH Oxidase Inhibitors on Butterfly Wing Morphogenesis and Color Pattern Formation in *Junonia orithya*

**DOI:** 10.3390/insects17030300

**Published:** 2026-03-10

**Authors:** Yugo Nakazato, Momo Ozaki, Ryunosuke Suenaga, Joji M. Otaki

**Affiliations:** The BCPH Unit of Molecular Physiology, Department of Chemistry, Biology and Marine Science, Faculty of Science, University of the Ryukyus, Nishihara 903-0213, Okinawa, Japan

**Keywords:** apoptosis, butterfly wing, color pattern formation, eyespot, hydrogen peroxide, marginal band system, NADPH oxidase, sexual dimorphism, wing morphogenesis

## Abstract

During the early pupal stage in butterflies, the peripheral portion of wing tissue undergoes apoptosis to finalize adult wing morphology, and wing color patterns are determined coordinately. We hypothesized that the development of wing morphology and color patterns may involve NADPH oxidase (NOX). To test this hypothesis, we treated pupae of the blue pansy butterfly *Junonia orithya* with NOX inhibitors. When VAS2870, isuzinaxib, or diphenyleneiodonium chloride (DPI) in dimethyl sulfoxide (DMSO) was topically applied to the pupal wing tissue via the sandwich method, wing morphology and color pattern elements, including eyespots, parafocal elements, submarginal bands, and marginal bands, were severely deformed as if the marginal area were surgically removed. The topical application of DMSO alone mildly deformed and enlarged eyespots without affecting other color patterns and wing morphology. When systemically injected into pupae, VAS2870 increased eyespots in males but decreased eyespots in females, likely due to the sexual dimorphism of this species. These results suggest that NOX and probably hydrogen peroxide play important roles in wing morphogenesis and color pattern fate determination in butterfly wings. Sexually dimorphic eyespot size in this species may also be explained by the sexually differential activities of NOX.

## Simple Summary

Butterfly wing morphology is determined during the early pupal stage, when the peripheral portion of the pupal wing tissue undergoes apoptosis. We hypothesized that the development of wing morphology and color patterns may involve NADPH oxidase (NOX). To test this hypothesis, we treated pupae of the blue pansy butterfly *Junonia orithya* with NOX inhibitors. When VAS2870, isuzinaxib, or diphenyleneiodonium chloride (DPI) was topically applied to pupal wing tissue, wing morphology and color pattern elements were severely deformed. When systemically injected into pupae, VAS2870 increased eyespot size in males but decreased eyespot size in females, likely due to the sexual dimorphism of this species. These results suggest that NOX and probably hydrogen peroxide play important roles in wing morphogenesis and color pattern fate determination in butterfly wings.

## 1. Introduction

Embryonic development involves dynamic cellular movement and the three-dimensional folding of cellular sheets, as exemplified by the process of gastrulation in amphibian embryos [1,2]. Such dynamic cellular activities are most prevalent in the early stages of life in vertebrates. However, because insect development accompanies metamorphosis, dynamic cellular activities are important in both early and late developmental stages of larval imaginal discs and pupal tissues to realize adult morphology. This is especially true in holometabolic insects, including Lepidoptera (butterflies and moths). The evolutionary inventions for wing elaboration are so important that they are key features for defining insect orders. For example, in Lepidoptera, the surface of the wings is covered with fine scales that may show diverse pigment colors and/or structural colors for the overall color patterns [3,4]. In addition to their color pattern diversity, lepidopteran insects have highly diverse wing morphologies, for example, notable hindwing tails.

In butterflies, wing morphogenesis takes place during the early pupal period [3,4,5,6]. During this period, a small number of mitosis events and subsequent terminal differentiation of wing epidermal (epithelial) cells occur [3,4,5,6]. Notably, the peripheral portion of a pupal wing tissue degenerates to finalize the adult wing morphology [4,5,6,7,8,9]. It is this mechanism that realizes the high diversity of wing morphology in Lepidoptera. This process involves not only the development of bordering lacuna along the prospective wing margin but also degeneration via the apoptosis of peripheral epidermal cells [4,5,6,7,8,9]. In real-time in vivo observations, pupal wing tissues exhibit dynamic physical movement [10,11,12], suggesting that the process of peripheral apoptosis may be more complex in association with wing movement. Furthermore, mitotic density is likely important in the development of wing shape [13]. A few genes are expressed in wing imaginal discs in an area-specific manner [9,14], but their precise functions in association with peripheral degeneration and wing morphogenesis are not clear. Similar to the peripheral wing degeneration in butterflies, the sex-dependent wing development of winter moths, in which entire female wings degenerate, involves the apoptosis of wing tissues [15]. This degeneration is induced by ecdysteroid [16,17].

In addition to wing morphology, wing color pattern fates are also determined during the early pupal period, although this determination process begins in the late larval stage. The color patterns are strongly influenced by the overall wing shape. The overall color pattern in butterflies is composed of four symmetry systems (the discal symmetry system, the central symmetry system, the basal symmetry system, and the border symmetry system) and two half-symmetry peripheral systems (the marginal band system and the wing root band system) [3,18,19,20,21]. These systems likely have their own organizers that determine the system’s color patterns. Each of these systems is composed of color pattern elements (such as eyespots in the border symmetry system). Color pattern elements in the same system and in different but adjacent systems seem to interact with one another to finalize the elemental positions during development [22,23,24]. The marginal band system and the border symmetry systems appear to repel each other so that their color patterns do not overlap [25]. When the marginal area of the prospective adult wing is physically removed, the marginal band system cannot develop, likely because its organizers are removed, and an eyespot is diffused and flattened along the artificial wing margin [4,26]. When the marginal organizer is damaged, the marginal band (MB) and submarginal band (SMB) are disturbed, and the parafocal element (PFE) is positioned more distally, likely because of the lack of repulsion from the marginal band system [25]. Furthermore, wing veins define wing compartments (wing cells), and this venation system is required to properly develop color pattern elements on the wings [4,27]. The wing veins likely cut the color pattern signals from the previous organizer into pieces that fit in each wing compartment [22].

Although the development of wing morphology can be considered an important model system for investigating animal morphogenesis [9,14], the molecular mechanisms of peripheral apoptosis in butterfly pupal wing tissues are largely unknown. In general, one of the apoptosis-regulating enzymes is the family of NADPH oxidases (NOX), which regulate reactive oxygen species (ROS), including hydrogen peroxide [28,29,30,31,32,33,34]. Through the production of ROS, NOX proteins have multiple roles, including innate immunity responses and crosslinking of the extracellular matrix, in various organisms including insects [35,36,37,38]. Extracellular ROS is likely a key factor for apoptosis-induced compensatory proliferation after stress-induced cell death [39]. Importantly, in insects, dual NADPH oxidase (DUOX) contributes to the stabilization of wing cuticle [40] and tracheal network [41], likely through tyrosine crosslinking via ROS. Moreover, DUOX appears to be important in various aspects of the insect life cycle, including molting, hatching, and feeding [42]. Various types of ROS are produced in many biological systems, of which hydrogen peroxide has been recognized as a signaling molecule [43,44,45,46,47,48,49,50,51,52]. Importantly, hydrogen peroxide is a cell-surviving signaling molecule at low concentrations, although it is generally harmful at high concentrations [46,53]. We hypothesized that a type of NOX is involved in the regulation of hydrogen peroxide to define the apoptotic and nonapoptotic regions of the pupal peripheral wings in butterflies. We also hypothesized that NOX-derived hydrogen peroxide is involved in color pattern determination in butterfly wings. To examine these possibilities, we employed VAS2870, a specific inhibitor of NOX without isoform specificity [54,55,56,57,58]. VAS2870 has been used in a variety of biological systems to demonstrate the physiological function of NOX [59,60,61,62,63]. In addition, we tested two additional NOX inhibitors, isuzinaxib and diphenyleneiodonium chloride (DPI). Both isuzinaxib [64,65,66,67] and DPI [68,69,70] have been used as widely as VAS2870.

In this study, we used the blue pansy butterfly *Junonia orithya* as an experimental system of choice. We topically applied NOX inhibitors on the surface of the hindwings of fresh pupae. We then tested whether the effects of NOX inhibitors can be rescued via the injection of hydrogen peroxide. Additionally, we systemically injected VAS2870 into fresh pupae. We then examined the wing morphology and color patterns of adult butterflies after eclosion. Because VAS2870, isuzinaxib, and DPI were dissolved in dimethyl sulfoxide (DMSO), we also examined the effects of DMSO on wing morphology and color patterns for comparison.

## 2. Materials and Methods

### 2.1. Butterflies

The blue pansy butterfly *J. orithya* (Linnaeus, 1758) was used throughout this study. This butterfly is abundant on Okinawa-jima Island, Okinawa Prefecture, Japan, where there is no restriction on its collection and use for research. Female butterflies were collected on the Nishihara Campus of the University of the Ryukyus, Okinawa, Japan. The collected female butterflies were confined in a glass tank (300 mm × 300 mm × 300 mm) together with the natural host plant *Phyla nodiflora* for oviposition. Larvae were reared with the natural host plant *Plantago asiatica* in a plastic container at ambient temperature (approximately 27 °C) under L16:D8 light conditions.

### 2.2. Experimental Treatments

Immediately after pupation (within 30 min postpupation), the left forewing was physically lifted, a liquid droplet (0.6 μL) was placed on the surface of the hindwing, and the forewing was carefully placed back to the original position. In this way, a liquid droplet was sandwiched between the forewing and the hindwing (henceforth called the sandwich method). The right wing was not treated and was used for comparison. For systemic applications, 2.0 μL of liquid was injected into the abdomen of a fresh pupa within 1 h postpupation via an Ito microsyringe MS-05 (Fuji, Shizuoka, Japan). The treated pupae were individually placed in a container until eclosion. Adult butterflies were frozen immediately after eclosion before wing damage could occur. The survival rates for these treatments are recorded in Appendix A Table A1.

We also performed a rescue experiment in which VAS2870 (13.87 mM) was applied topically via the sandwich method, and simultaneously, either water or 12% hydrogen peroxide solution (obtained as 30% solution; FUJIFILM Wako Pure Chemical, Osaka, Japan) (2.0 μL) was injected. This experiment was based on the hypothesis that the hydrogen peroxide produced from endogenous NOX during wing morphogenesis is inhibited by VAS2870, and its effects could be rescued by the exogeneous supply of hydrogen peroxide, although the biological half-lives of VAS2870 and hydrogen peroxide in pupae may be different.

### 2.3. Chemical Compounds

The NOX inhibitor VAS2870 (Cayman Chemical, Ann Arbor, MI, USA) was used in this study. This compound contains a sulfur atom, and its formal name is 7-(2-benzoxazolylthio)-3-(phenylmethyl)-3H-1,1,3-triazolo[4,5-d]pyrimidine, according to the manufacturer’s product information. Two additional NOX inhibitors, isuzinaxib (also known as APX-115 free base or Ewha-18278 free base) (Selleckchem, Houston, TX, USA) and diphenyleneiodonium chloride (DPI) (MedChemExpress, Monmouth Junction, NJ, USA) were similarly used. These inhibitors were dissolved in dimethyl sulfoxide (DMSO) (Wako Pure Chemical Industries, Osaka, Japan), and several concentrations were applied as shown in Appendix A Table A1. Owing to its high degree of cellular penetrance and solubilizing ability, DMSO may disrupt wing morphogenesis and color pattern formation. Thus, we evaluated the effects of DMSO alone and NOX inhibitors in DMSO simultaneously.

### 2.4. Experimental Evaluations

We mainly focused on dorsal hindwing eyespots because they are accessible via the sandwich method. For the quantification of eyespot areas, only the posterior eyespot was examined in males because anterior eyespots are often nonexistent or undeveloped in males. In females, both the anterior and posterior eyespots were examined.

**Figure 1:**
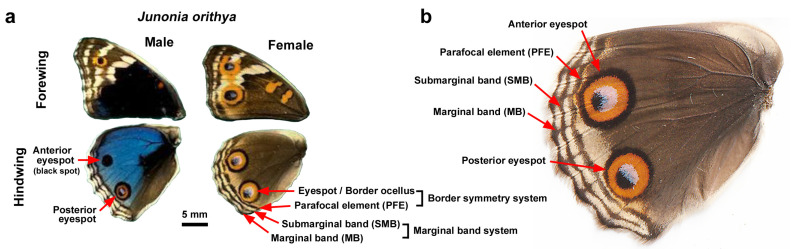
Nomenclature of color pattern elements on the dorsal hindwings in *J. orithya*. (**a**) Male and female dorsal wings without experimental treatment. This species is sexually dimorphic. A dorsal hindwing has anterior and posterior eyespots, but the male anterior eyespot is mostly black and is often a small dot or is not expressed at all. (**b**) Female dorsal hindwing without experimental treatment.

We obtained images of the dorsal hindwings of the adult butterflies that eclosed in our laboratory via a Keyence Digital Microscope VHX-7000 (Osaka, Japan). For qualitative analysis, the color pattern elements that were changed in location, shape, and size were noted in reference to the nomenclature shown in Figure 1 in accordance with the nymphalid ground plan [4,20]. Wing shape changes were also noted. For quantitative analysis, an eyespot area was defined and measured via the brightness function of the microscope. To standardize the eyespot area values in accordance with wing size, the distance between the two wing veins where a given eyespot was present was measured using ImageJ 1.54g (National Institute of Health, Bethesda, MD, USA). To obtain the standardized eyespot size, the eyespot area value was divided by the distance squared.

Because DMSO solvent alone was found to increase eyespot size (Section 3.3), the effect of VAS2870 was corrected in accordance with the effect of DMSO alone when the DMSO-injected control group was not available in the same sibling group. To do so, the VAS2870-injected eyespot size was divided by the DMSO-induced fold change value of eyespot size.

Statistical evaluation (unpaired bisided Student *t*-test or Welch *t*-test after *F*-test) of eyespot size was performed using Microsoft Excel (Microsoft Office 365) and JSTAT 16.1 (Yokohama, Japan). Fisher’s exact test was performed to evaluate the results of the rescue experiments using JSTAT 16.1.

## 3. Results

### 3.1. Topical Application of DMSO

**Figure 2:**
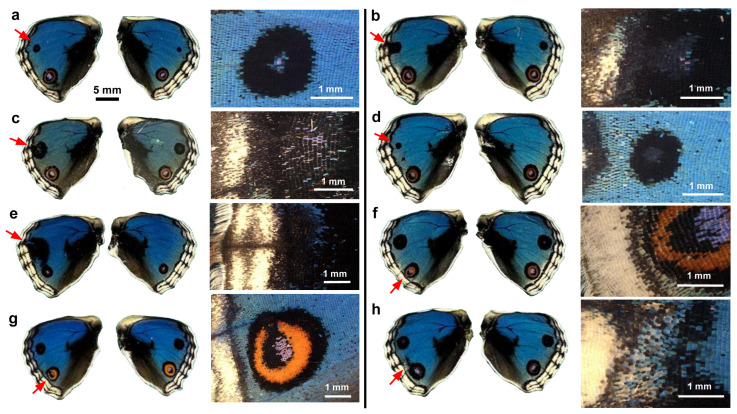
Topical application of DMSO to males via the sandwich method. Only the left wing was treated, and the right wing was not treated and saved as a control. Eight treated individuals (**a**–**h**) with successful eclosion are shown. The scale bar in (**a**) is applicable to all whole-wing images in this figure. A portion of a treated wing (red arrow) is enlarged in the right panel for each individual.

Because NOX inhibitors were dissolved in DMSO, we first evaluated the possible effects of DMSO alone on wing morphogenesis and color pattern formation. To directly deliver DMSO to the wing tissue, we topically applied it on the surface of the left dorsal hindwing using the sandwich method. Qualitative visual inspection revealed that, in comparison with the right wing, the anterior eyespot (a black spot in most individuals) tended to be enlarged in males (*n* = 8) (Figure 2a–h). One individual presented a large black area covering the anterior eyespot (Figure 2e). The posterior eyespot tended to deform without extensive size changes (Figure 2f–h). The MB, SMB, and PFE were not severely affected. Overall, color pattern changes in response to DMSO were not extensive in males.

**Figure 3:**
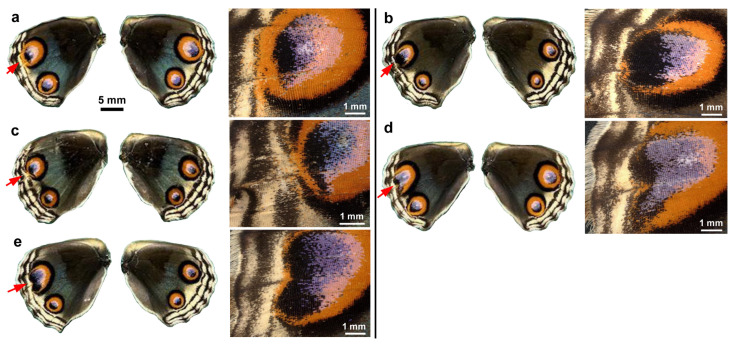
Topical application of DMSO to females via the sandwich method. Only the left wing was treated. The right wing was not treated and was saved as a control. Five treated individuals (**a**–**e**) with successful eclosion are shown. The scale bar in (**a**) is applicable to all whole-wing images in this figure. A portion of a treated wing (red arrow) is enlarged in the right panel for each individual.

We then observed females that were subjected to the same treatment. Qualitative visual inspection revealed that the anterior eyespot tended to be enlarged and open at the distal side so that the eyespot was in contact with the PFE (*n* = 5) (Figure 3a–e). In one individual, the position and shape of the PFE touching an eyespot were disrupted (Figure 3c). Only mild deformation of the wing shape was observed. These results revealed the ability of DMSO to modify color pattern elements, especially eyespots, both in males and females.

### 3.2. Topical Application of VAS2870 in DMSO

**Figure 4:**
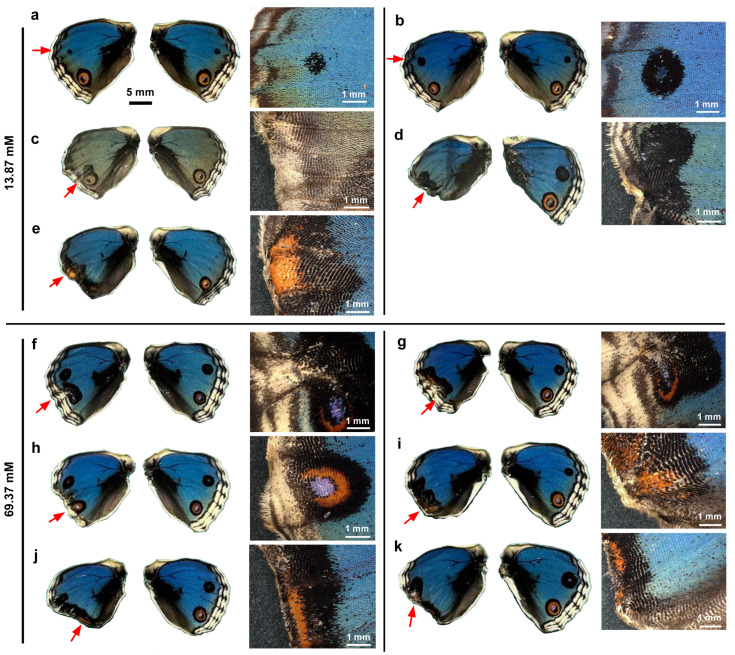
Topical application of VAS2870 in DMSO to males via the sandwich method. Only the left wing was treated, with the right wing saved as a control. Five treated individuals (13.87 mM) (**a**–**e**) and six treated individuals (69.37 mM) (**f**–**k**) with successful eclosion are shown. The scale bar in (**a**) is applicable to all whole-wing images in this figure. A portion of a treated wing (red arrow) is enlarged in the right panel for each individual.

We dissolved VA2870 in DMSO and applied it topically to fresh pupae immediately after pupation via the sandwich method. Qualitatively, at a relatively low concentration (13.87 mM) in males (*n* = 5), no color pattern changes were observed in one male individual (Figure 4a), but in another male individual, the anterior eyespot (black spot) appeared to be enhanced (Figure 4b). However, at the same concentration, in other individuals, the peripheral wing area was lacking, like when ablated, resulting in an abnormal wing shape (Figure 4c–e). In two individuals, eyespots were completely compromised; eyespots were spread along the wing margin where the MB, SMB, and PFE were no longer present (Figure 4d,e). Despite the lack of these elements, long marginal scales were present in at least one individual (Figure 4e).

Essentially similar results were observed at a higher concentration (69.37 mM) in males (*n* = 6) (Figure 4f–k). In two individuals, eyespots were severely disrupted, but the MB, SBM, and PFE were still identifiable, suggesting that VAS2870 acted directly on eyespots more than on the peripheral area in these cases (Figure 4f,g). In the other four individuals, the peripheral wing proper was lacking, and the elements were no longer identifiable, but the orange area along the wing margin suggested that eyespots were compromised to spread out along the wing margin (Figure 4h–k).

**Figure 5:**
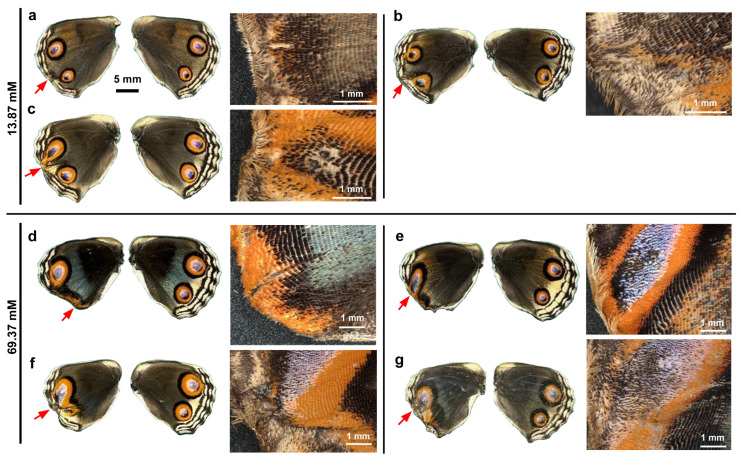
Topical application of VAS2870 in DMSO to females via the sandwich method. Only the left wing was treated, and the right wing was not treated and was saved as a control. Three treated individuals (13.87 mM) (**a**–**c**) and four treated individuals (69.37 mM) (**d**–**g**) with successful eclosion are shown. The scale bar in (**a**) is applicable to all whole-wing images in this figure. A portion of a treated wing (red arrow) is enlarged in the right panel for each individual.

We then visually examined the females. At a relatively low concentration (13.87 mM) in females (*n* = 3), the MB, SMB, and PFE appeared to be inhibited from developing in association with the lack of peripheral wing area (Figure 5a–c). Eyespots appeared to be deformed in response to the deformation of the wing shape and the lack of MB, SMB, and PFE (Figure 5b,c). At a higher concentration (69.37 mM) in females (*n* = 4), the peripheral wing area was lacking and appeared ablated, and eyespots were spread out along the wing margin (Figure 5d) or were completely deformed (Figure 5e–g).

In summary, both males and females were severely affected in a similar fashion; a lack of peripheral wing area (including the elimination of the MB, SMB, and PFE) and the deformation of eyespots were notable.

### 3.3. Systemic Application of DMSO

To further understand the effects of DMSO and VAS2870 on wing morphogenesis and color pattern formation, we systemically applied DMSO alone via the injection method. No clear color pattern changes were observed in any of the individuals treated (*n* = 15 in males and *n* = 15 in females) compared with the no treatment sibling group (*n* = 13 in males and *n* = 18 in females) (Supplementary Figure S1).

**Figure 6:**
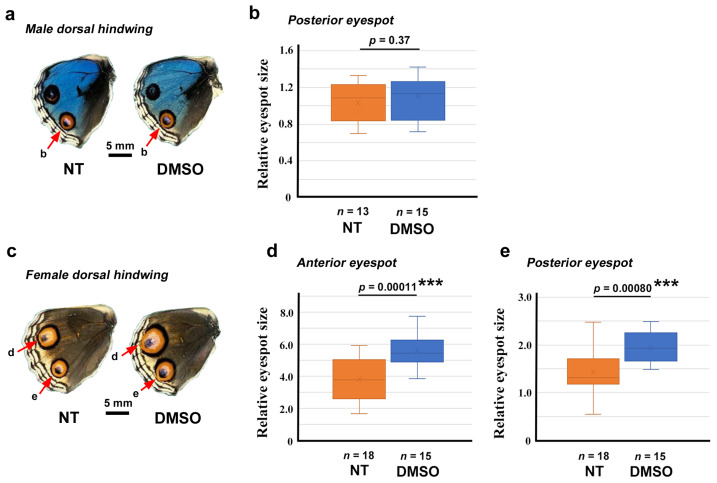
Quantitative evaluation of the effect of DMSO on eyespot size. ***: *p* < 0.001. (**a**) Examples of a control (no treatment, NT) dorsal hindwing (left) and a DMSO-treated dorsal hindwing (right) of males. The posterior eyespots (red arrows) were measured and statistically compared in (**b**). (**b**) Comparison of the relative eyespot size between the NT and DMSO-treated groups of male posterior dorsal hindwings (Student’s *t*-test). (**c**) Examples of a control (NT) dorsal hindwing (left) and a DMSO-treated dorsal hindwing (right) of females. Both anterior eyespots and posterior eyespots (red arrows) were measured and statistically compared in (**d**,**e**). (**d**) Comparison of the relative eyespot size between the NT and DMSO-treated groups of female anterior dorsal hindwings (Student *t*-test). (**e**) Comparison of the relative eyespot size between the NT and DMSO-treated groups of female posterior dorsal hindwings (Student *t*-test).

We then attempted to quantitatively evaluate the possible effects of DMSO on eyespot size. In the male posterior eyespot, no statistically significant difference in relative size was observed between the DMSO-treated group (*n* = 15) and the no treatment group (*n* = 13) (*p* = 0.37) (Figure 6a,b). However, in the female anterior and posterior eyespots, DMSO treatment (*n* = 15) significantly increased the relative eyespot size (*p* = 0.00011 for the anterior eyespot; *p* = 0.00080 for the posterior eyespot) compared with those of the no treatment group (*n* = 18) (Figure 6c–e). For the anterior and posterior eyespots, a 1.48-fold increase and a 1.36-fold increase in relative eyespot size, respectively, were observed.

In summary, DMSO effect was sexually dimorphic. DMSO increased eyespot size in females but not in males. This effect of DMSO via systemic injection appeared to be specific to eyespots, although we did not quantitatively evaluate whether other elements were affected.

### 3.4. Systemic Application of VAS2870 in DMSO

**Figure 7:**
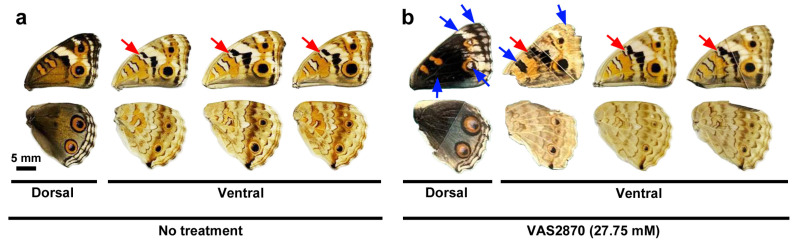
Qualitative evaluation of the effects of VAS2870 (27.75 mM) in DMSO on color patterns in females. (**a**) Examples of the control group (no treatment). The gap between the black band and the black line is indicated by red arrows. The scale bar here is applicable to all wings shown in this figure. (**b**) Modified color patterns on the dorsal and ventral sides. The fusion of the black band and the black line is indicated by a red arrow. Other modifications are indicated by blue arrows. Two wings on the right also show partial fusion of the black band and the black line indicated by red arrows.

To investigate the effects of VAS2870 on wing morphogenesis and color pattern development, we performed systemic application via the injection method. Qualitatively, we did not observe any changes in wing shape or color patterns in three different concentrations at 0.69 mM (*n* = 13 in males and *n* = 18 in females), 6.94 mM (*n* = 15 in males and *n* = 15 in females), and 13.87 mM (*n* = 18 in males and *n* = 13 in females) compared with the nontreated group (*n* = 29 in males and (*n* = 30 in females) in the first sibling group (Supplementary Figure S2). However, in the second sibling group, in comparison with nontreated group (*n* = 24 in males and *n* = 25 in females), one female individual presented relatively extensive color pattern modifications in the forewing on both the dorsal and ventral sides at 27.75 mM (*n* = 9 in males and *n* = 7 in females) (Figure 7a,b; Supplementary Figure S2). In this individual, the black band on the ventral side expanded to fuse with the adjacent black line (Figure 7b). Less extensive but similar fusion of the black band and the black line was observed in two additional female individuals (*n* = 3 in total among seven females) at this concentration (Figure 7b). In the no-treatment group, only one such individual among 25 females was observed, suggesting that the fusion of the black band and the black line was induced by the VAS2870 injection in females. We did not observe similar modifications at 69.37 mM (*n* = 1 for both males and females) compared with those of the nontreated group (*n* = 24 in males and *n* = 25 in females). This is probably because of the small number of successful eclosion events at 69.37 mM.

**Figure 8:**
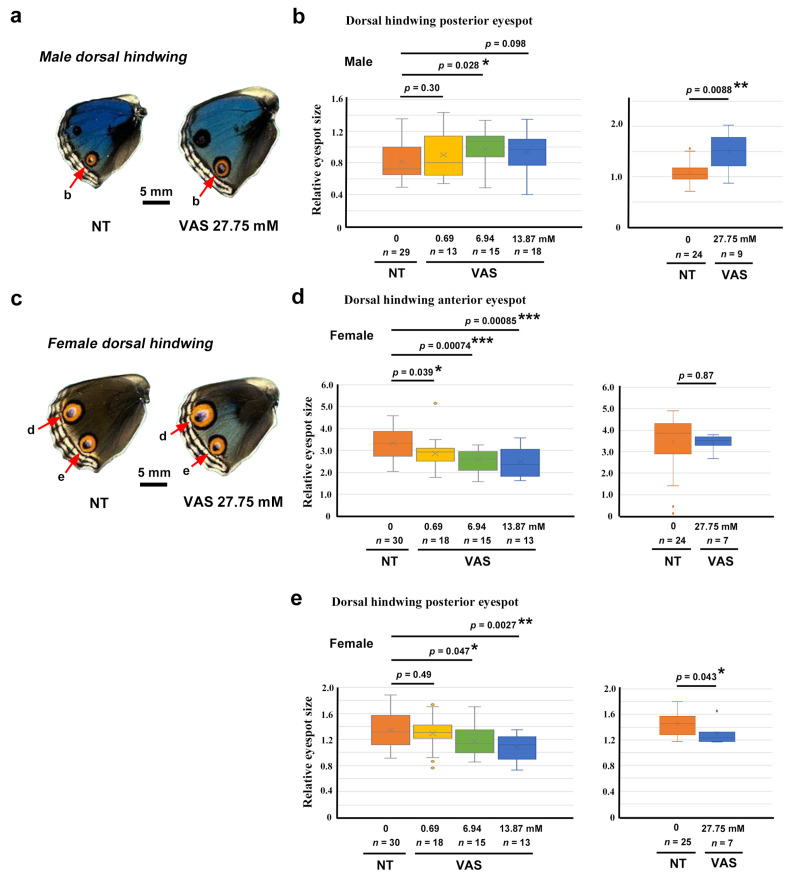
Quantitative evaluation of the effect of VAS2870 in DMSO on eyespot size. *: *p* < 0.05, **: *p* < 0.01, ***: *p* < 0.001. (**a**) Examples of a control (no treatment, NT) dorsal hindwing (left) and a VAS2870-treated dorsal hindwing (right) of males. The posterior eyespots (red arrows) were measured and statistically analyzed in (**b**). (**b**) Comparison of the relative eyespot size between the NT and VAS2870-treated groups of male posterior dorsal hindwings. (Student *t*-test). Each graph indicates each sibling group, as in (**d**,**e**). (**c**) Examples of a control NT dorsal hindwing (left) and a VAS2870-treated dorsal hindwing (right) of females. Both the anterior eyespots and the posterior eyespots (red arrows) were measured and statistically compared in (**d**,**e**). (**d**) Comparison of the relative eyespot size between the NT and VAS2870-treated groups of female anterior dorsal hindwings (Student *t*-test for the left panel and Welch *t*-test for the right panel). (**e**) Comparison of the relative eyespot size between the NT and VAS2870-treated groups of female posterior dorsal hindwings (Student *t*-test).

We then measured the relative area of the eyespots for quantitative evaluation. Compared with the no treatment group (*n* = 29), the VAS2870-injected group had significantly larger relative eyespot sizes at concentrations of 6.94 mM (*n* = 15; *p* = 0.028) and 27.75 mM (*n* = 9; *p* = 0.0088) in males (Figure 8a,b). In females, the relative eyespot area values were corrected in accordance with the DMSO correction factors (Section 2.4 and Section 3.3). In the female anterior eyespot, the VAS2870-injected group had significantly smaller relative eyespot sizes at concentrations of 0.69 mM (*n* = 18; *p* = 0.039), 6.94 mM (*n* = 15; *p* = 0.00074), and 13.87 mM (*n* = 13; *p* = 0.00085) than the no treatment group (*n* = 30 for 0.69 mM, 6.94 mM, and 13.87 mM) (Figure 8c,d). This effect was not observed at 27.75 mM (*n* = 7; *p* = 0.87), compared with the no treatment group (*n* = 24), which may be attributable to the high variation in the control group in this sibling group (Figure 8d). Similarly, in the female posterior eyespot, the VAS2870-injected group had significantly smaller relative eyespot sizes at concentrations of 6.94 mM (*n* = 15; *p* = 0.047), 13.87 mM (*n* = 13; *p* = 0.0027), and 27.75 mM (*n* = 7; *p* = 0.043) than the no treatment group (*n* = 30 for 0.69 mM, 6.94 mM, and 13.87 mM; *n* = 25 for 27.75 mM) (Figure 8e).

In summary, the effect of VAS2870 on eyespot size was sexually dimorphic. Eyespot size increased in males and decreased in females. This effect of VAS2870 via systemic injection appeared to be selective to eyespots to some extent. However, other elements such as the black band in the ventral forewings appeared to be affected in females.

### 3.5. Topical Application of Isuzinaxib in DMSO

**Figure 9:**
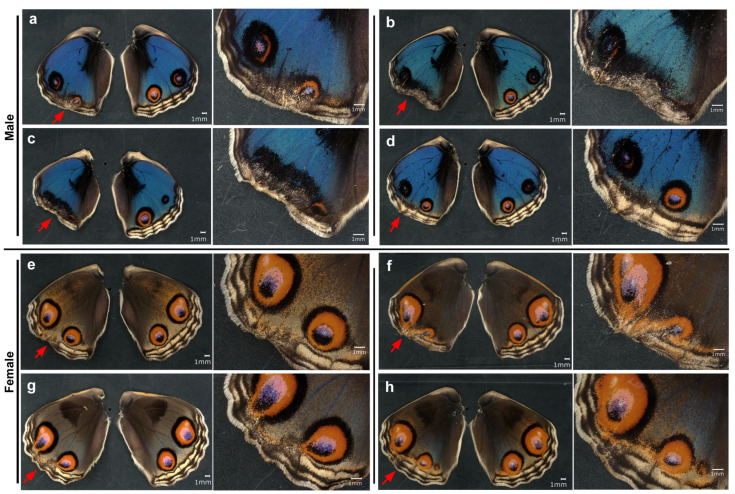
Effects of isuzinaxib in DMSO on wing shape and color patterns. Representative individuals are shown. Wings of all treated individuals are shown in Supplementary Figure S4. Left panels indicate both right and left (treated) wings, and right panels indicate enlarged images of affected wing areas. Red arrows indicate the affected wing margins. (**a**–**d**) Males. (**e**–**h**) Females.

To confirm the effects of VAS2870 above, here we tested another NOX inhibitor, isuzinaxib, via topical application. As in the case of VAS2870, we observed wing shape disruption and color pattern changes (Figure 9; Supplementary Figure S4). Among the treated male individuals (*n* = 17) (Figure 9a–d), eyespots were severely disrupted in 11 individuals. Among them, the peripheral wing proper was severely deleted in two individuals (Figure 9b,c). In these cases, the elements were not clearly identifiable. In one male individual, the wing shape was almost intact, but MB and SMB did not develop well in one compartment (Figure 9d), suggesting the elimination of the organizer for the marginal band system. The treated female individuals (*n* = 10) exhibited similar results (Figure 9e–h), although the deletion level of the wing margin was minor in females compared to males. Eyespots, PFE, SMB, and MB appeared to be deformed in response to the deformation of the wing shape.

In summary, the results of isuzinaxib were phenotypically similar to those of VAS2870 in both males and females. We observed a lack of peripheral wing area and the deformation of certain elements, notably eyespots. These results support the results of VAS2870 and suggest the function of NOX in butterfly wing development.

### 3.6. Topical Application of DPI in DMSO

**Figure 10:**
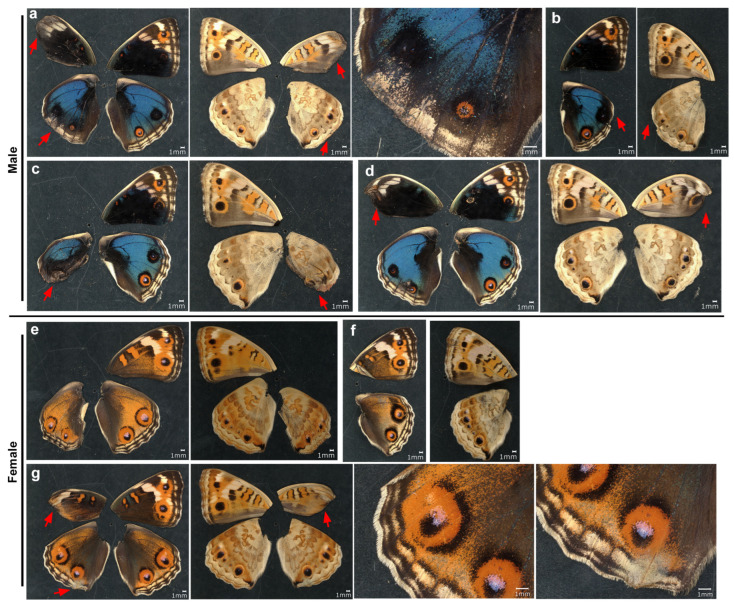
Effects of DPI in DMSO at the highest concentration tested (41.32 mM) on wing shape and color patterns. Left wings were treated, but both right and left wings and both dorsal and ventral sides are shown. Some wings are not shown because they did not eclose. Red arrows indicate disrupted wing margin. (**a**–**d**) Wings from male individuals (*n* = 4). (**e**–**g**) Wings from female individuals (*n* = 3).

To further confirm the effects of VAS2870 and isuzinaxib, we applied three different concentrations of DPI topically via the sandwich method. At the highest concentration tested (41.32 mM), we obtained only seven individuals with successful eclosion; the survival rate was just 19% (Appendix A Table A1). In these individuals, we observed wing shape disruption and color pattern changes as in the case of VAS2870 and isuzinaxib (Figure 10). In two males and one female, the forewing shape was disrupted more than the hindwing shape (Figure 10a,d,g). Despite only the left wings being treated topically, the wing shape of the right hindwing was affected in one individual (Figure 10b), suggesting that DPI reached the right hindwing via hemolymph. Both in right and left dorsal wings in both males and females, the overall color patterns were blurred. The enhancement of black scales was observed in the dorsal wings of males (Figure 10a–d), whereas the enhancement of orange scales was notable in the dorsal wings of females (Figure 10e–g). Lower concentrations of DPI (4.13 mM and 0.41 mM) did not produce the forewing disruption but produced wing shape disruption and color pattern changes in hindwings as in the case of VAS2870 and isuzinaxib (Supplementary Figure S5).

### 3.7. Rescue Experiement via the Injection of Hydrogen Peroxide

**Figure 11:**
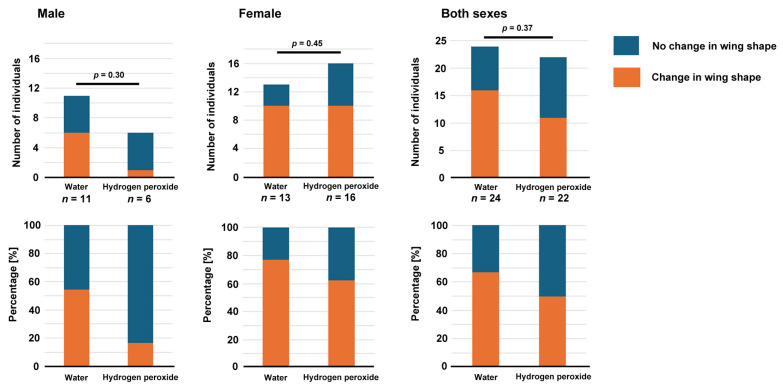
Results of the rescue experiment. The number of individuals with wing shape changes were counted and compared between the two groups (top panels). The results are also shown as percentages (bottom panels).

The results thus far suggest that the NOX inhibitors used in this study inhibited endogenous NOX in wing tissues to produce hydrogen peroxide, which may be necessary for wing morphogenesis and color pattern formation. We tested the hypothesis that the lack of hydrogen peroxide due to the treatment with NOX inhibitors might be compensated for via the exogenous supplementation of hydrogen peroxide via injection. These double treatments (VAS2870 application via the sandwich method and water or hydrogen peroxide application via the injection method) were well tolerated with high survival rates (Appendix A Table A1). In both sexes, the differences between the two treatments (hydrogen peroxide versus water) after the VAS2870 application were not statistically significant, but the percentages of individuals with wing shape changes were lower when hydrogen peroxide was injected than when water was injected (Figure 11).

## 4. Discussion

In this study, we tested the pharmacological effects of three NOX inhibitors (namely, VAS2870, isuzinaxib, and DPI) and DMSO on the development of butterfly wings. Their effects on wing morphology and eyespot size were observed. Because VAS2870, isuzinaxib, and DPI are well-known specific inhibitors of the NOX family proteins [54,55,56,57,58,59,60,61,62,63,64,65,66,67,68,69,70], which produce hydrogen peroxide as a signaling molecule, the present results can be interpreted as showing a functional inhibition of NOX and hydrogen peroxide. Notably, hydrogen peroxide has a dual role in organisms [43,44,45,46,47,48,49,50,51,52]. It is produced as a byproduct of mitochondrial oxidative phosphorylation, which causes oxidative stress. In this case, hydrogen peroxide must be eliminated quickly before extensive damage to biomolecules occurs. However, hydrogen peroxide is also used as a signaling molecule at low concentrations. It is this type of hydrogen peroxide that was inhibited by the NOX inhibitors in this study. Because VAS2870 and other NOX inhibitors were dissolved in DMSO, we also evaluated the effects of DMSO alone.

With respect to wing morphology, DMSO alone had a minor effect, if any. In contrast, VAS2870 in DMSO eliminated a large peripheral portion of the wing proper in severely affected individuals, in both males and females. In severely affected individuals, the MB, SMB, and PFE were all eliminated, and the eyespots were extremely deformed and flattened along the wing edge. These results likely indicate that VAS2870-treated individuals cannot restrict the proper region of apoptotic degeneration and that in normal development, hydrogen peroxide from NOX may define the wing border and thus wing morphology. The present results likely indicate that the large wing area proper, including the presumptive eyespot field, underwent apoptosis in response to VAS2870, together with the further peripheral portion of the wing tissue that normally undergoes apoptosis. It is known that VAS2870 induces or inhibits apoptosis depending on the cellular context; VAS2870 enhances the apoptosis of tumor cells [60] but reduces the apoptosis of platelets [61]. Because VAS2870 is an inhibitor of NOX that produces hydrogen peroxide, hydrogen peroxide may function to maintain the survival of wing tissue during normal development. In this case, hydrogen peroxide may act as a signaling molecule for cellular survival, preventing epidermal cells from undergoing apoptosis. In the far periphery, which normally undergoes apoptosis, hydrogen peroxide may not be produced for survival.

To confirm the findings on the VAS2870-induced changes above, we further used isuzinaxib and DPI. We found that both isuzinaxib and DPI induced changes in wing morphology and color patterns, as did VAS2870. Furthermore, we performed a rescue experiment, in which hydrogen peroxide was injected into pupae after sandwich treatment with VAS2870. The results indicated that the injection of hydrogen peroxide could not statistically significantly rescue the phenotypic effect of VAS2870. This is not very surprising, because hydrogen peroxide, when injected, may not be able to reach the wing due to immediate quenching with catalase. Moreover, while the possible effect of NOX inhibitors may last for hours or even days, hydrogen peroxide will be quenched relatively quickly. As a signaling molecule, hydrogen peroxide should be produced constantly for a relatively long period of time at low concentration levels. Nonetheless, there were fewer wing-deformed individuals in the hydrogen peroxide treatment group than the no treatment group, suggesting that the wing deformation phenotype may be partially rescued. Therefore, the present results, although not statistically significant, are not sufficient to reject the hypothesis that wing deformations are caused by lack of hydrogen peroxide due to NOX inhibitors.

These NOX-inhibitor-induced changes in wing morphology and color patterns are reminiscent of the results of surgical removal of the large peripheral area from the larval imaginal disc, resulting in a flattened eyespot along the wing margin [4,26]. To obtain such an eyespot via surgery, the presumptive eyespot field should be cut out during the larval stage [26]. The surgery results suggest that the eyespot and other color patterns are determined during the late larval stage. However, the present results based on the pupal manipulations demonstrated that the color pattern fates are determined during the early pupal stage but not the larval stage, although the determination process begins in the late larval stage. Moreover, the previous surgery results have been explained by the behavior of a diffusible substance with the eyespot focus and ablated wing edge as either sources or sinks [4,26]. We speculate that the eyespot focus and ablated wing edge are sources or sinks of mechanical forces, according to the physical distortion hypothesis [71]. When the peripheral portion of the pupal wing tissue degenerates in response to a NOX inhibitor, mechanical forces from the prospective eyespot focus and the wing edge may be so close together that they generate a collision to spread these forces out along the wing margin.

A previous study suggested that no qualitative color pattern changes are induced by DMSO via injections into pupae [72]. Although this is probably correct, DMSO quantitatively affected eyespot size in a sexually dimorphic manner. That is, DMSO affected eyespot size in females but not in males. Both the topical and systemic application of DMSO had a negligible effect on eyespots in males. However, in females, DMSO clearly enlarged eyespots, with a 1.48-fold increase in the anterior eyespot and a 1.36-fold increase in the posterior eyespot without affecting the overall color pattern. These results are consistent with other pharmacological studies [73,74]. This eyespot-specific sexually dimorphic effect of DMSO is surprising and worth investigating in the future. In addition, regardless of the sexually dimorphic response to DMSO, this finding is practically important in that many chemical compounds must be dissolved in DMSO as a solvent to test their effects on butterfly color patterns. It is not easy to understand how DMSO changes eyespots in butterflies because DMSO is reported to show various biological effects, including immunomodulation, drug delivery and efficacy modulation, and radioprotection [75,76,77], but we acknowledge that in our experiments, the solvent itself (i.e., DMSO) might have behaved as a ROS scavenger, which might have masked the effects of solutes (i.e., NOX inhibitors) to some extent.

Because the eyespot is determined by morphogenic signals from the eyespot organizer located at the prospective eyespot focus, a larger eyespot induced by DMSO in females indicates that morphogenic signals travel a greater distance. This sexually dimorphic response may not be a trivial discovery regarding the understanding of the molecular mechanisms of sexual dimorphism in butterflies. Importantly, morphogenic signals need a mechanical surface of proper rigidity and hydrophobicity to travel [78]. In this context, thiol modifications of cuticular proteins by DMSO may change the structure of the cuticle’s inner surface, allowing tighter cellular binding to the inner cuticle surface and resulting in eyespot morphogenic signals traveling longer distances, according to the physical distortion hypothesis [71]. Other mechanisms of the DMSO effect that act intracellularly may also be possible, as DMSO can penetrate into cells; however, eyespot enlargement with no effect on the overall color pattern cannot be explained well by the nonspecific intracellular penetration of DMSO.

Extensive eyespot deformation was observed following the topical application of NOX inhibitors in both males and females. This deformation of eyespots may largely be due to deformation of the wing morphology. The disappearance of the MB, SMB, and PFE appeared to have affected the eyespot morphology, indicating that the elemental shape and position are determined by the mutually repulsive interactions among the elements, although the elements are not in physical contact, as indicated in previous studies [23,24,25]. However, eyespots were also deformed without peripheral degradation invading the eyespot area in a few individuals, suggesting that eyespots themselves may employ a mechanism similar to peripheral degradation for color pattern formation. It is known that physical damage causes cell death (necrosis, not apoptosis) and calcium waves, and then wound healing induces ectopic eyespot formation at the damage site in butterfly wings [4,23,24,79,80,81,82].

**Figure 12:**
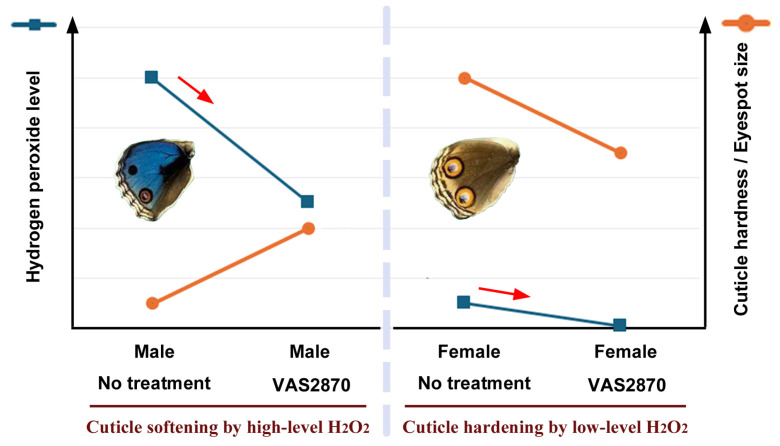
Possible interpretations of the sexually dimorphic response of eyespot size to VAS2870. A key is the dual concentration-dependent effects of hydrogen peroxide. Eyespot size is here considered to be directly related to cuticle hardness, which is controlled by the level of hydrogen peroxide. Red arrows indicate treatments with VAS2870 to reduce the level of hydrogen peroxide.

Systemic application of VAS2870 revealed that eyespot size responded to this compound in a sexually dimorphic manner; eyespots were enlarged in males but were diminished in females. Because the DMSO effect was female-specific for eyespot enlargement, these two compounds (i.e., DMSO and VAS2870) seem to work similarly but in a counteractive manner. To understand the sexually dimorphic results of VAS2870, the possible opposing effects of hydrogen peroxide at relatively high and low concentrations are discussed below (Figure 12). We presume that eyespot size is determined by cuticle hardness [78,83,84] and that hydrogen peroxide has a dual effect on pupal cuticle hardness in a concentration-dependent manner.

At low levels, hydrogen peroxide may crosslink specific biomolecules (e.g., tyrosine residues in proteins) in the extracellular matrix, including cuticular proteins, which hardens the cuticle. As a result, eyespot morphogenic signals can travel long distances, which may be represented in large female eyespots without treatment. When VAS2870 is applied to females, relatively specific crosslinks are not formed due to the scarcity of hydrogen peroxide, and the cuticle becomes less hard, resulting in smaller eyespots. In contrast, in males, the hydrogen peroxide level may be relatively high without treatment. In that case, hydrogen peroxide may nonspecifically oxidize the cuticle, which makes the cuticle soft. As a result, eyespot morphogenic signals cannot travel long distances in small male eyespots without treatment. When VAS2870 is applied to males, nonspecific oxidation is inhibited, and the cuticle becomes harder, resulting in larger eyespots. In this way, sex-dependent responses to VAS2870 may be explained. Note that the enlarged male eyespots are still much smaller than the reduced female eyespots, indicating that males and females exhibit different concentration ranges of hydrogen peroxide. If the explanations above are correct, the sexually dimorphic eyespot size in this species may be explained by the levels of hydrogen peroxide and its effect on cuticle hardening, although other aspects of sexual dimorphism such as background coloration cannot be explained. However, NOX and hydrogen peroxide may have something to do with background color expression. The DPI treatment enhanced the black background color in males and the orange background color in females.

In accordance with this interpretation, hydrogen peroxide is known to degrade polysaccharides such as chitin at high concentrations [85,86]. ROS in general may be recruited to adjust the crosslinking of cuticle components in insects [87,88,89]. Resilin, which is expressed to make the insect cuticle flexible, may also function in association with ROS [90,91,92,93,94]. According to the physical distortion hypothesis [78], mechanical push and pull among epidermal cells in pupal wing tissues function as morphogenic signals, and accordingly, the reception or emission of mechanical signals may require cytoskeletal reorganization of epidermal cells. Indeed, it has been reported that ROS from NOX induce cytoskeletal reorganization [95,96,97].

In addition to the eyespot enlargement in males and reduction in females, the systemic injection of VAS2870 induced notable modifications in the dorsal and ventral forewings in a single female individual. Similar, although minor, expansion of the black band was also observed in two additional VAS2870-treated individuals, suggesting that this type of modification is induced by VAS2870. This modification pattern is reminiscent of that of the temperature-shock (TS) type induced by cold shock [98], tungstate [99], acid carboxypeptidase [100], sulfated polysaccharides [101], and fluorescent brightener 28 [83]. All of these treatments appear to act on the cuticle [83]. These treatments are known to induce black band expansion and eyespot size reduction [83,98,99,100,101]. Indeed, in the VAS2870-induced modified individuals, the black bands expanded on the ventral side of the forewings. Taken together, we speculate that NOX inhibitor treatments may inhibit the production of hydrogen peroxide required for proper cuticle hardening and color pattern formation. However, hydrogen peroxide may also directly activate TRPA1 channel for color pattern formation [74], as observed in other systems [102,103].

## 5. Conclusions

This study showed the effects of NOX inhibitors in wing morphogenesis and color pattern formation in butterflies. These results suggest that NOX (and thus hydrogen peroxide) functions as a morphogenic signaling molecule for butterfly wing morphogenesis and color pattern formation. Molecular mechanisms of sexual dimorphism in butterflies may be partly explained by the activities of NOX, hydrogen peroxide, and cuticle. Further studies are necessary to validate the function of hydrogen peroxide as a regulator of wing morphology and eyespot color patterns. For example, hydrogen peroxide production may be quenched experimentally by several quenchers. The sexually dimorphic effect of DMSO on eyespot size is also interesting, and it is a practically important finding regarding the use of DMSO as a solvent for chemical compounds.

## Data Availability

The original contributions presented in this study are included in the article/Supplementary Material. Further inquiries can be directed to the corresponding author.

## Supplementary Materials

The following supporting information can be downloaded at: https://www.mdpi.com/article/10.3390/insects17030300/s1, Figure S1: Wings of all butterfly samples used for the DMSO injection experiment (a single sibling group); Figure S2: Wings of all butterfly samples used for the VAS2870 injection experiment at 0.69 mM, 6.94 mM, and 13.87 mM (a single sibling group); Figure S3: Wings of all butterfly samples used for the VAS2870 injection experiment at 27.75 mM and 69.37 mM (a single sibling group); Figure S4: Isuzinaxib-treated wings; Figure S5: DPI-treated wings.

## Abbreviations

The following abbreviations are used in this manuscript:

DMSO	Dimethyl sulfoxide
DPI	Diphenyleneiodonium chloride
DUOX	Dual NADPH oxidase
NADPH	Nicotinamide adenine dinucleotide phosphate
NOX	NADPH oxidase
NT	No treatment
MB	Marginal band
SMB	Submarginal band
PFE	Parafocal element
ROS	Reactive oxygen species

## Appendix A

The survival rates for DMSO and VAS2870 are shown in Table A1 below.

**Table A1:** Survival rates in response to chemical treatments.

Trial Number (Sibling Group)	Application	Chemical	Treated	Eclosed (Male)	Eclosed (Female)	Survival Rate (%)
#1	Topical	DMSO only	36	16	12	77.8%
#2	Topical	VAS2870 (13.87 mM)	15	6	6	80.0%
	Topical	VAS2870 (69.37 mM)	35	20	10	85.7%
#3	Systemic	DMSO only	33	15	15	91.0%
	--	No treatment	31	13	18	100%
#4	Systemic	VAS2870 (0.69 mM)	39	12	18	76.9%
	Systemic	VAS2870 (6.94 mM)	39	17	15	82.1%
	Systemic	VAS2870 (13.87 mM)	38	18	13	81.6%
	--	No treatment	71	32	31	88.7%
#5	Systemic	VAS2870 (27.75 mM)	43	9	7	37.2%
	Systemic	VAS2870 (69.37 mM)	40	1	1	5.0%
	--	No treatment	63	24	25	79.7%
#6	Topical	Isuzinaxib (200.47 mM)	27	17	10	100%
	Topical	DPI (41.32 mM)	36	4	3	19%
	Topical	DPI (4.13 mM)	41	22	14	98%
	Topical	DPI (0.41 mM)	12	6	6	100%
	--	No treatment	4	2	2	100%
	Topical and Systemic	VAS2870 (13.87 mM) + Hydrogen peroxide (12%)	21	6	15	100%
	Topical and Systemic	VAS2870 (13.87 mM) + Water	24	11	13	100%
