# Supplementary material for: Pharmacological Effects of NADPH Oxidase Inhibitors on Butterfly Wing Morphogenesis and Color Pattern Formation in Junonia orithya
Source: Insects. 2026 Mar 10;17(3):300. doi: 10.3390/insects17030300 (PMC13026668; doi:10.3390/insects17030300)

Insects

Pharmacological Effects of the NADPH Oxidase Inhibitors on Butterfly Wing Morphogenesis and Color Pattern Formation in *Junonia orithya*

Yugo Nakazato, Momo Ozaki, Ryunosuke Suenaga, and Joji M. Otaki

The BCPH Unit of Molecular Physiology, Department of Chemistry, Biology and Marine Science, Faculty of Science, University of the Ryukyus, Nishihara, Okinawa 903-0213, Japan.

Supplementary Figure S1. Wings of all butterfly samples used for the DMSO injection experiment (a single sibling group).

(a) No treatment, male, dorsal side (n = 13)

NO1

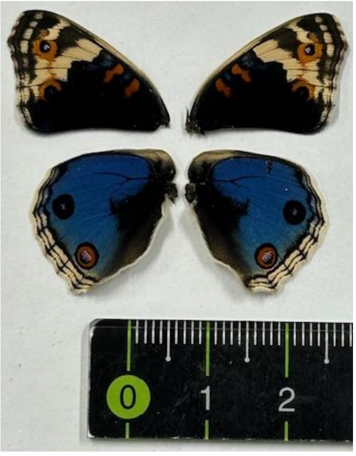

NO2

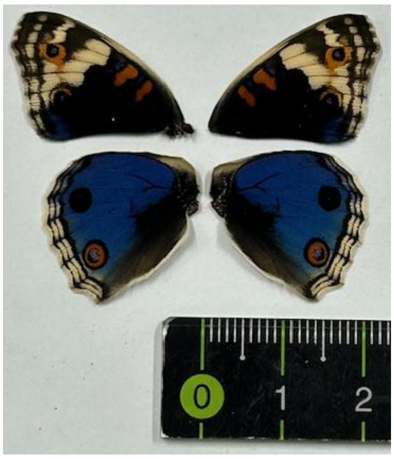

NO3

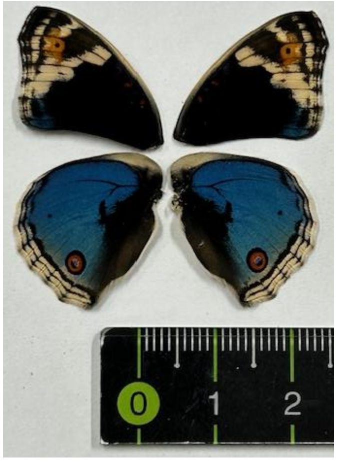

NO4

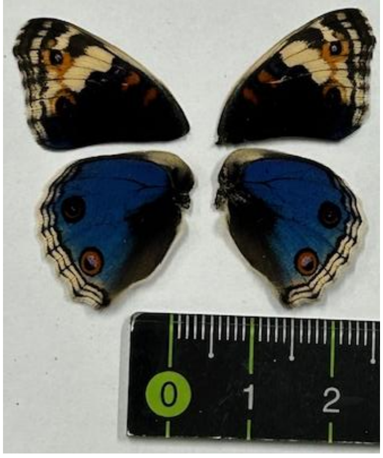

NO5

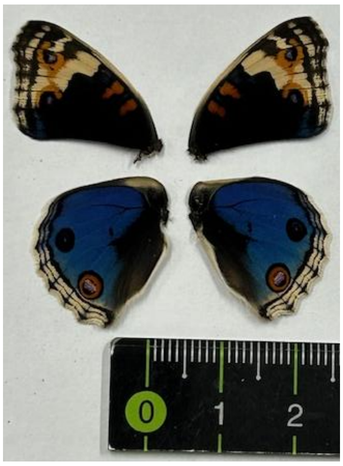

NO6

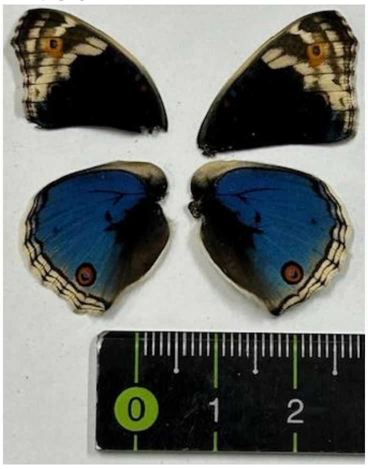

NO7

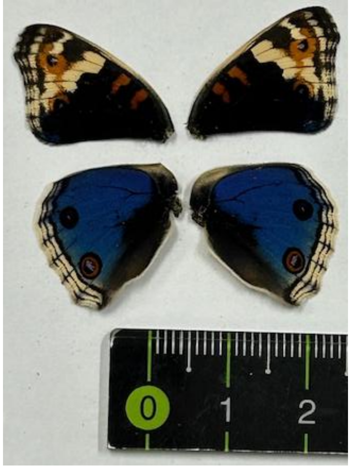

NO8

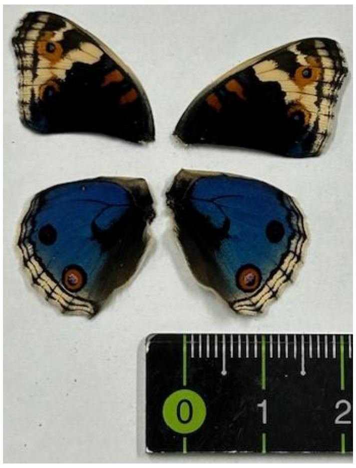

NO9

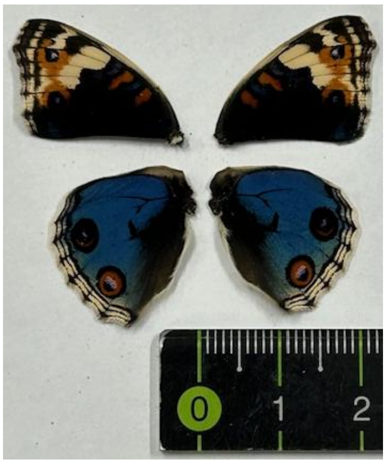

NO10

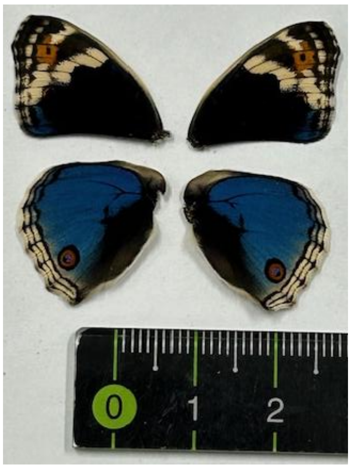

NO11

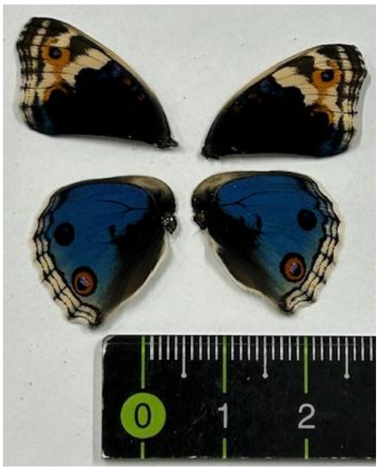

NO12

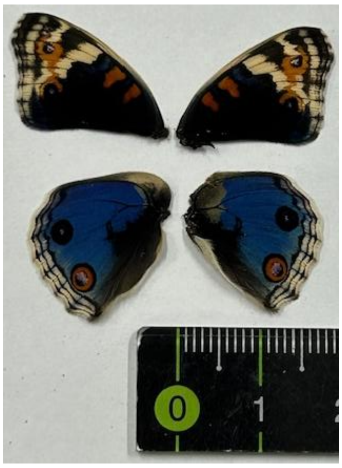

NO13

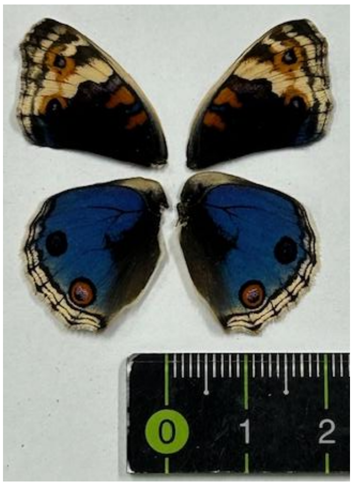

(b) No treatment, male, ventral side (n = 13)

NO1

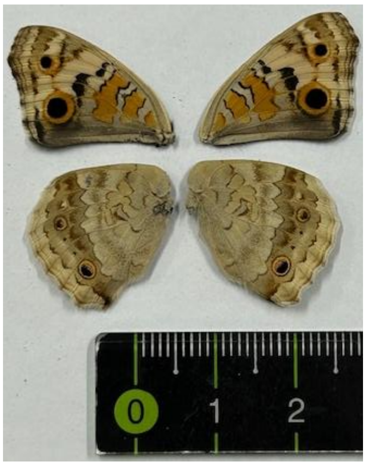

NO2

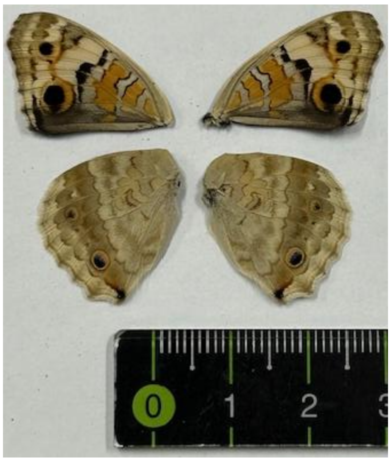

NO3

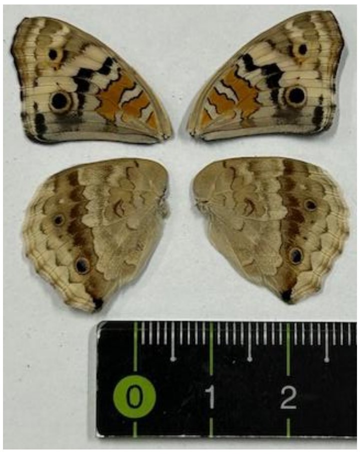

NO4

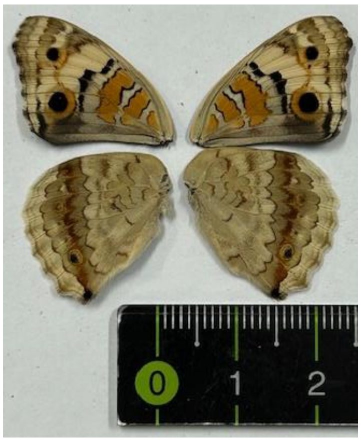

NO5

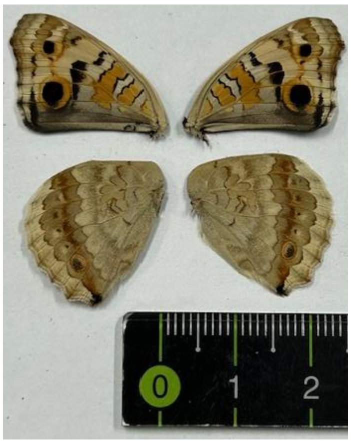

NO6

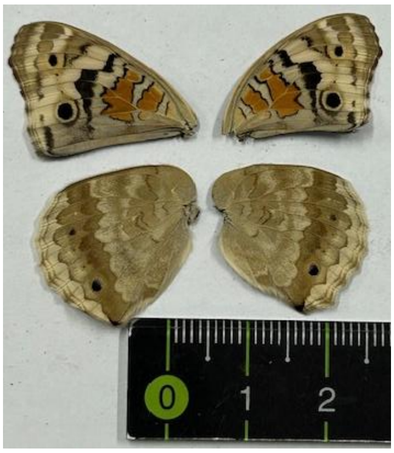

NO7

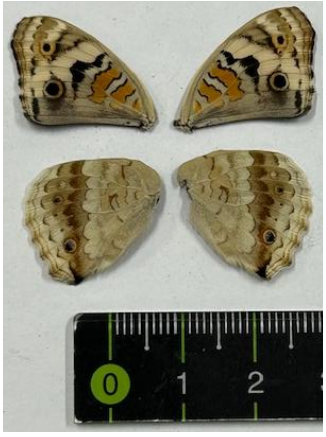

NO8

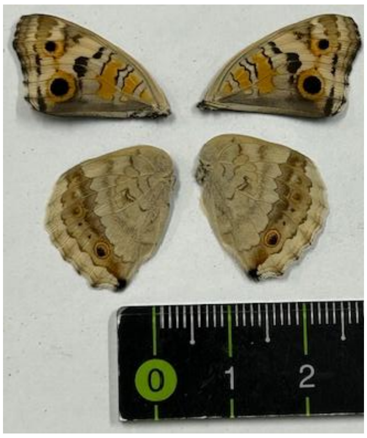

NO9

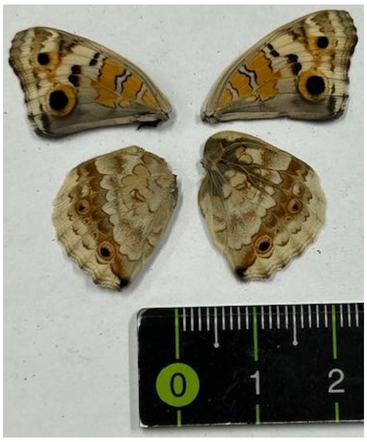

NO10

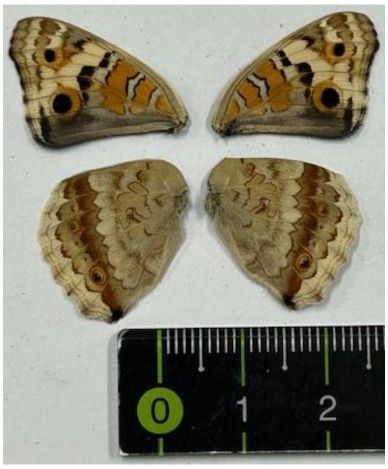

NO11

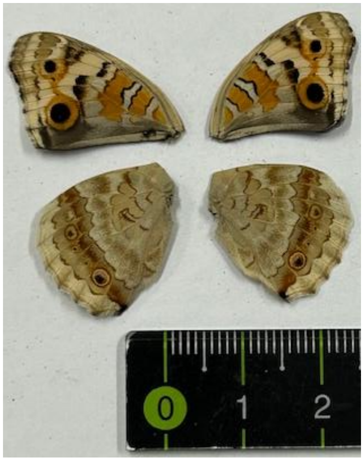

NO12

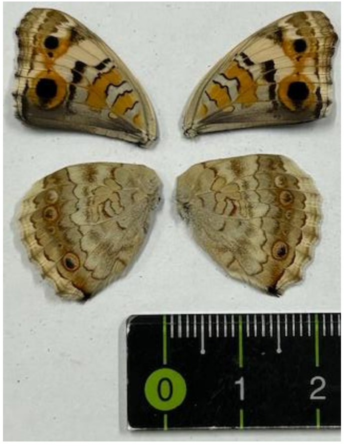

NO13

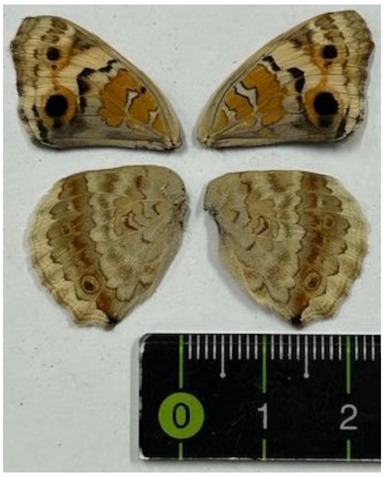

(c) DMSO injection, male, dorsal side (n = 15)

NO1

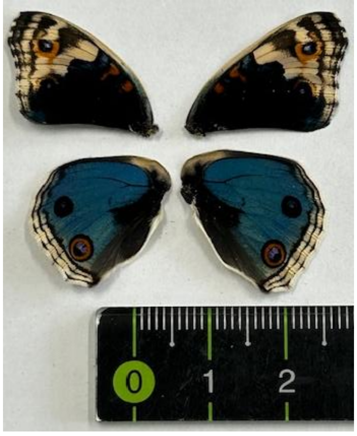

NO2

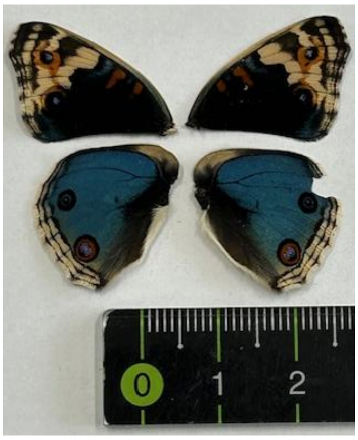

NO3

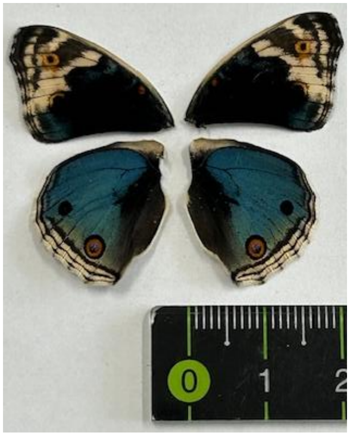

NO4

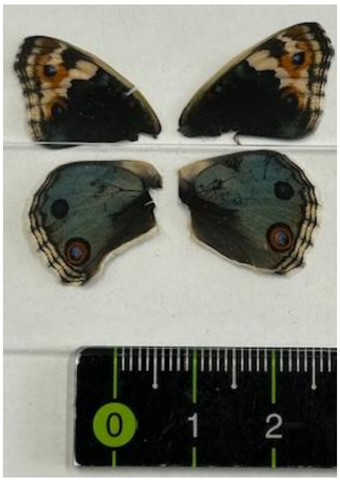

NO5

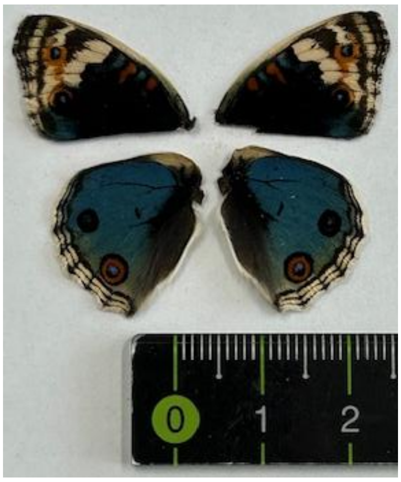

NO6

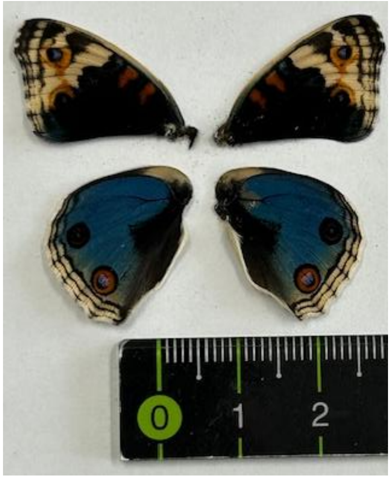

NO7

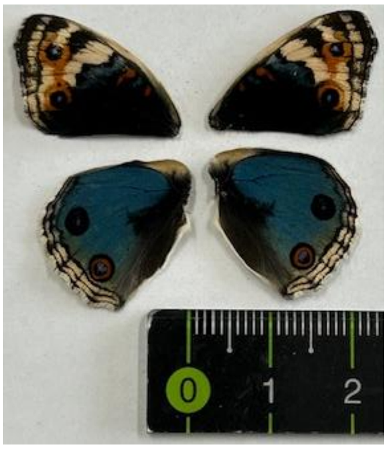

NO8

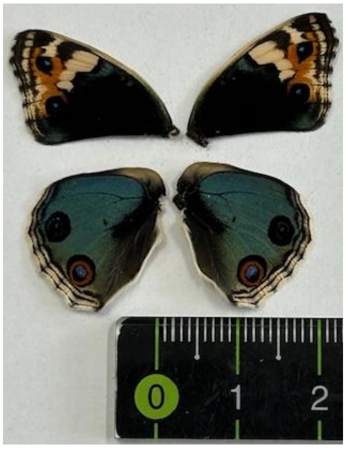

NO9

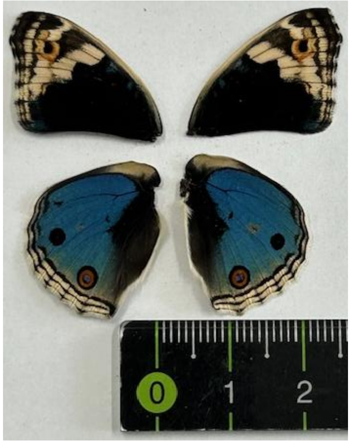

NO10

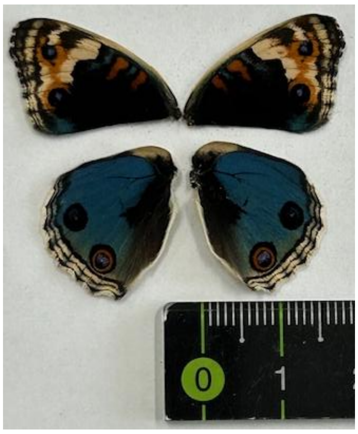

NO11

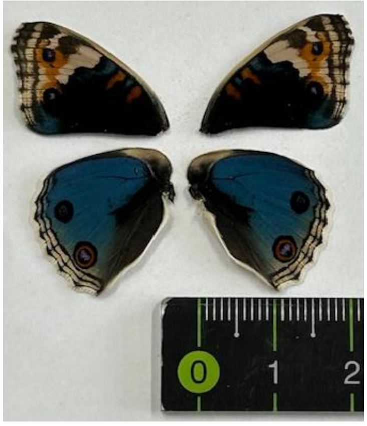

NO12

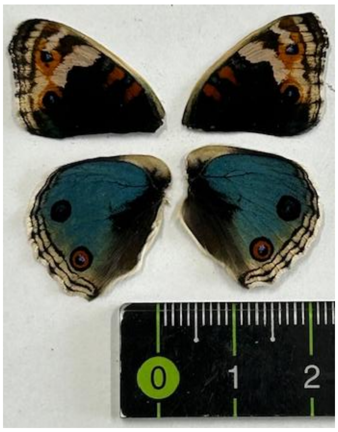

NO13

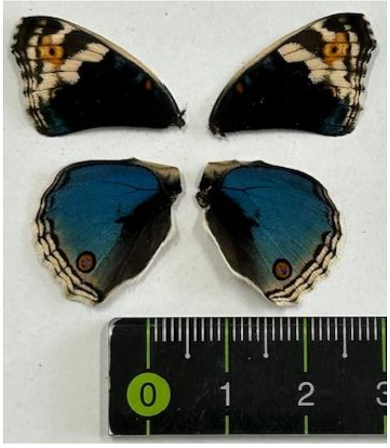

NO14

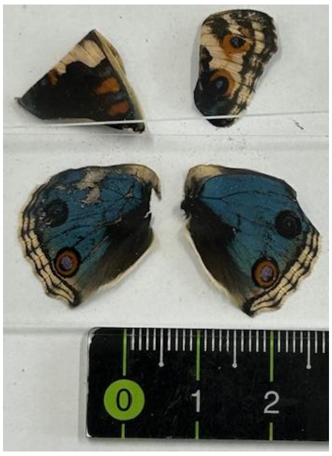

NO15

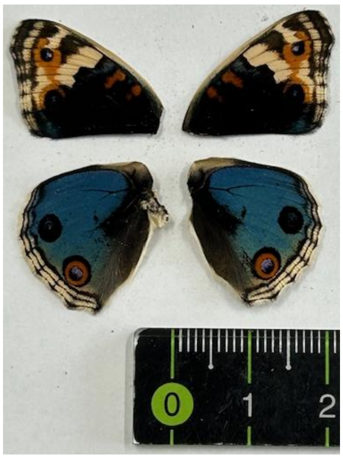

(d) DMSO injection, male, ventral side (n = 15)

NO1

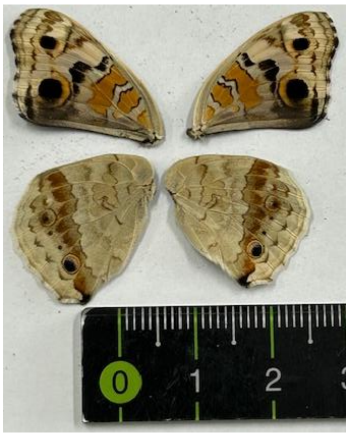

NO2

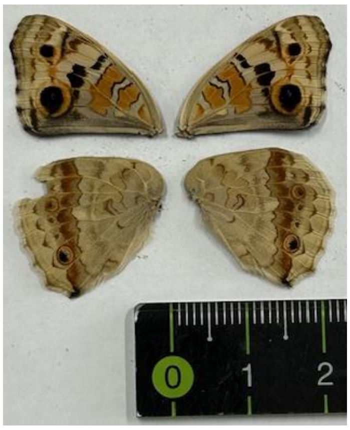

NO3

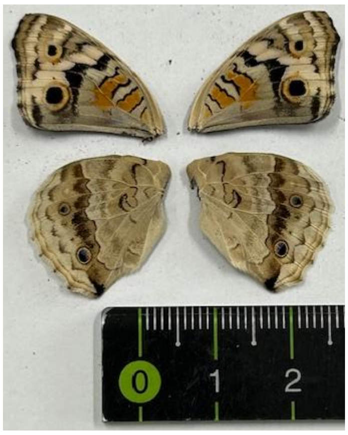

NO4

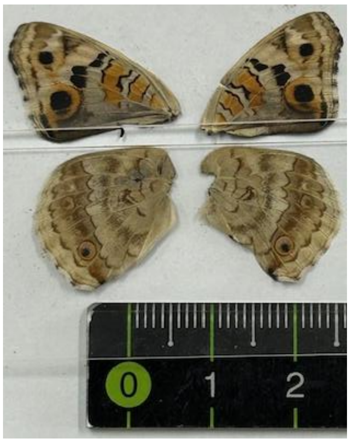

NO5

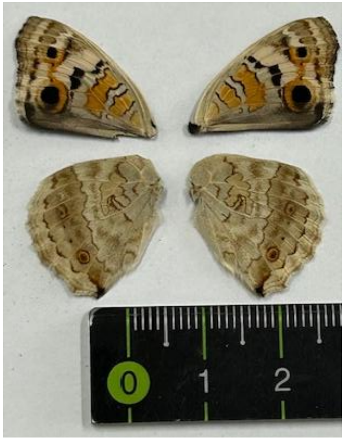

NO6

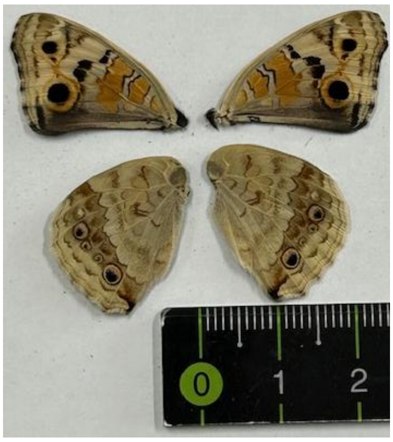

NO7

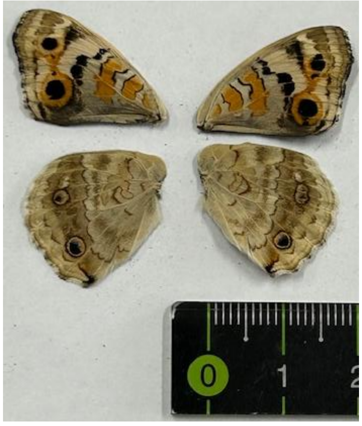

NO8

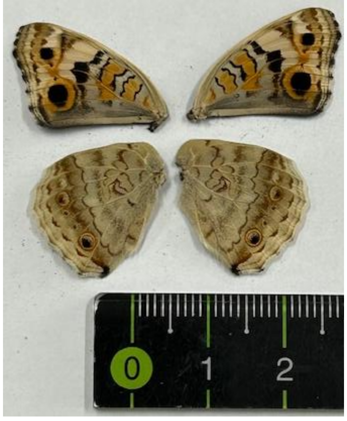

NO9

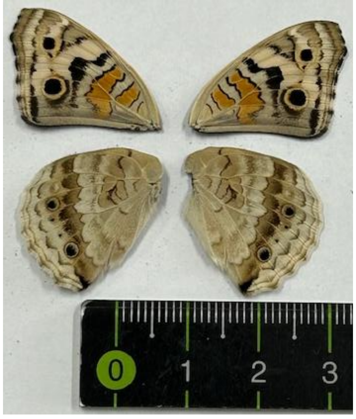

NO10

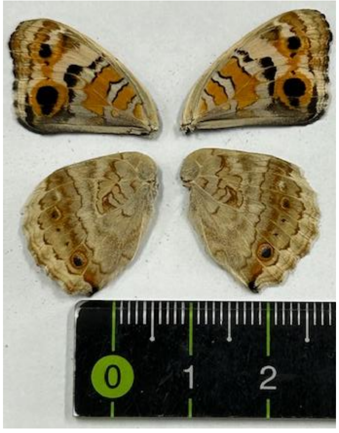

NO11

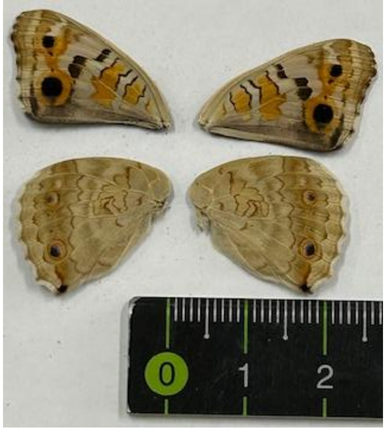

NO12

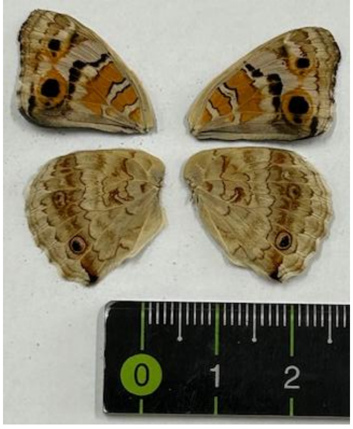

NO13

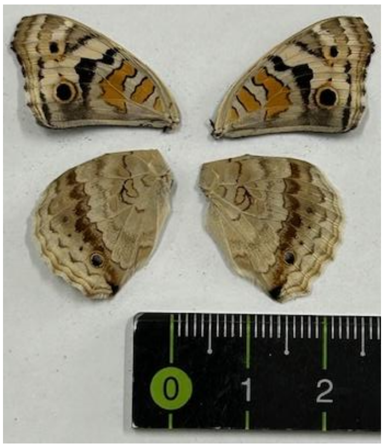

NO14

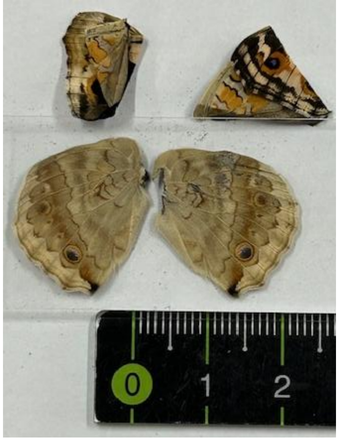

NO15

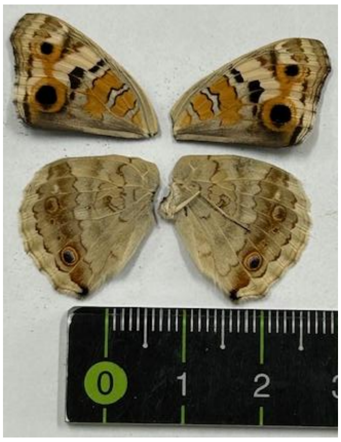

(e) No treatment, female, dorsal side (n = 18)

NO1

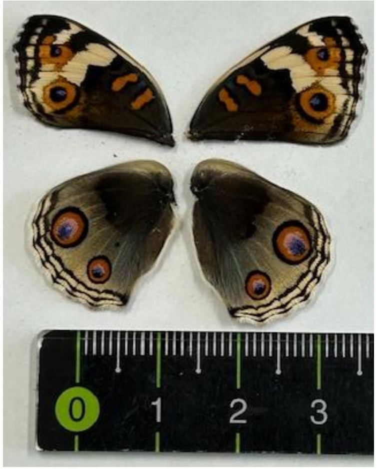

NO2

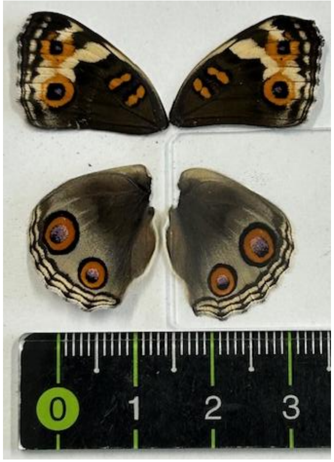

NO3

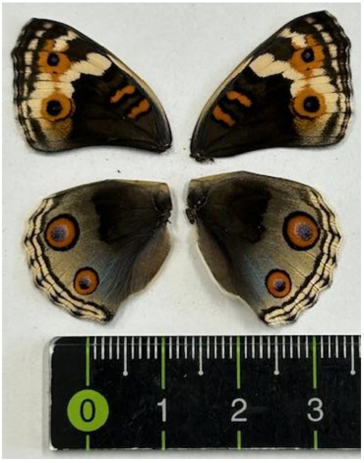

NO4

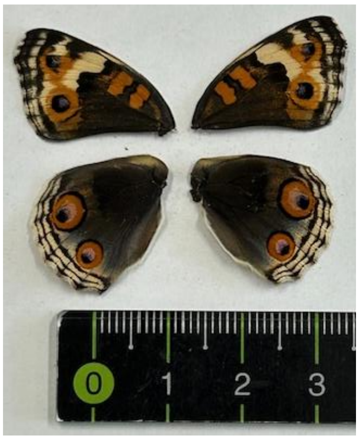

NO5

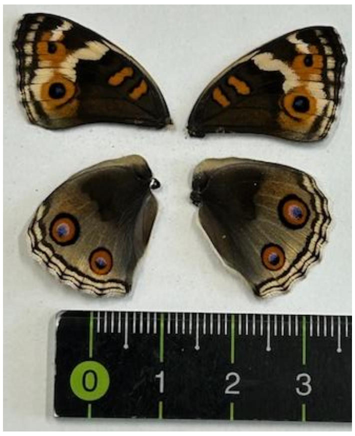

NO6

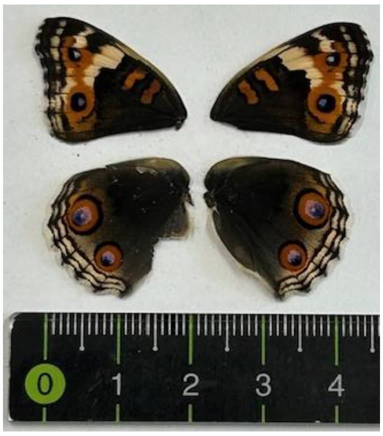

NO7

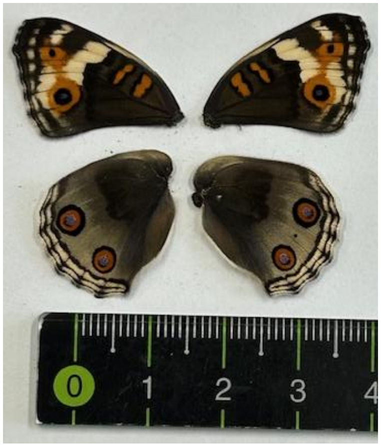

NO8

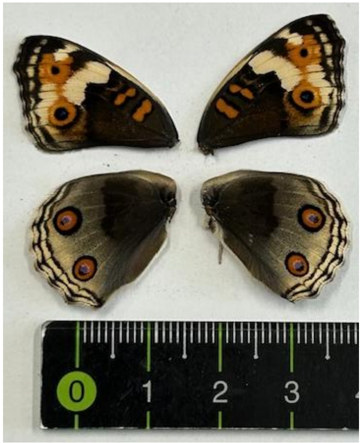

NO9

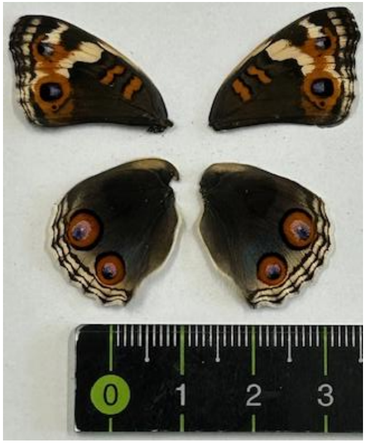

NO10

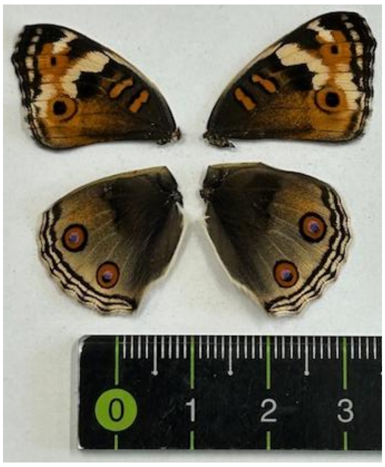

NO11

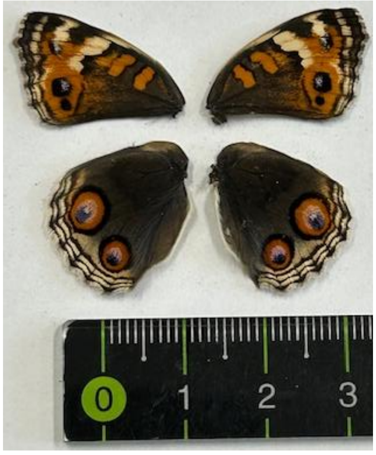

NO12

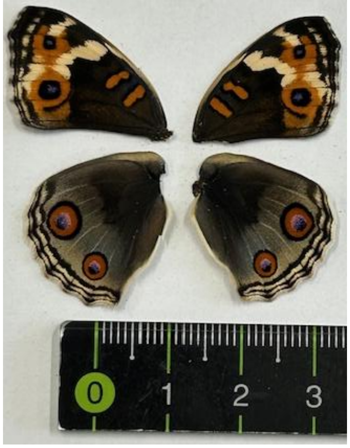

NO13

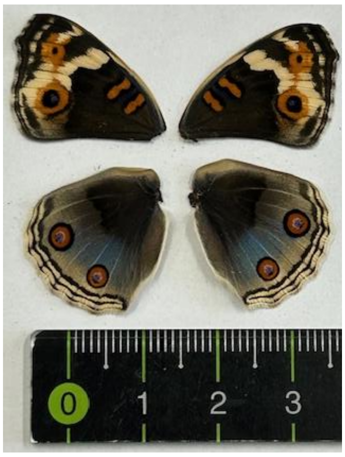

NO14

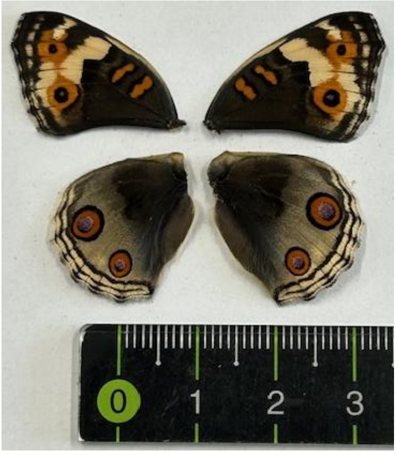

NO15

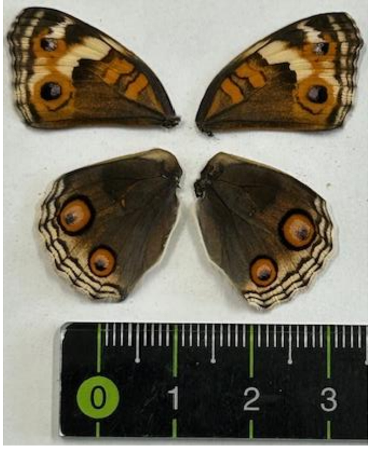

NO16

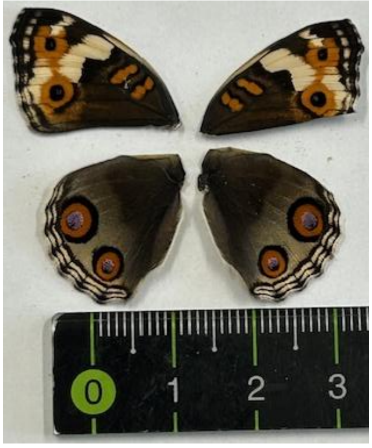

NO17

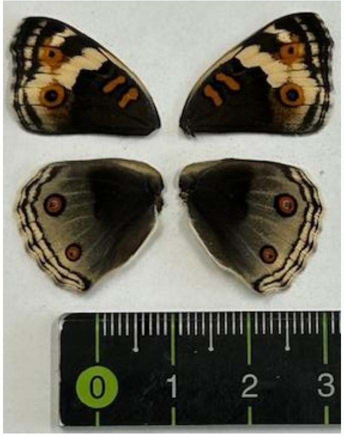

NO18

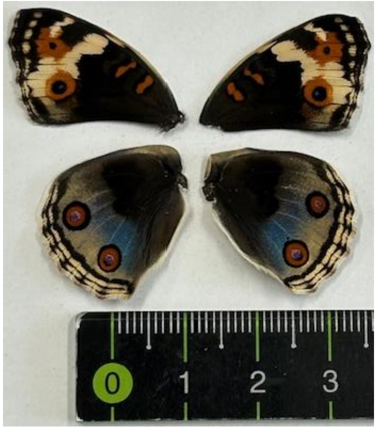

(f) No treatment, female, ventral side (n = 18)

NO1

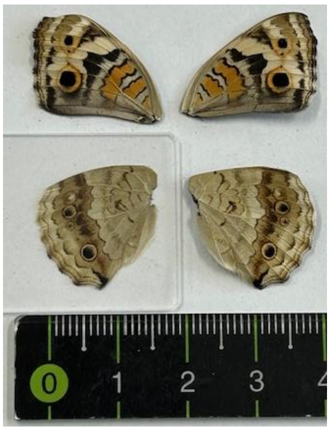

NO2

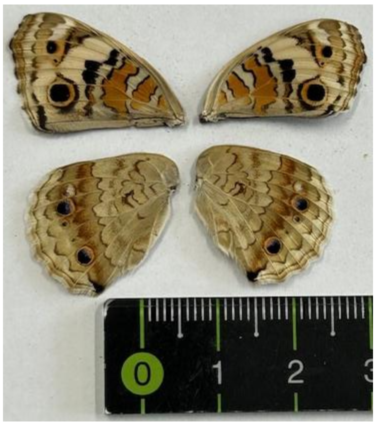

NO3

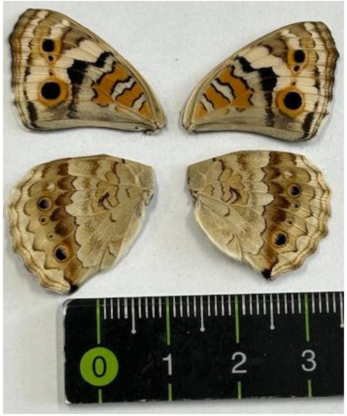

NO4

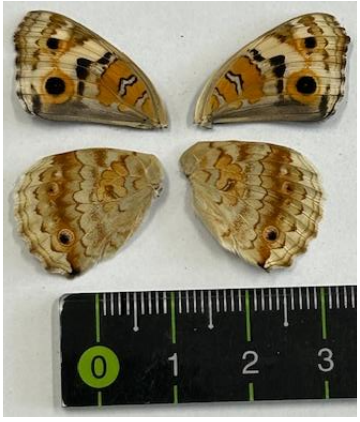

NO5

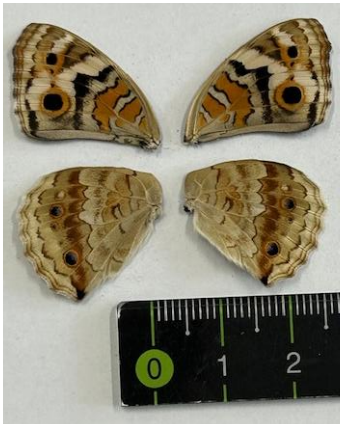

NO6

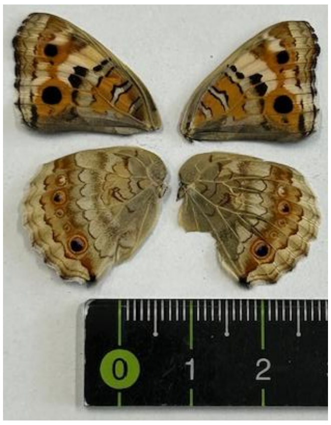

NO7

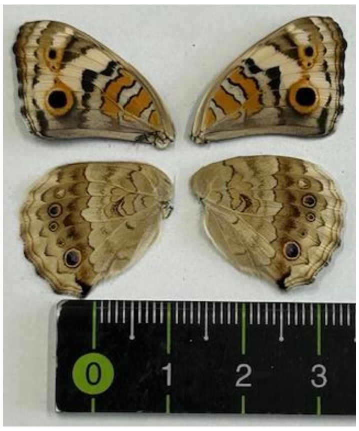

NO8

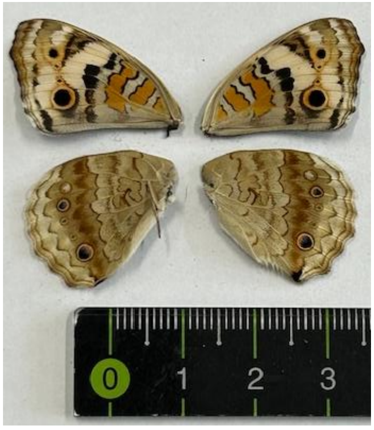

NO9

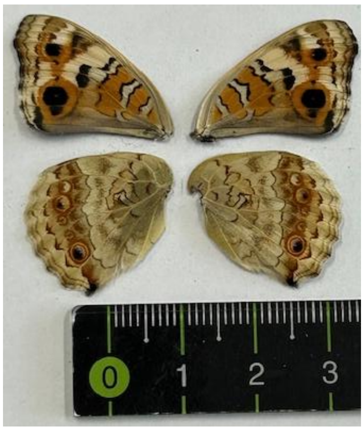

NO10

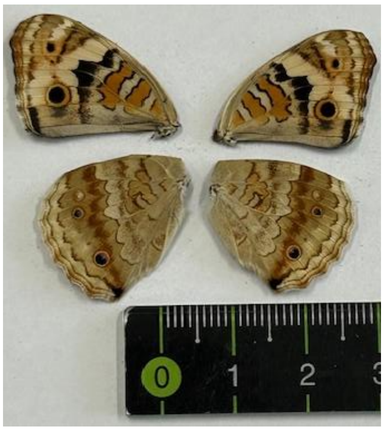

NO11

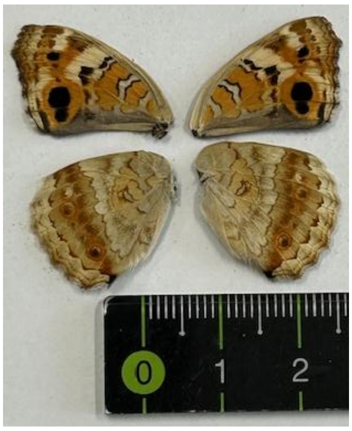

NO12

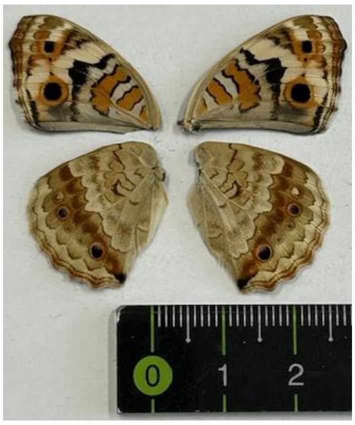

NO13

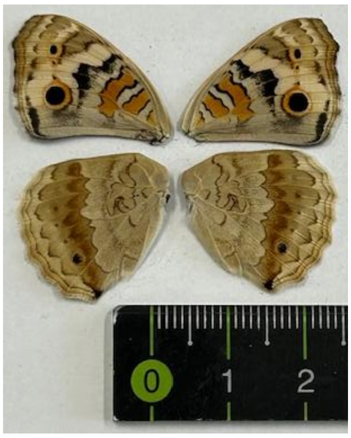

NO14

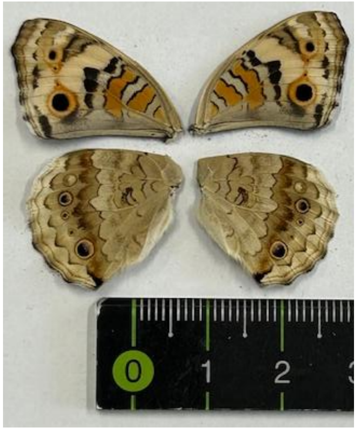

NO15

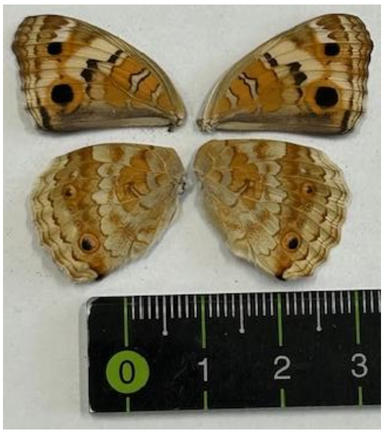

NO16

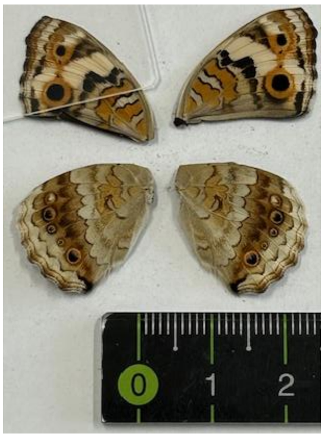

NO17

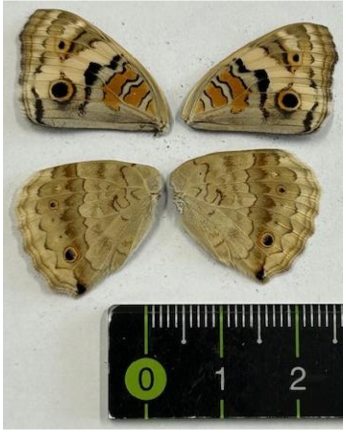

NO18

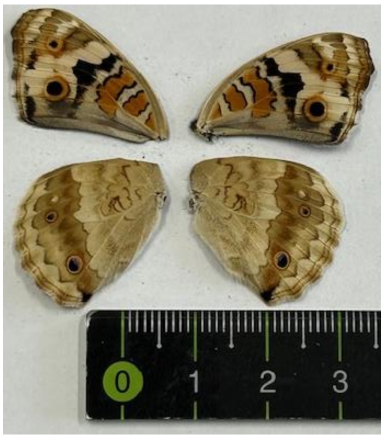

(g) DMSO injection, female, dorsal side ($n = 15$)

NO1

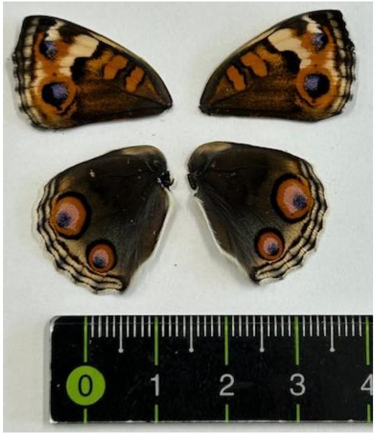

NO2

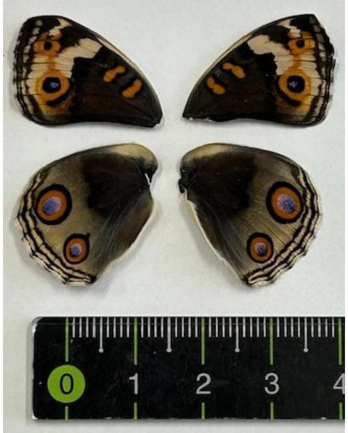

NO3

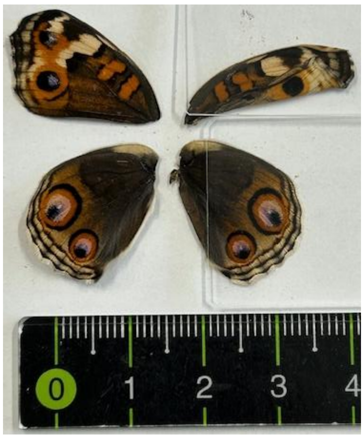

NO4

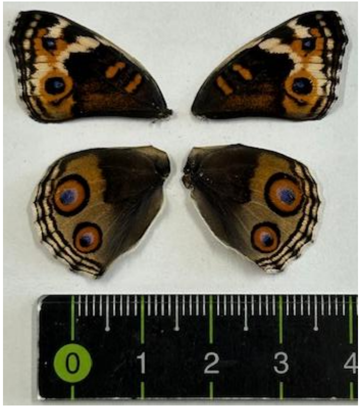

NO5

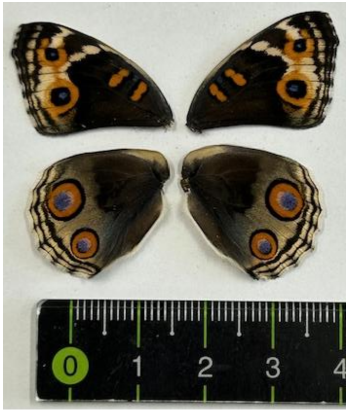

NO6

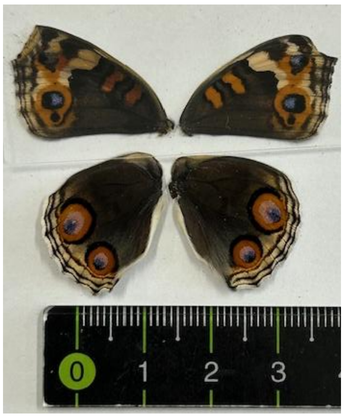

NO7

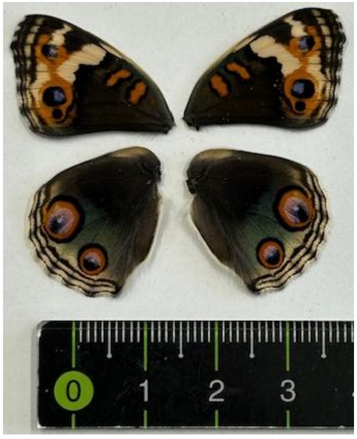

NO8

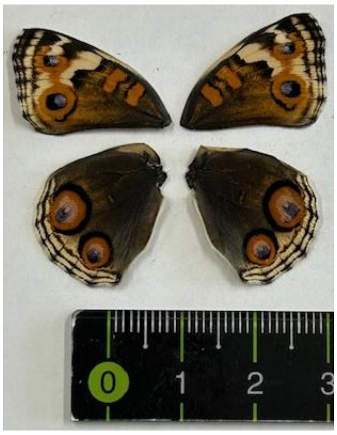

NO9

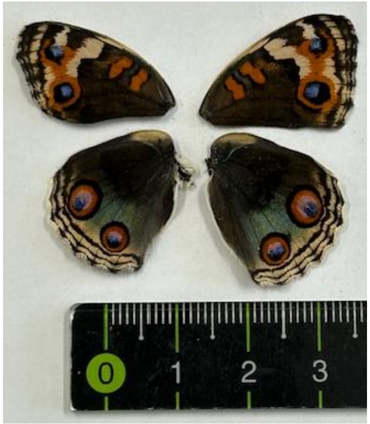

NO10

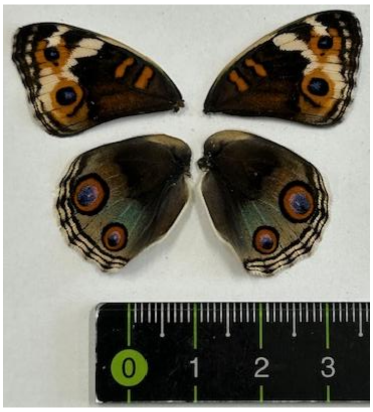

NO11

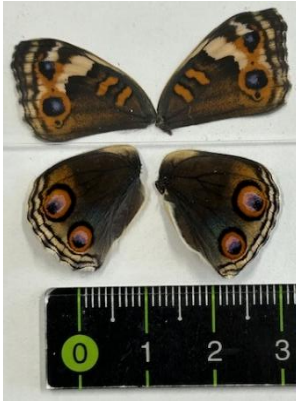

NO12

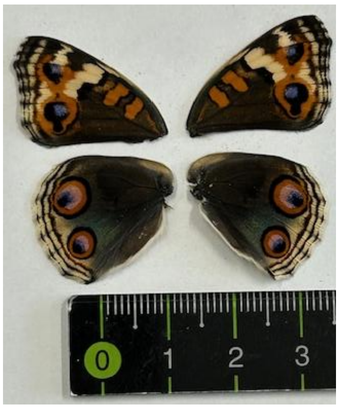

NO13

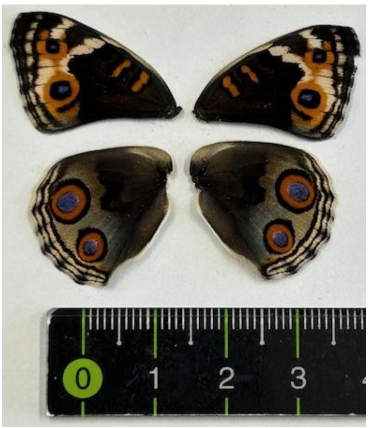

NO14

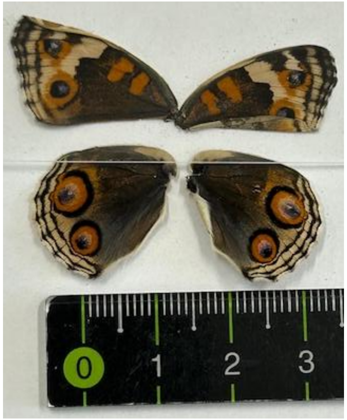

NO15

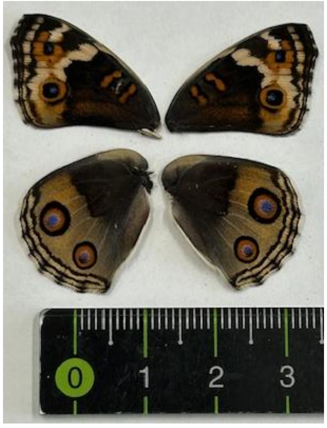

(h) DMSO injection, female, ventral side ($n = 15$)

NO1

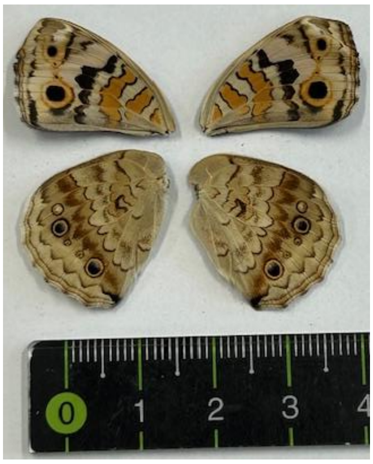

NO2

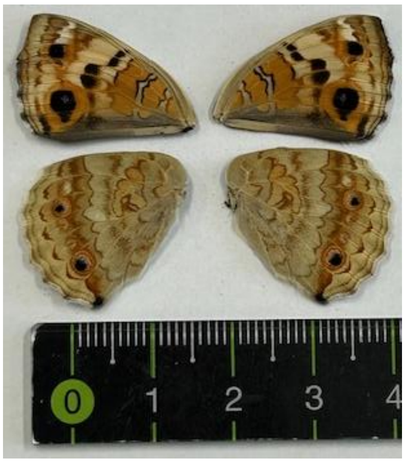

NO3

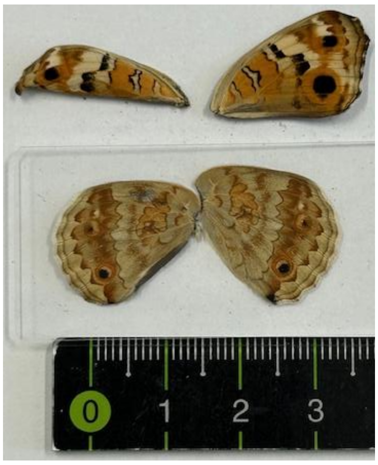

NO4

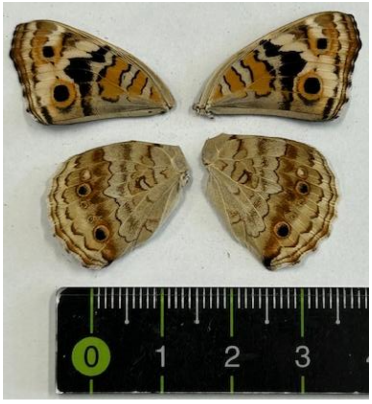

NO5

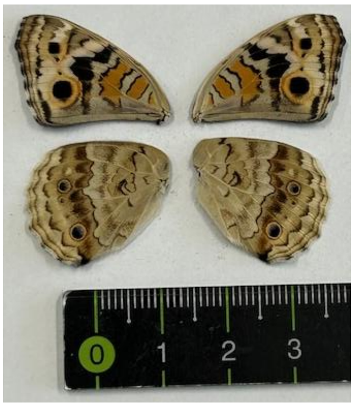

NO6

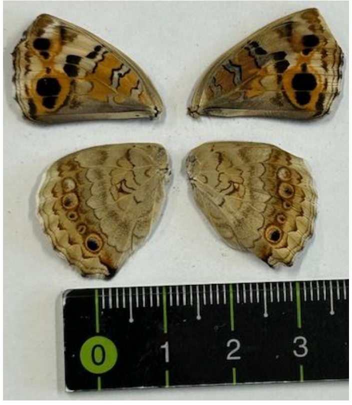

NO7

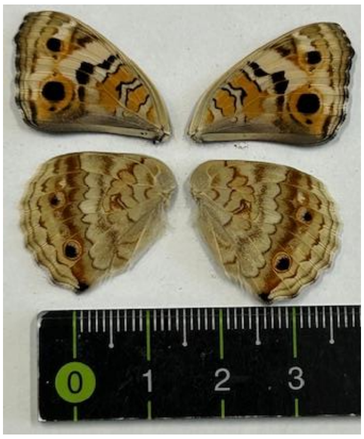

NO8

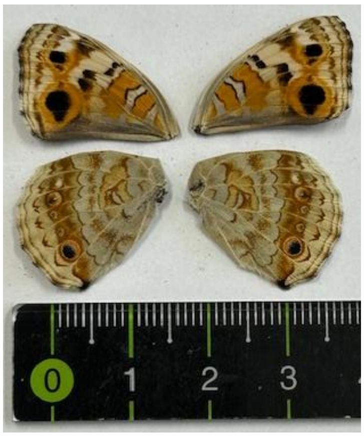

NO9

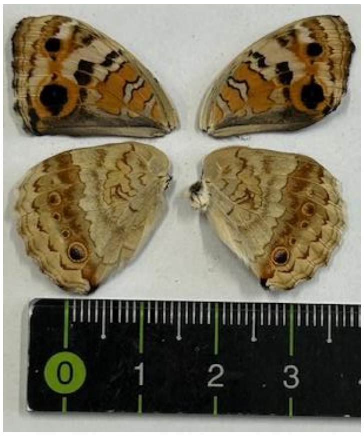

NO10

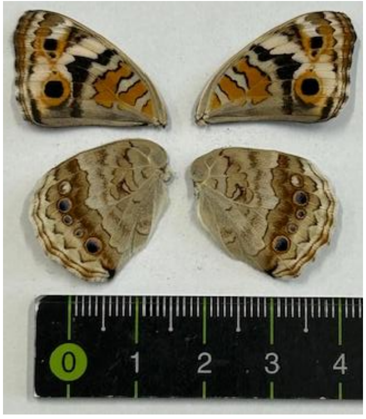

NO11

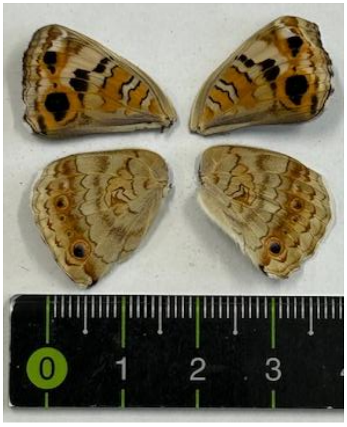

NO12

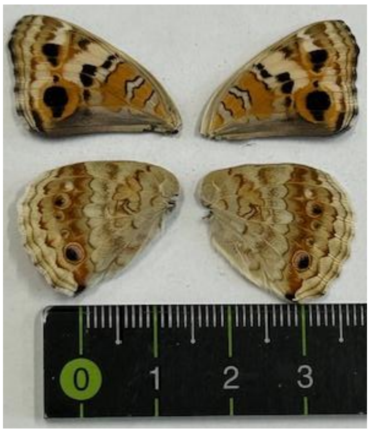

NO13

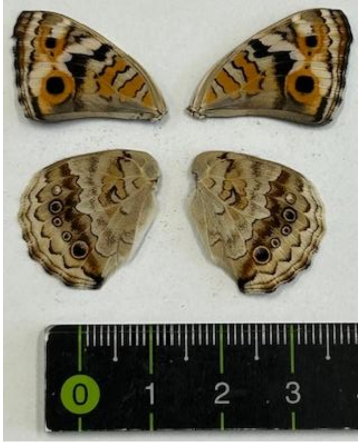

NO14

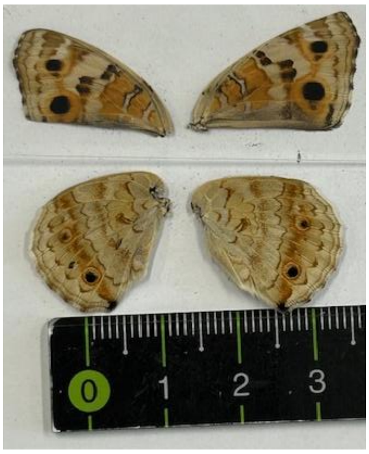

NO15

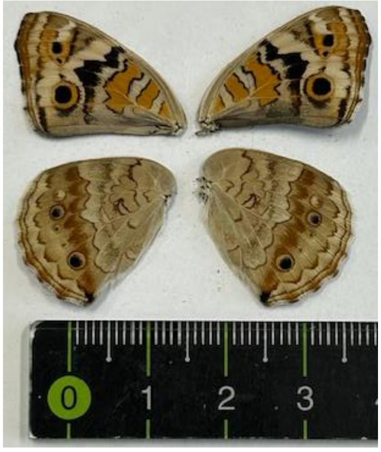

Supplement: Supplementary file 1 [file insects-17-00300-s001.zip › Supplementary Figure S1.pdf]
