# Supplementary material for: Pharmacological Effects of NADPH Oxidase Inhibitors on Butterfly Wing Morphogenesis and Color Pattern Formation in Junonia orithya
Source: Insects. 2026 Mar 10;17(3):300. doi: 10.3390/insects17030300 (PMC13026668; doi:10.3390/insects17030300)

Insects

Pharmacological Effects of the NADPH Oxidase Inhibitors on Butterfly Wing Morphogenesis and Color Pattern Formation in *Junonia orithya*

Yugo Nakazato, Momo Ozaki, Ryunosuke Suenaga, and Joji M. Otaki

The BCPH Unit of Molecular Physiology, Department of Chemistry, Biology and Marine Science, Faculty of Science, University of the Ryukyus, Nishihara, Okinawa 903-0213, Japan.

Supplementary Figure S2. Wings of all butterfly samples used for the VAS2870 injection experiment at 0.69 mM, 6.94 mM, and 13.87 mM (a single sibling group).

(a) No treatment, male, dorsal side (*n* =29)

NO1

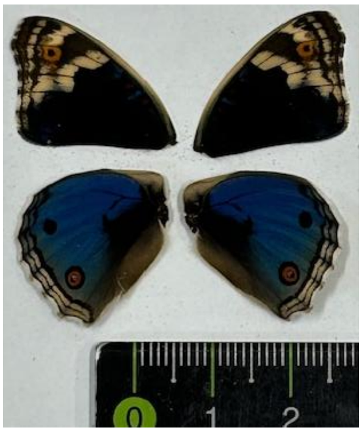

NO2

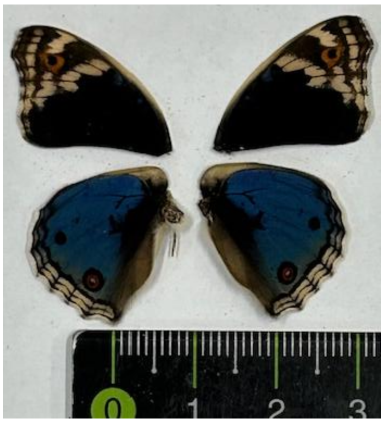

NO3

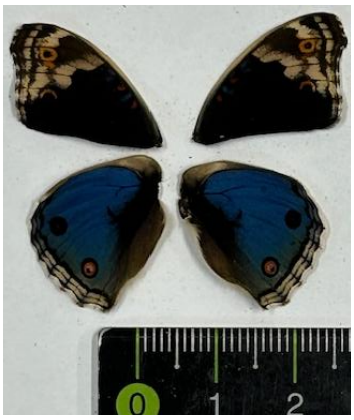

NO4

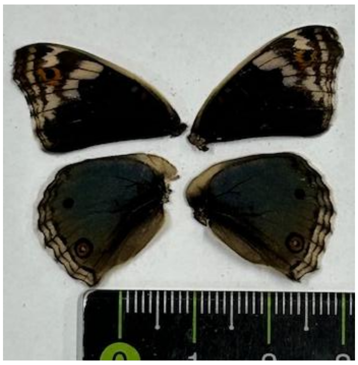

NO5

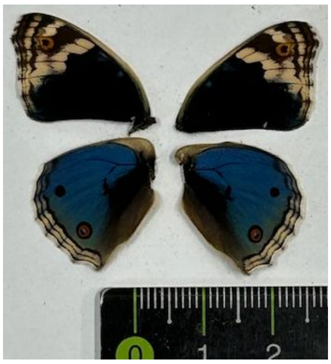

NO6

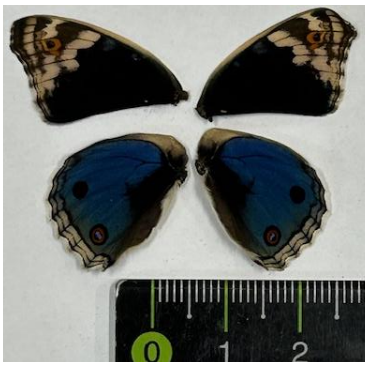

NO7

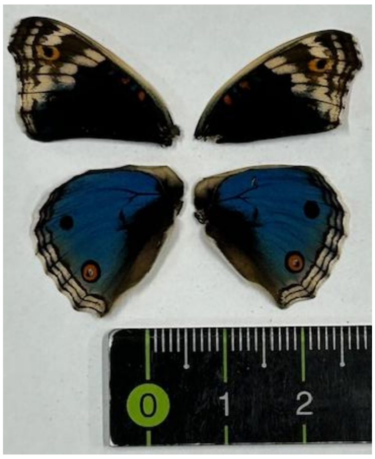

NO8

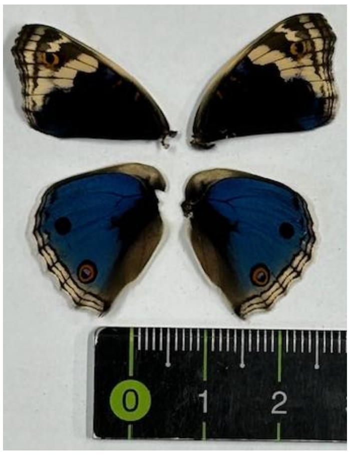

NO9

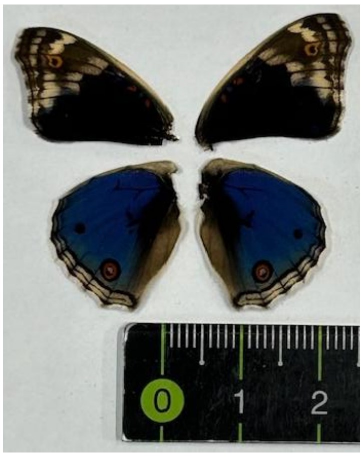

NO10

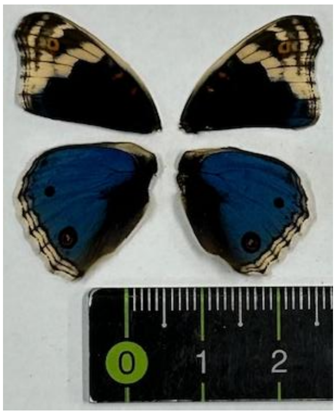

NO11

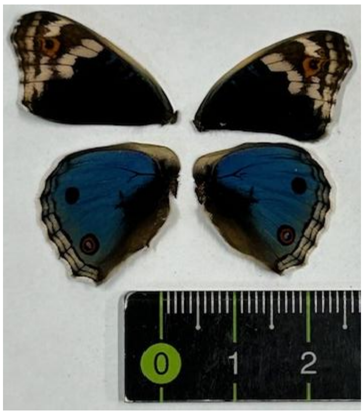

NO12

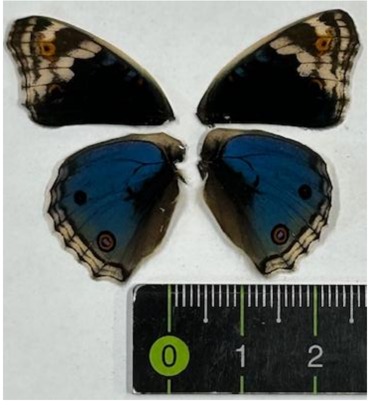

NO13

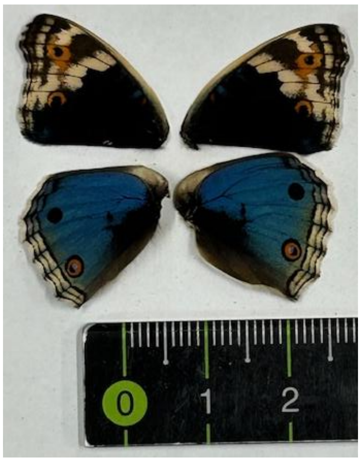

NO14

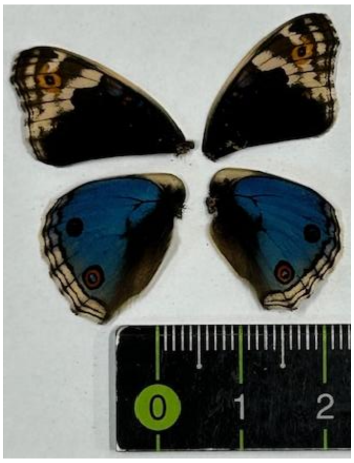

NO15

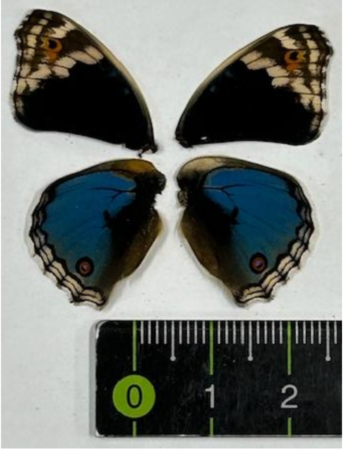

NO16

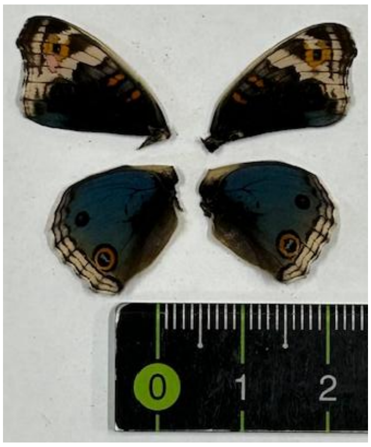

NO17

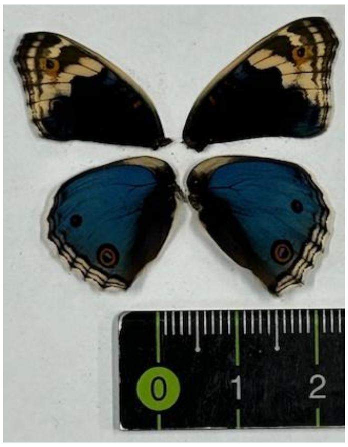

NO18

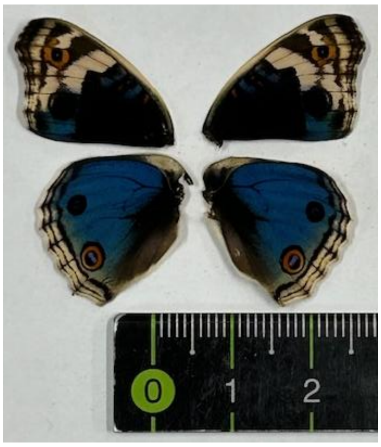

NO19

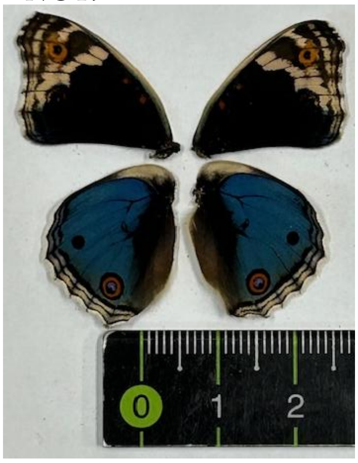

NO20

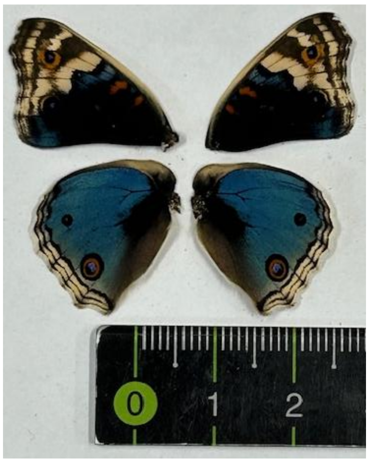

NO21

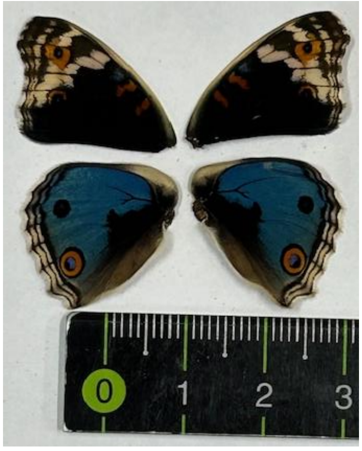

NO22

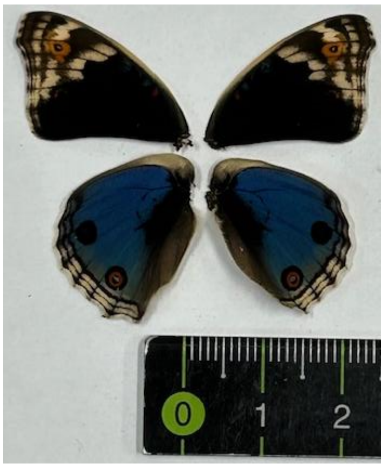

NO23

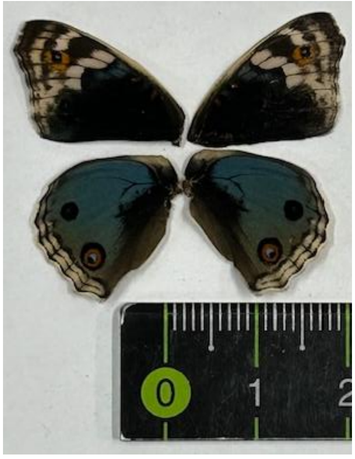

NO24

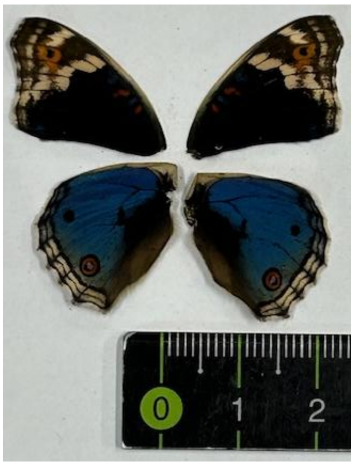

NO25

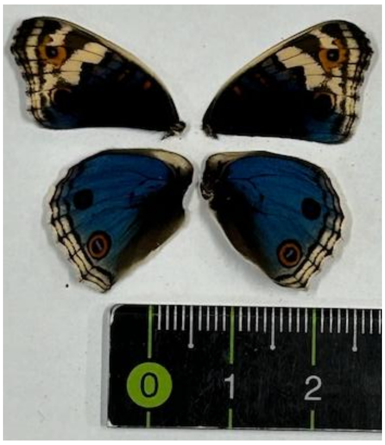

NO26

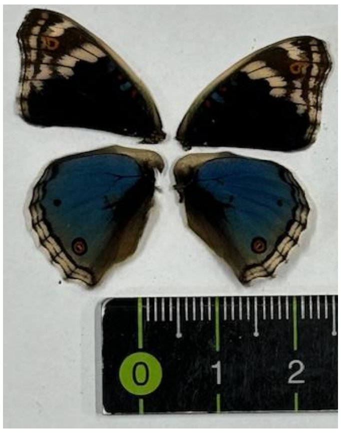

NO27

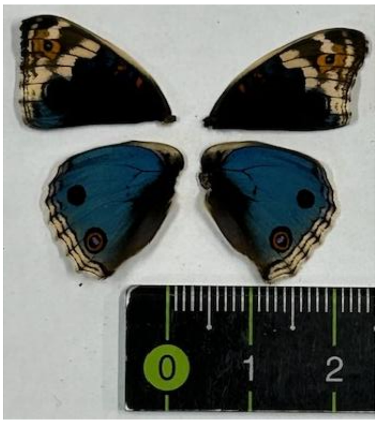

NO28

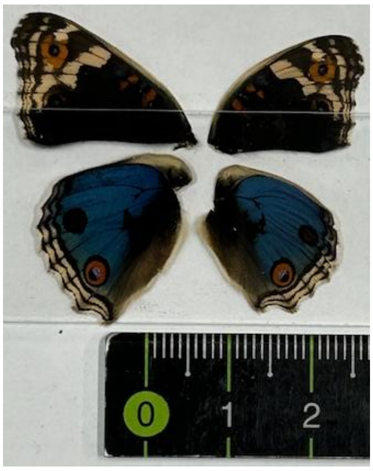

NO29

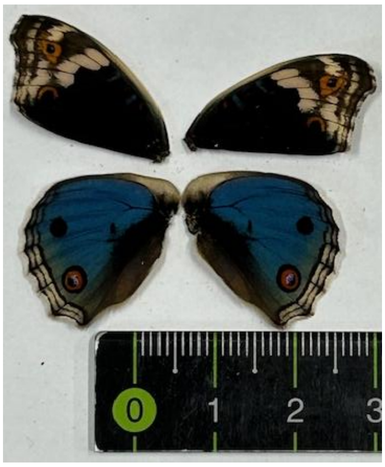

(b) No treatment, male, ventral side ($n = 29$)

NO1

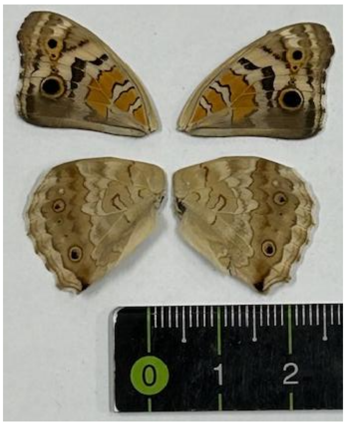

NO2

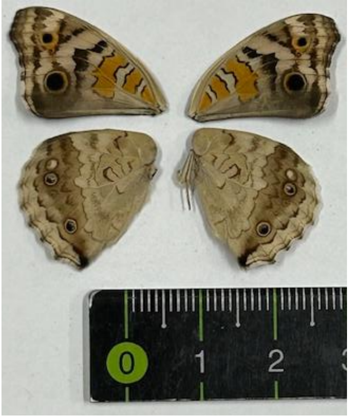

NO3

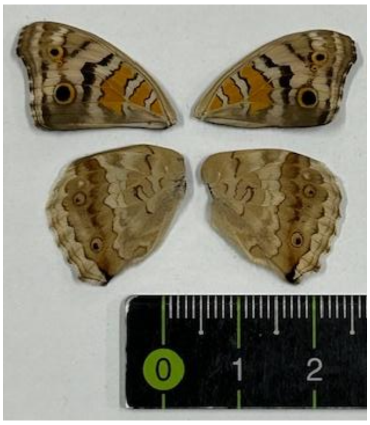

NO4

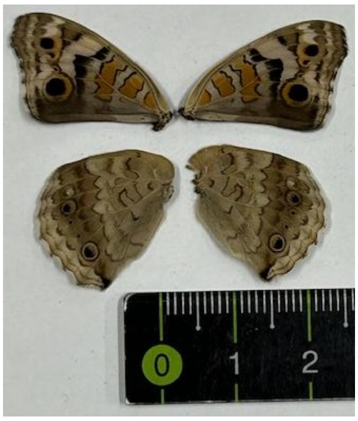

NO5

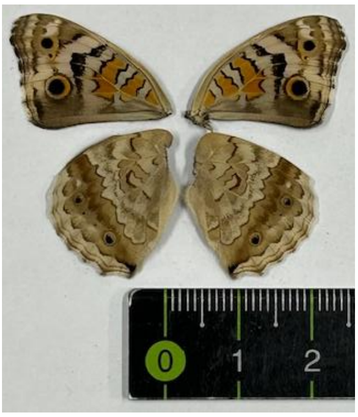

NO6

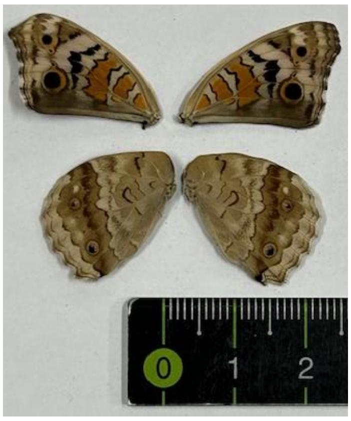

NO7

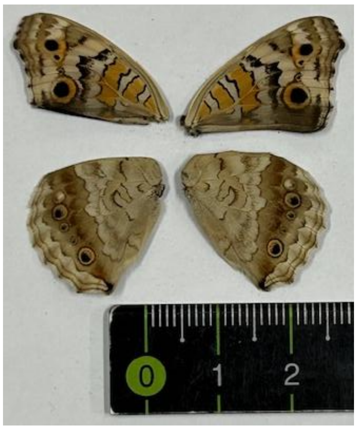

NO8

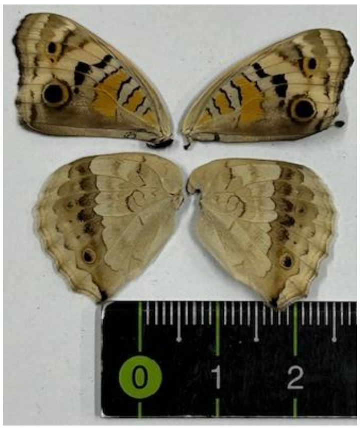

NO9

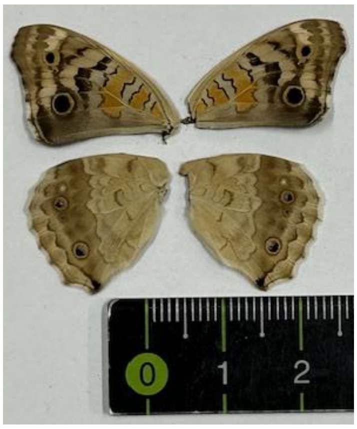

NO10

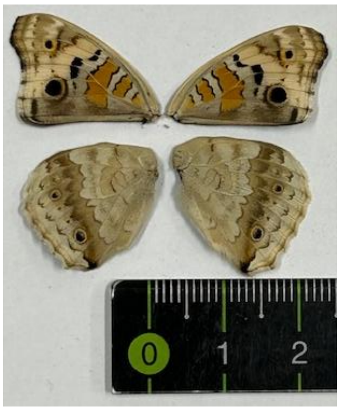

NO11

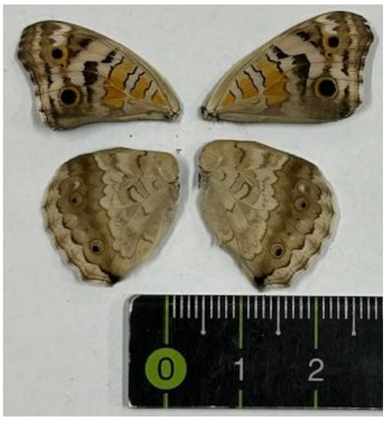

NO12

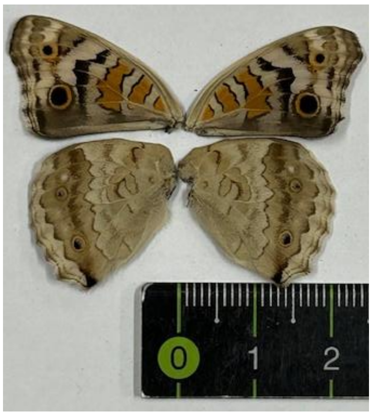

NO13

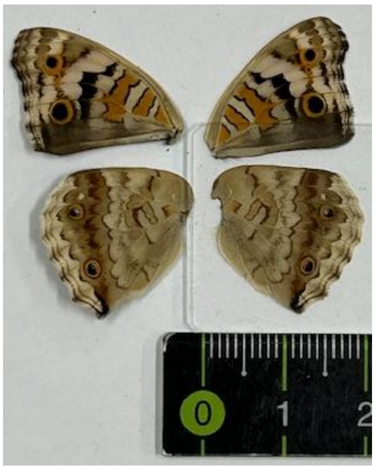

NO14

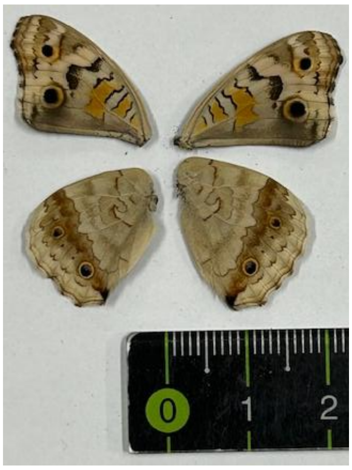

NO15

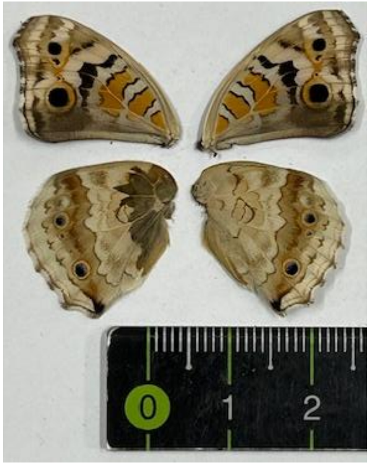

NO16

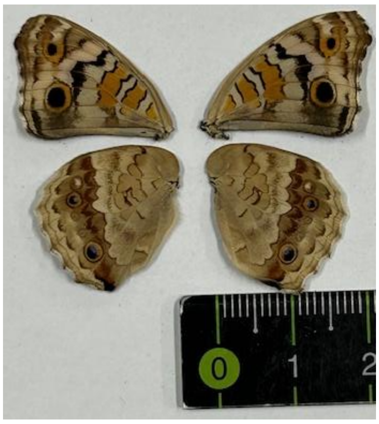

NO17

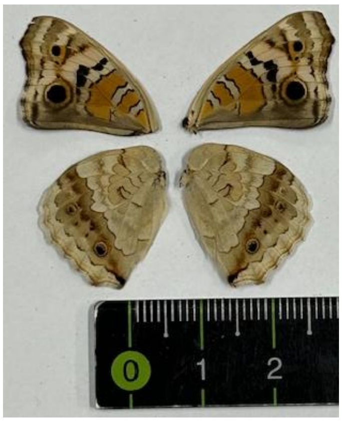

NO18

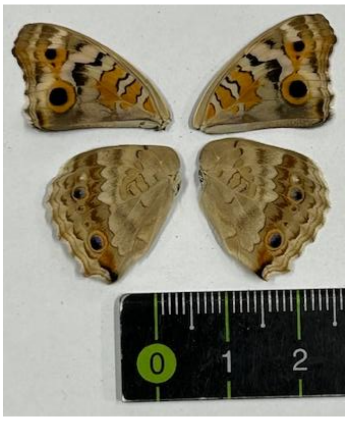

NO19

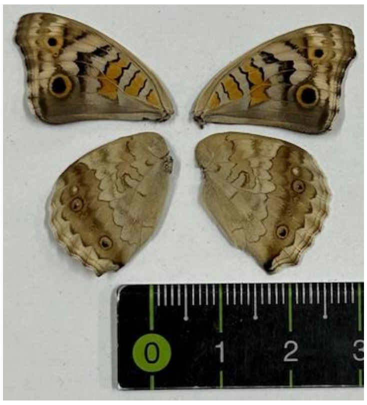

NO20

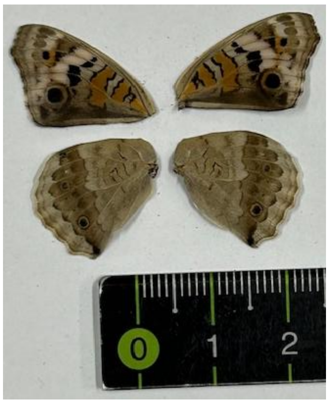

NO21

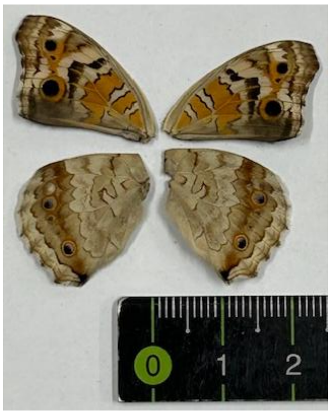

NO22

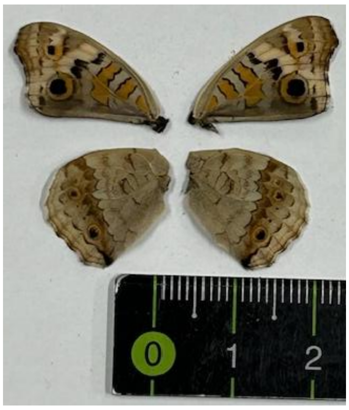

NO23

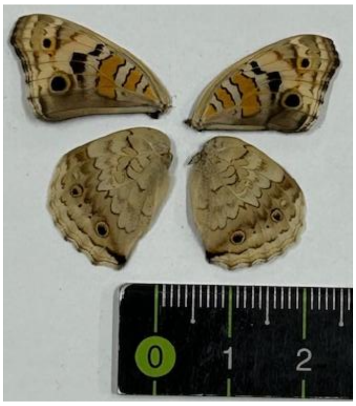

NO24

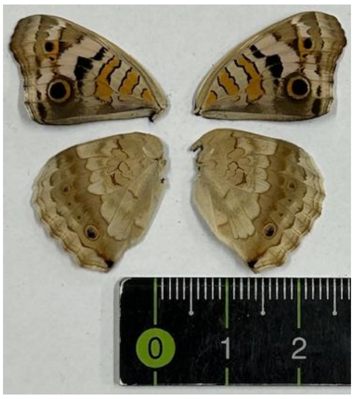

NO25

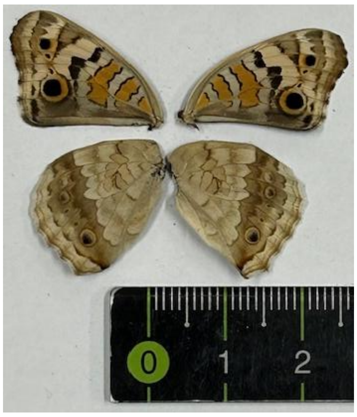

NO26

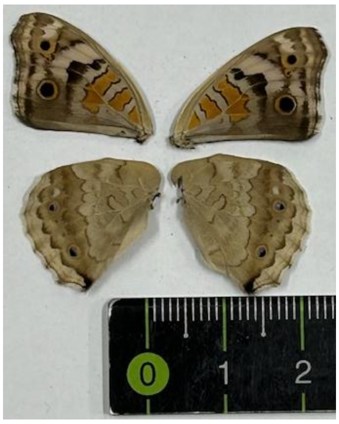

NO27

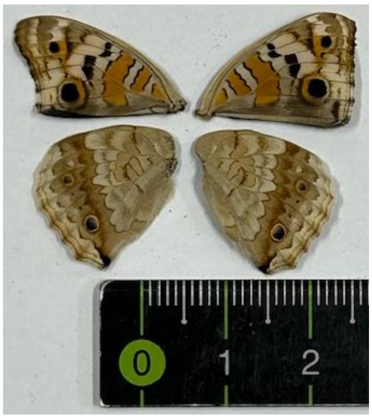

NO28

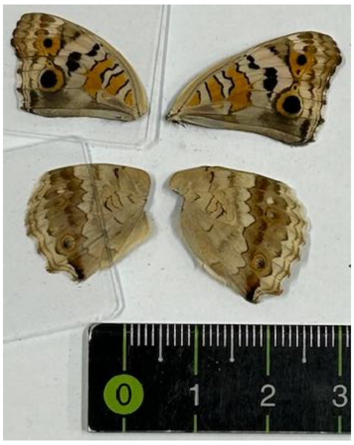

NO29

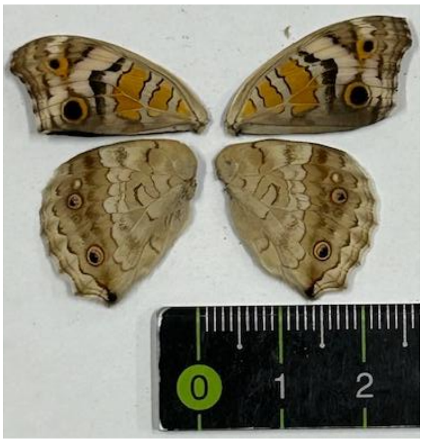

(c) No treatment, female, dorsal side ($n = 30$)

NO1

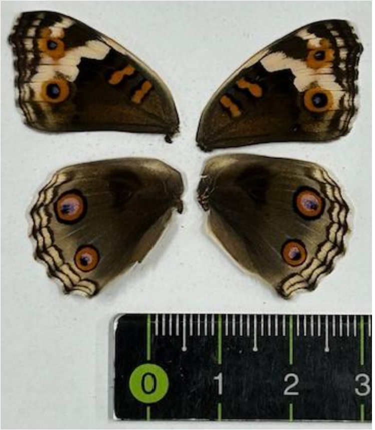

NO2

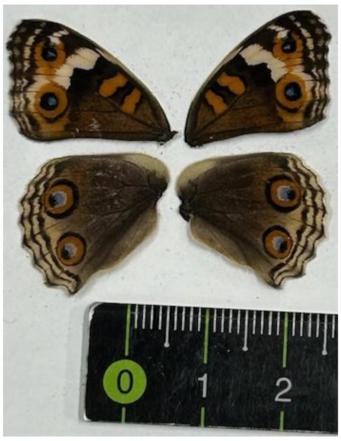

NO3

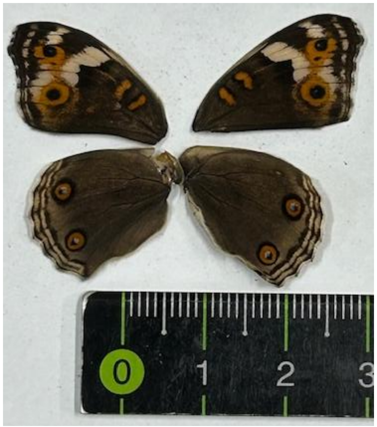

NO4

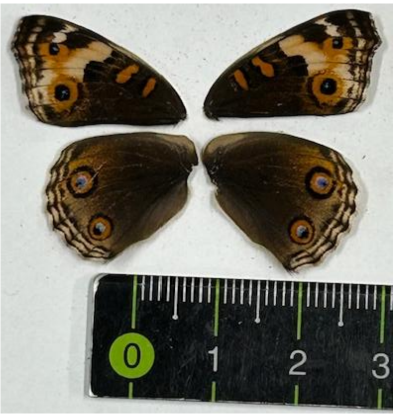

NO5

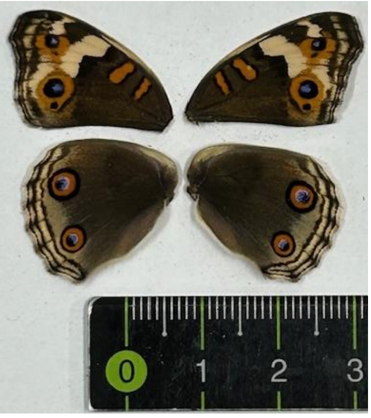

NO6

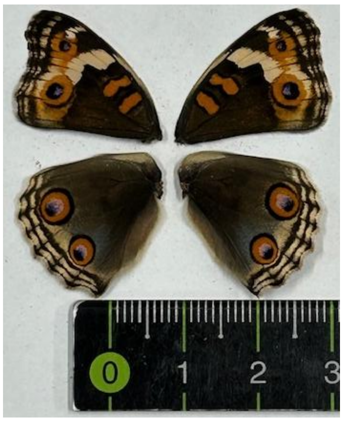

NO7

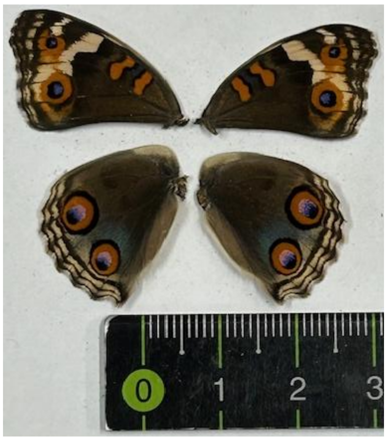

NO8

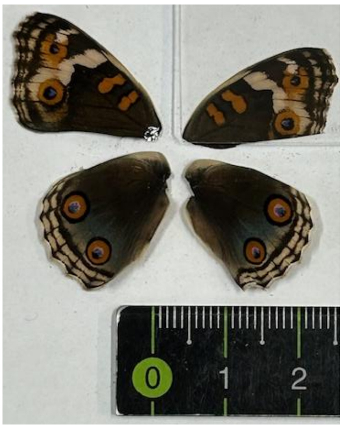

NO9

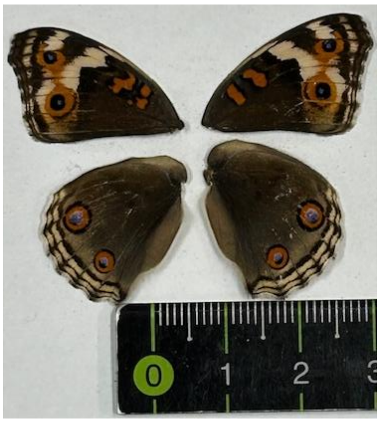

NO10

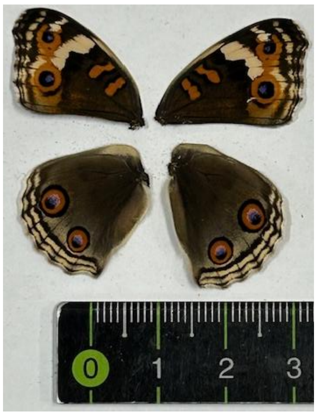

NO11

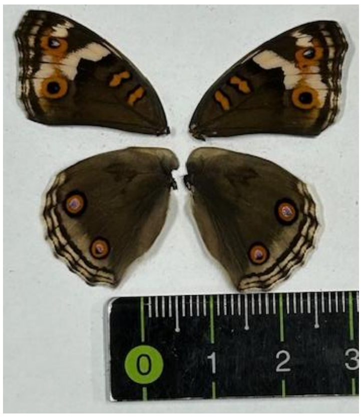

NO12

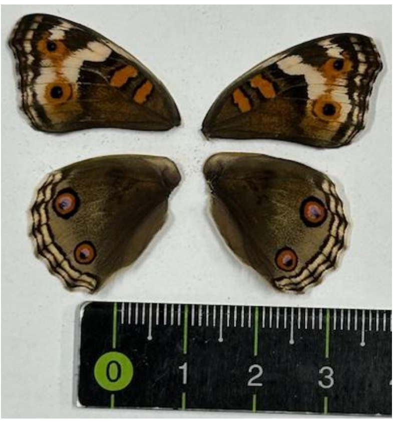

NO13

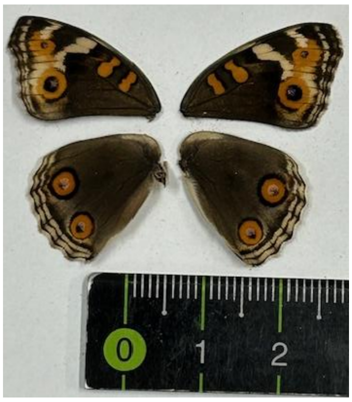

NO14

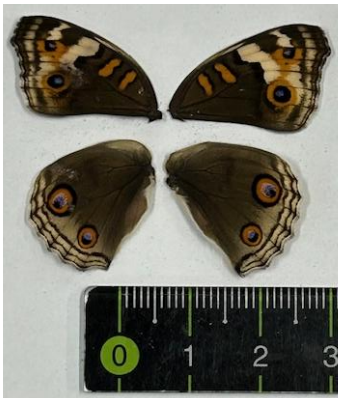

NO15

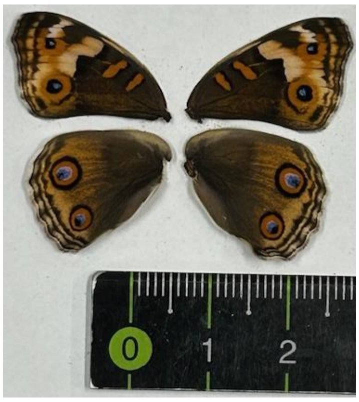

NO16

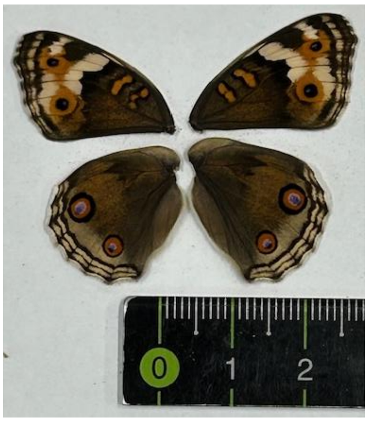

NO17

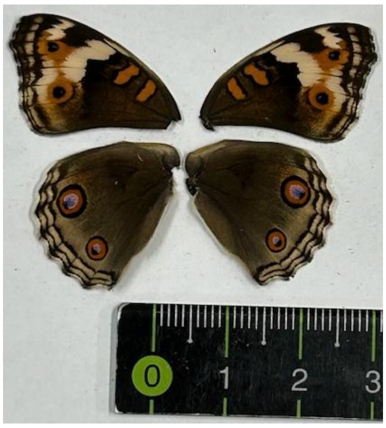

NO18

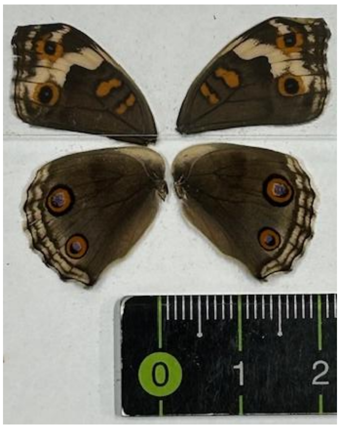

NO19

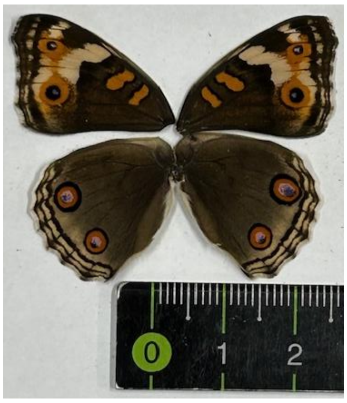

NO20

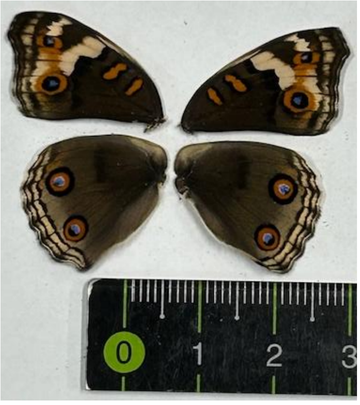

NO21

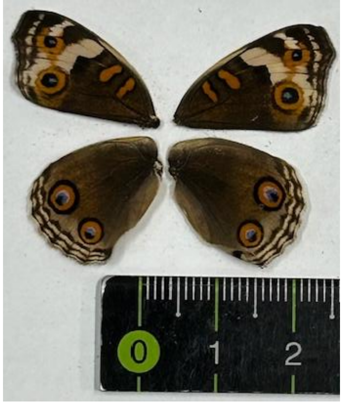

NO22

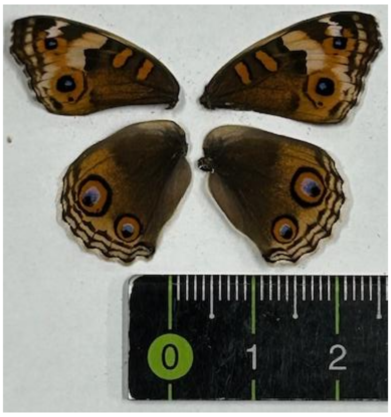

NO23

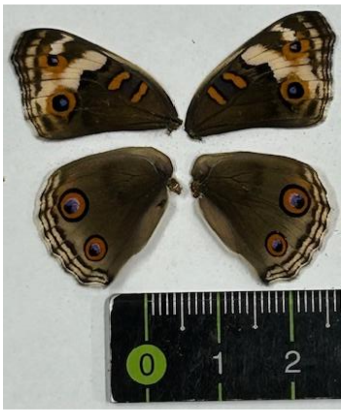

NO24

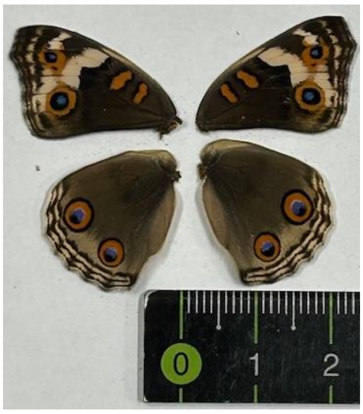

NO25

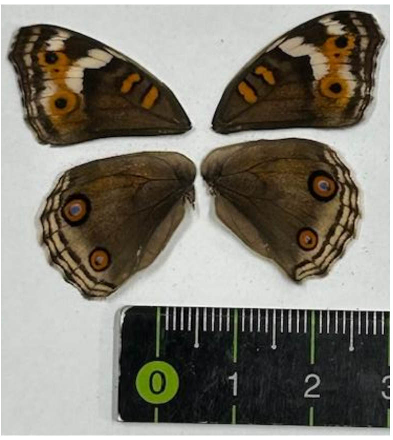

NO26

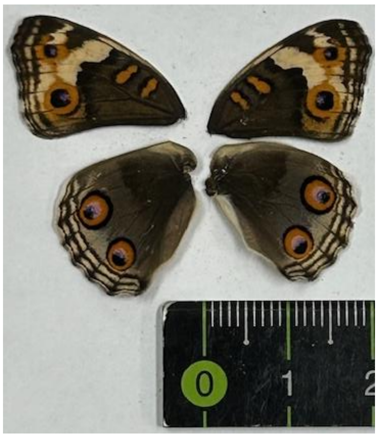

NO27

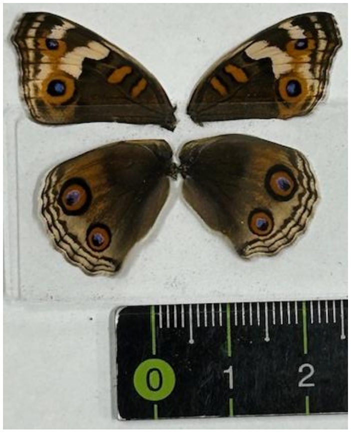

NO28

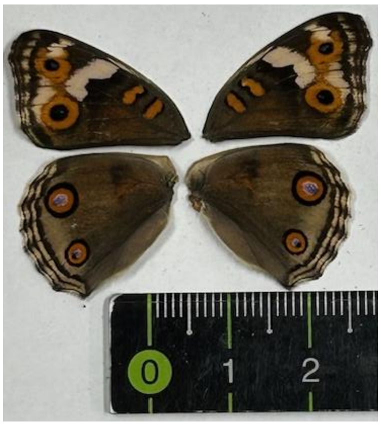

NO29

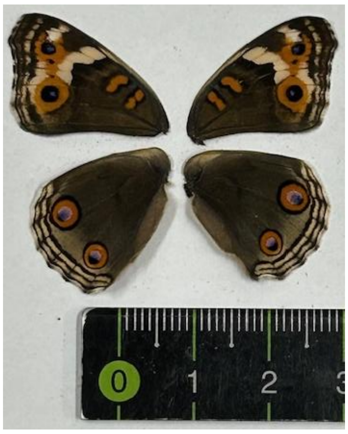

NO30

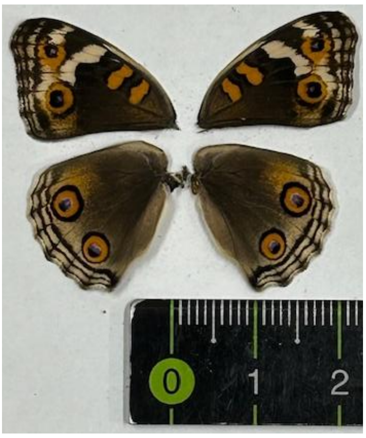

(d) No treatment, female, ventral side (n = 30)

NO1

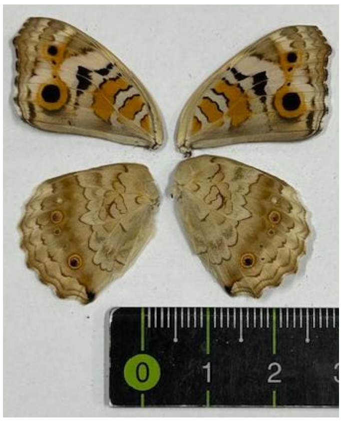

NO2

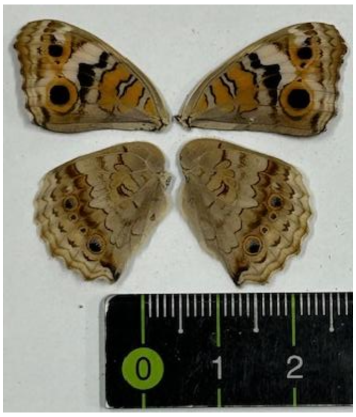

NO3

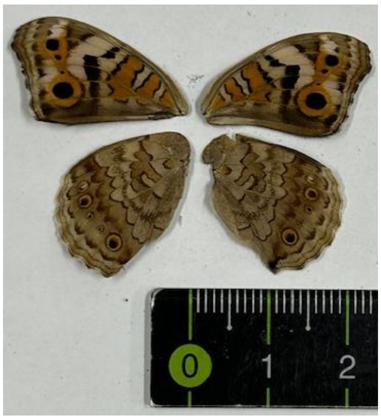

NO4

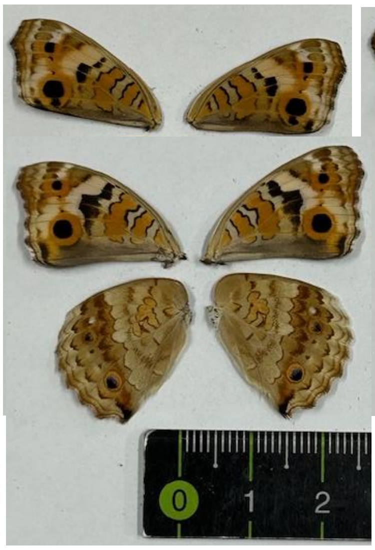

NO5

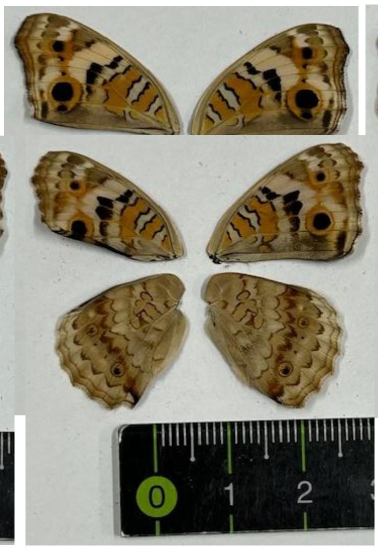

NO6

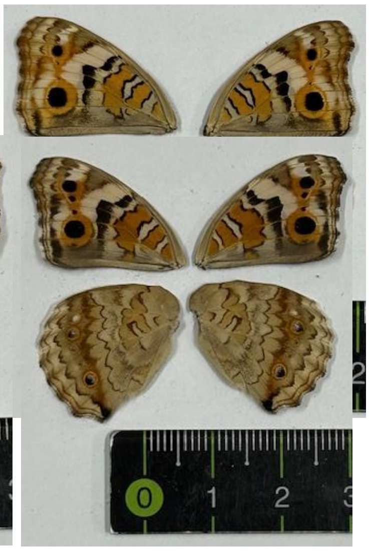

NO10

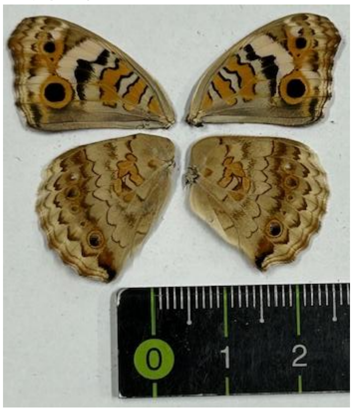

NO11

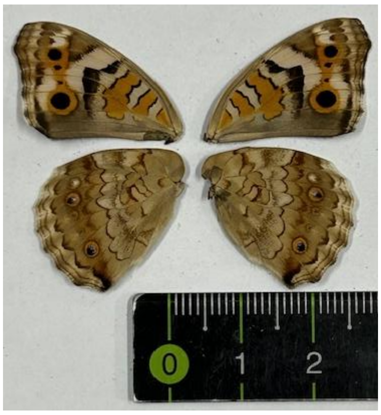

NO12

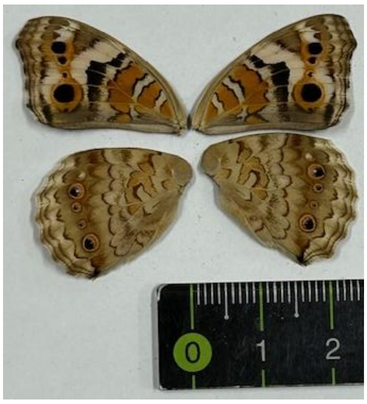

NO13

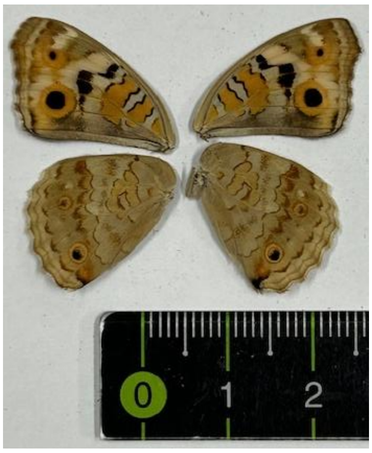

NO14

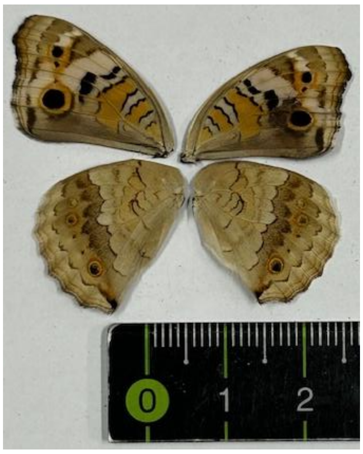

NO15

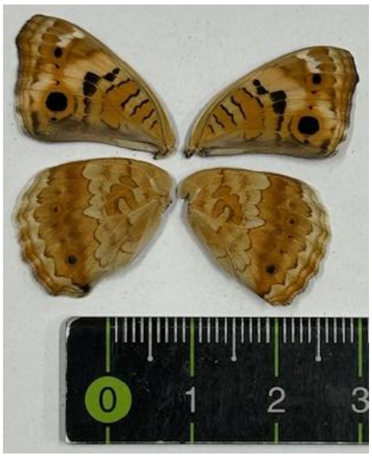

NO16

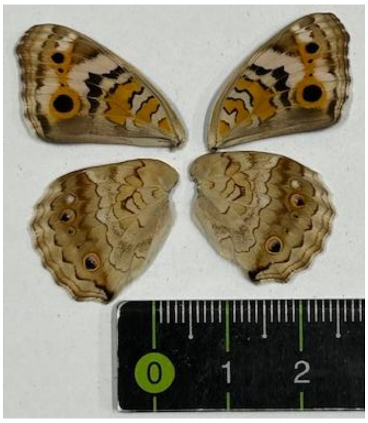

NO17

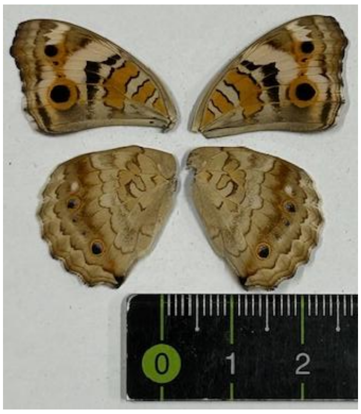

NO18

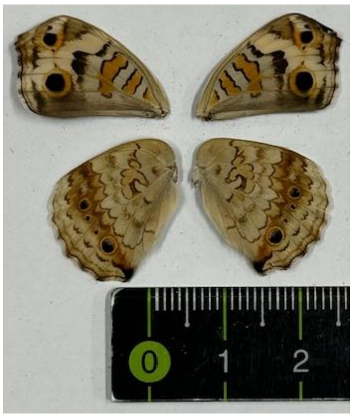

NO19

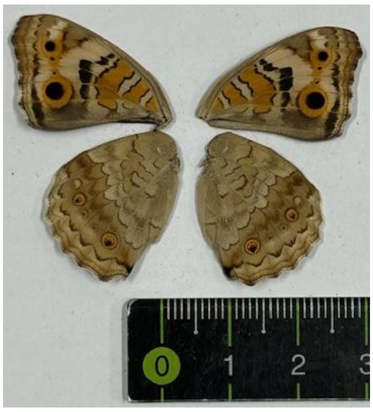

NO20

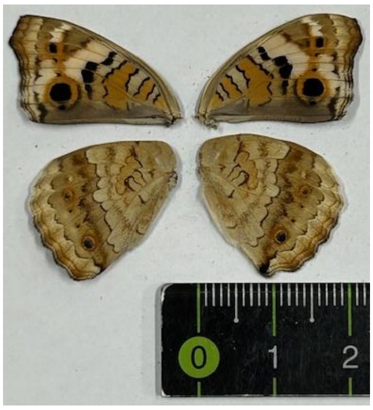

NO21

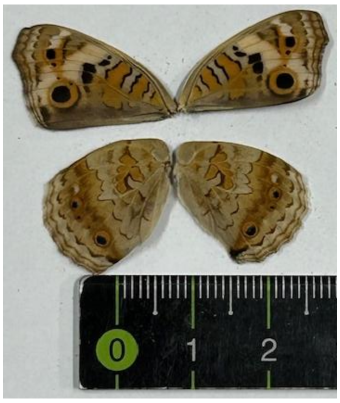

NO22

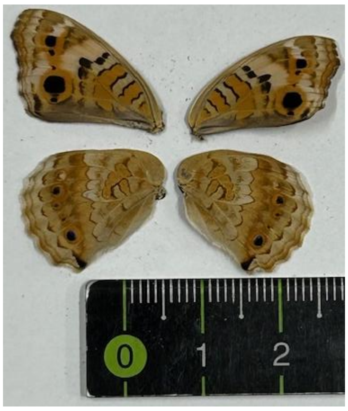

NO23

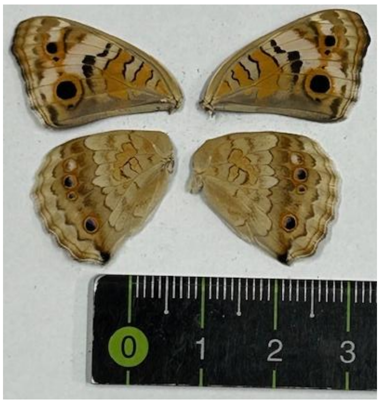

NO24

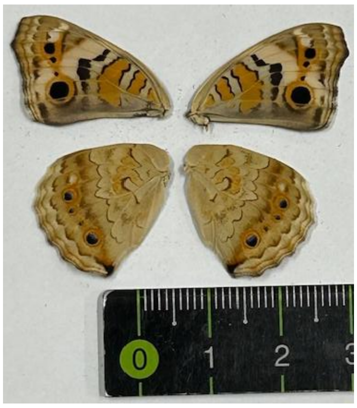

NO25

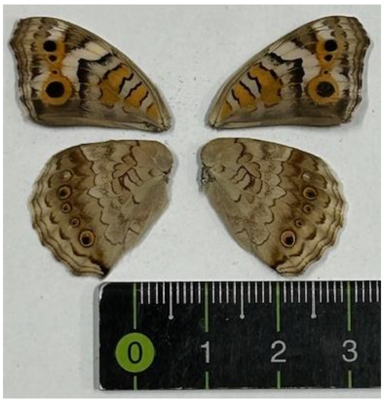

NO26

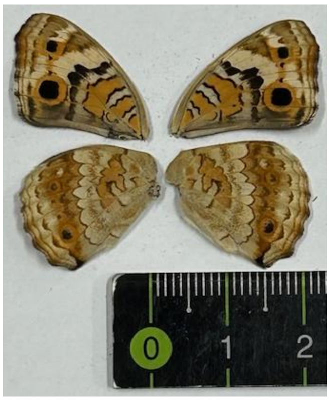

NO27

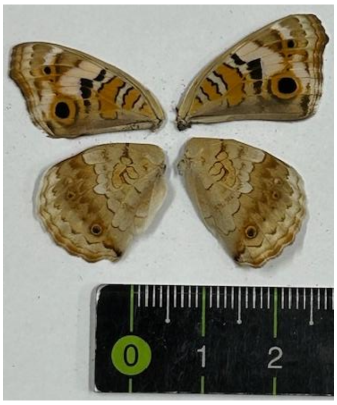

NO28

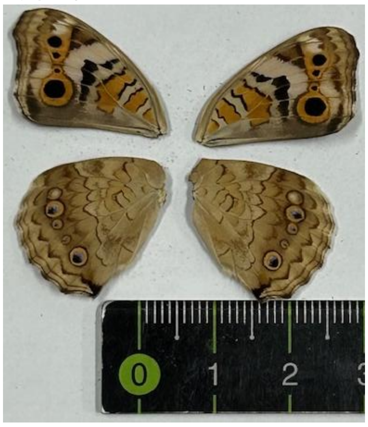

NO29

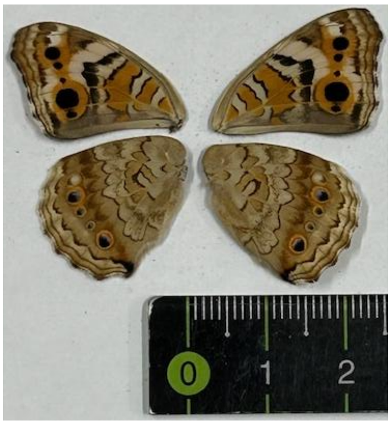

NO30

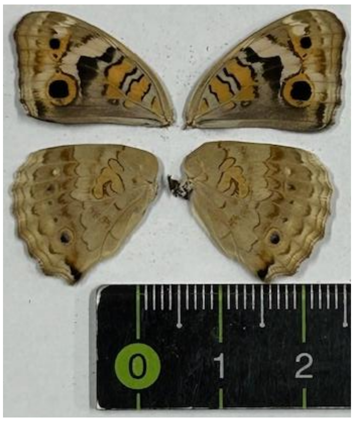

(e) VAS2870 injection (0.694 mM), male, dorsal side (n = 13)

NO1

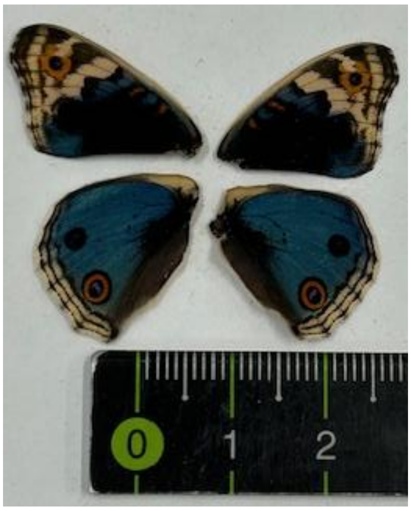

NO2

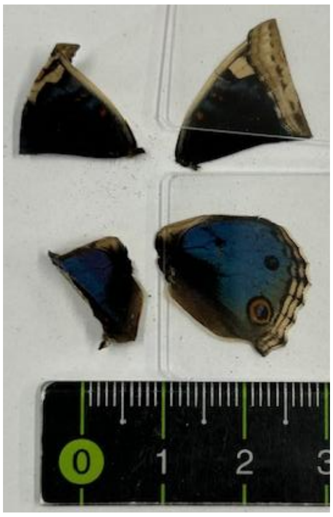

NO3

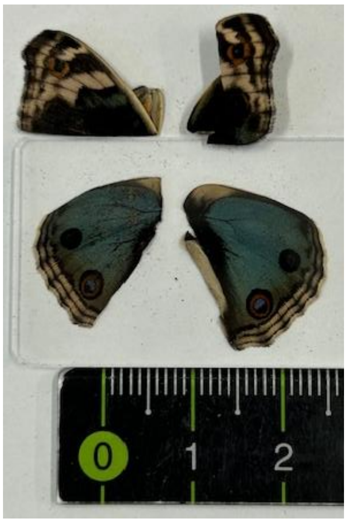

NO4

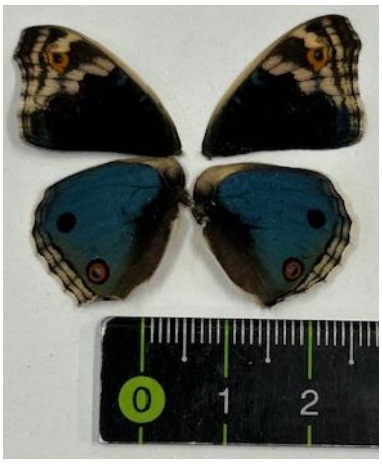

NO5

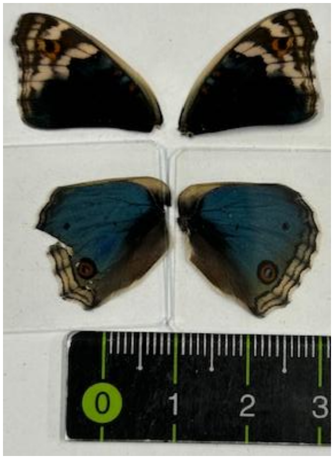

NO6

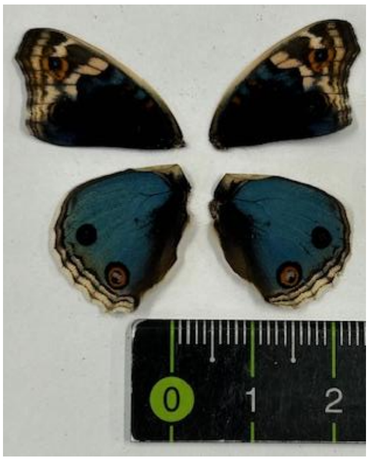

NO7

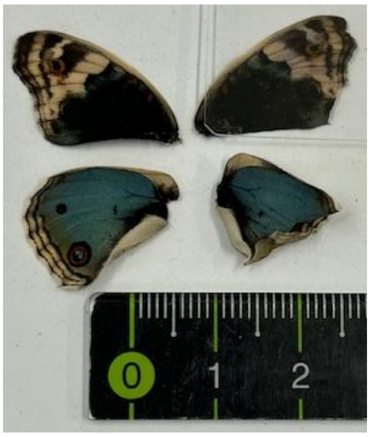

NO8

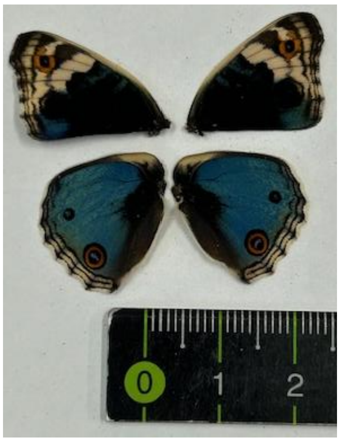

NO9

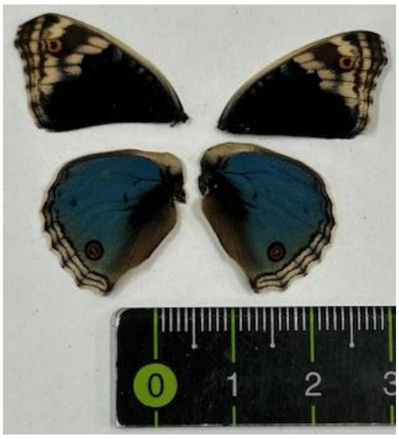

NO10

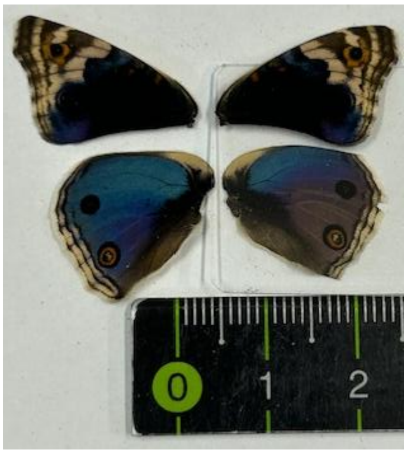

NO11

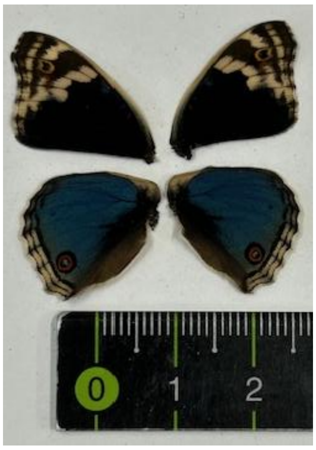

NO12

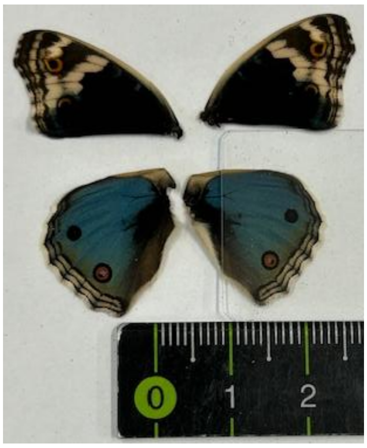

NO13

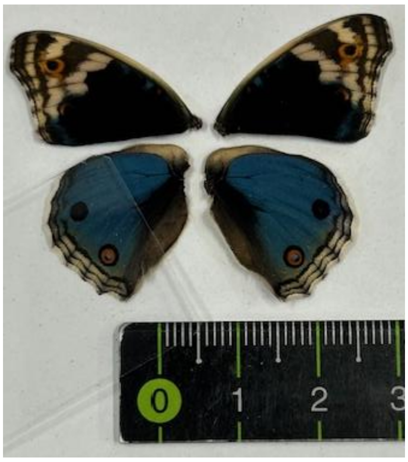

(f) VAS2870 injection (0.694 mM), male, ventral side (n = 13)

NO1

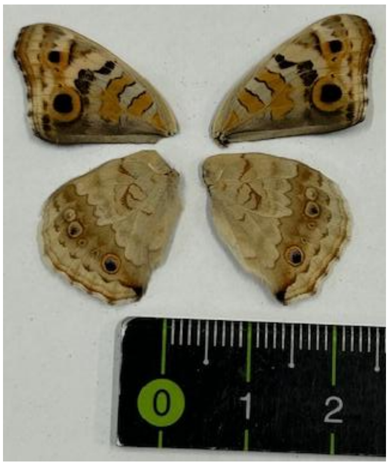

NO2

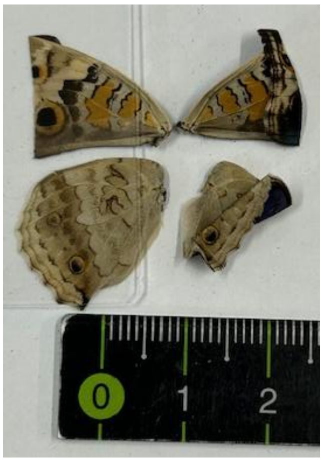

NO3

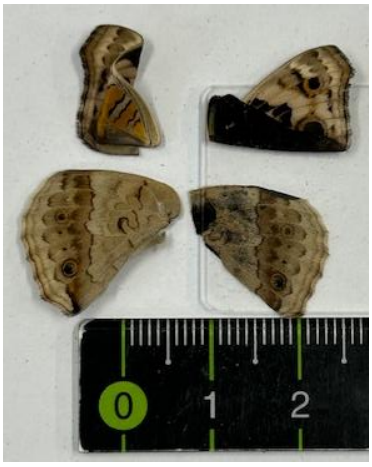

NO4

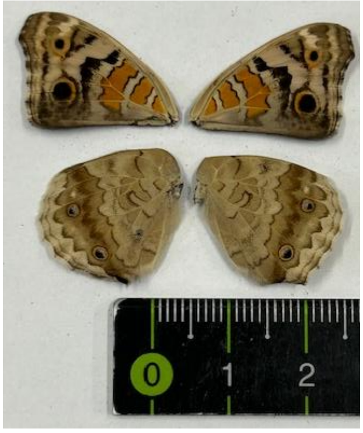

NO5

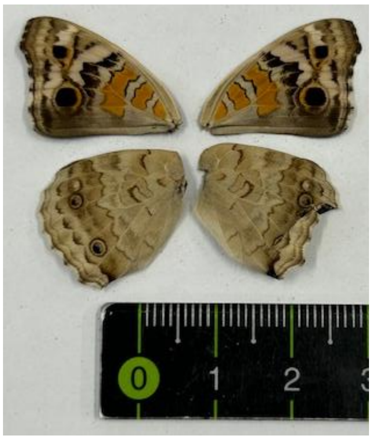

NO6

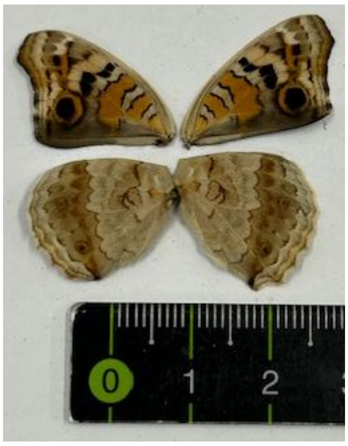

NO7

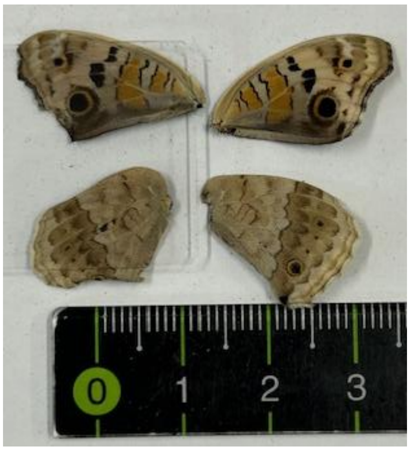

NO8

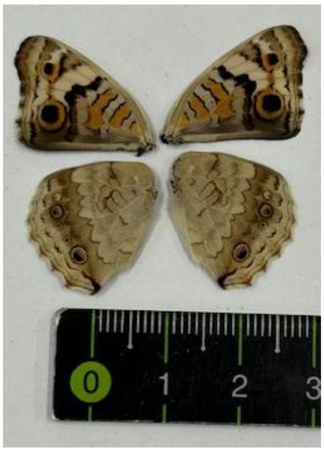

NO9

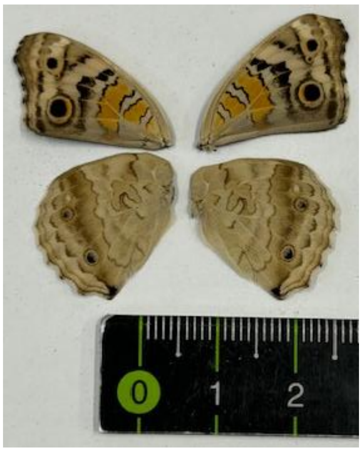

NO10

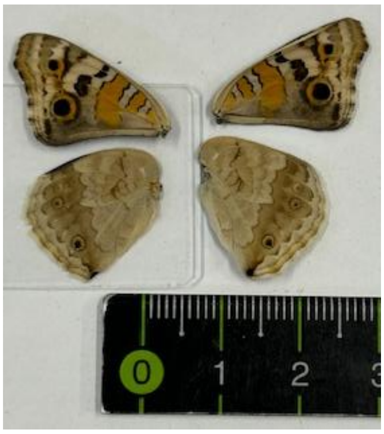

NO11

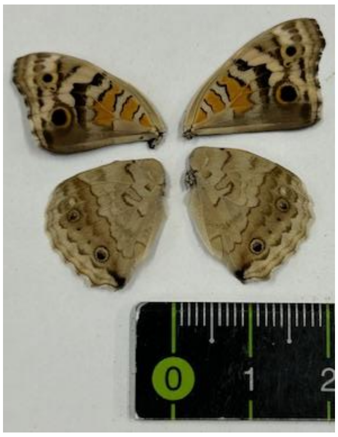

NO12

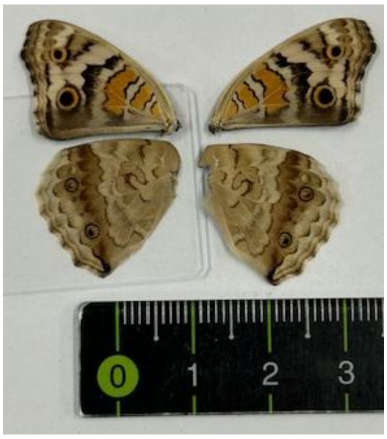

NO13

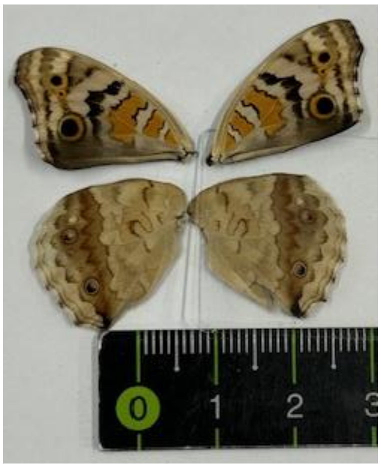

(g) VAS2870 injection (0.694 mM), female, dorsal side (n = 18)

NO1

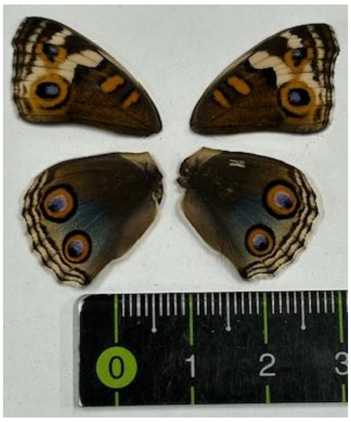

NO2

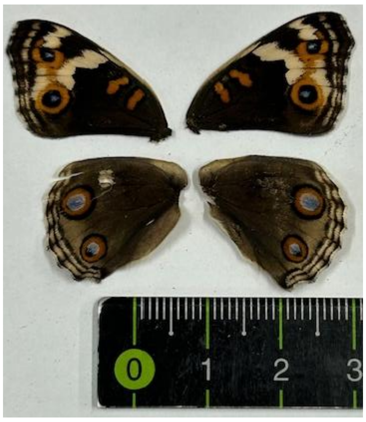

NO3

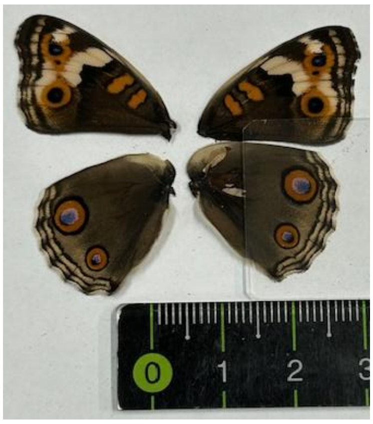

NO4

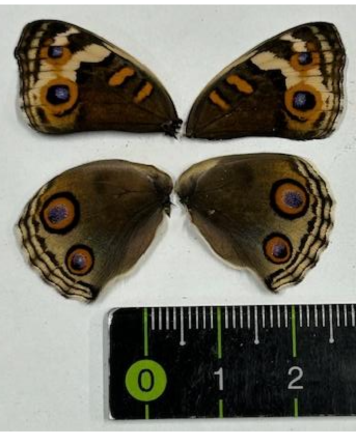

NO5

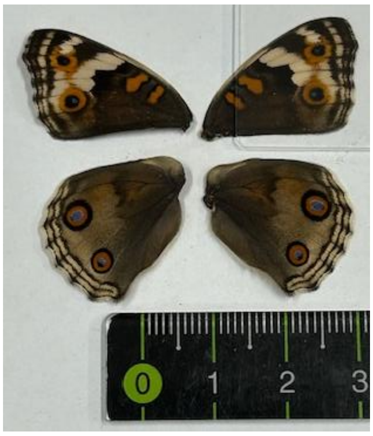

NO6

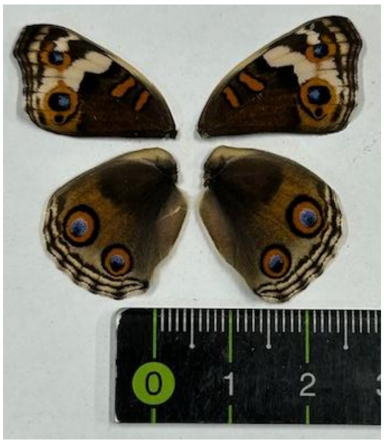

NO7

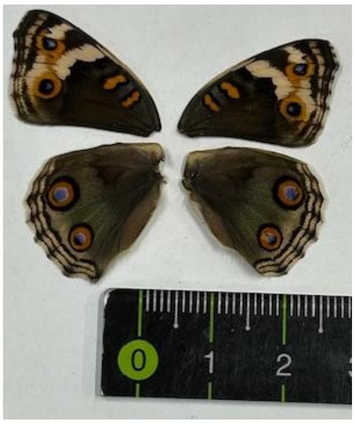

NO8

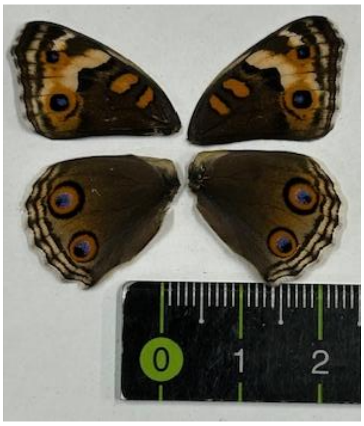

NO9

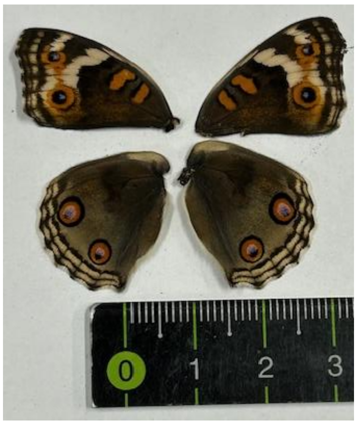

NO10

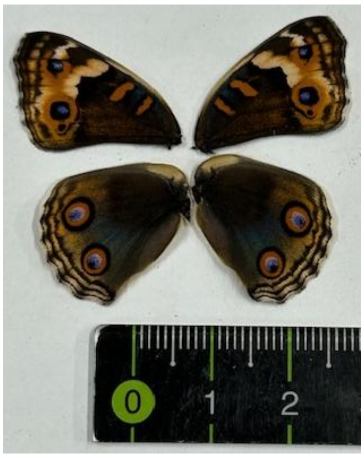

NO11

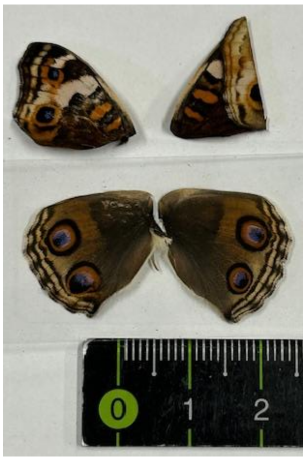

NO12

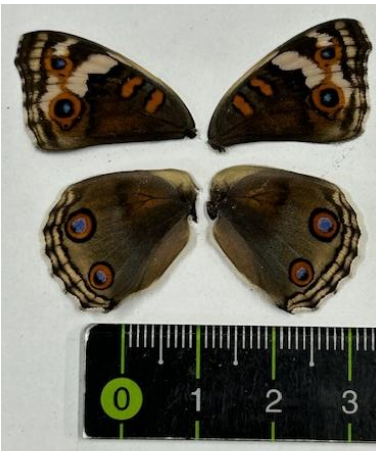

NO13

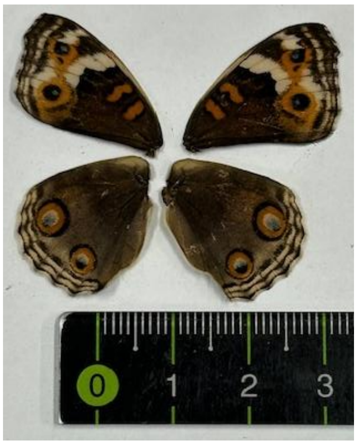

NO14

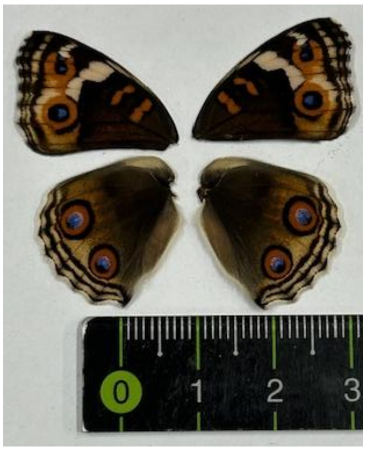

NO15

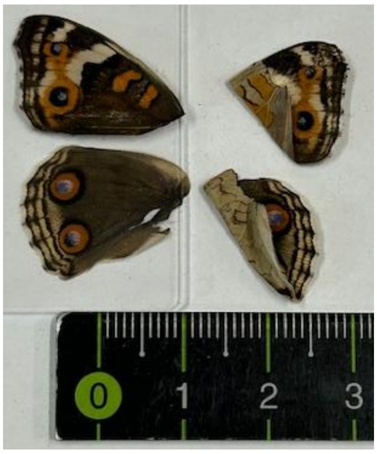

NO16

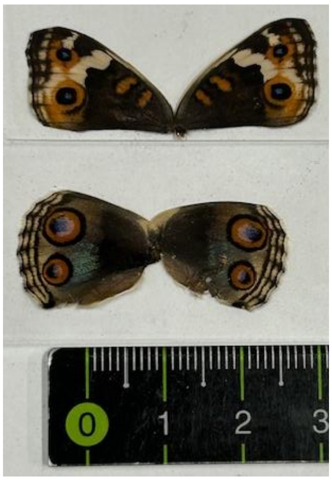

NO17

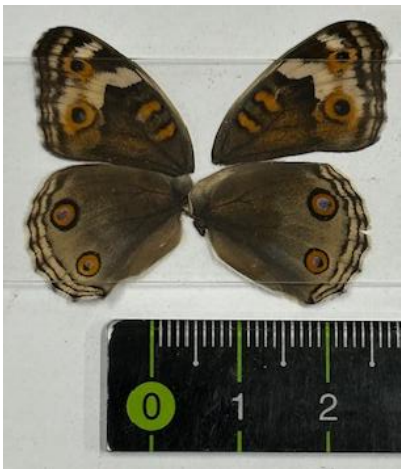

NO18

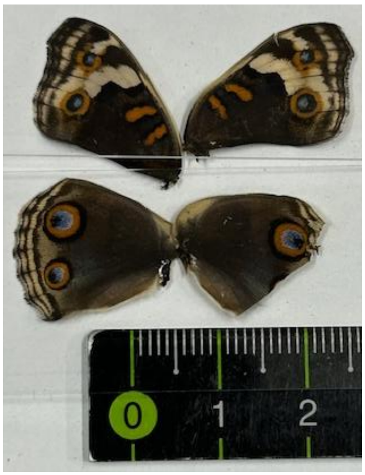

(h) VAS2870 injection (0.694 mM), female, ventral side ($n = 18$)

NO1

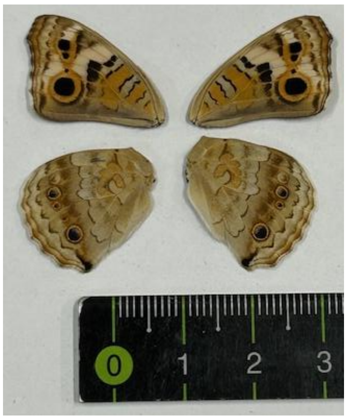

NO2

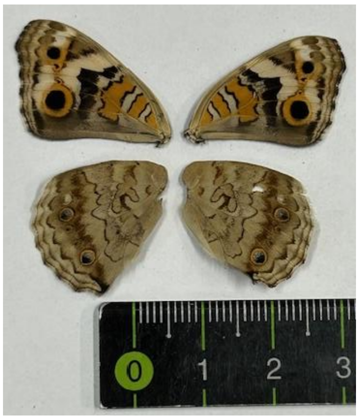

NO3

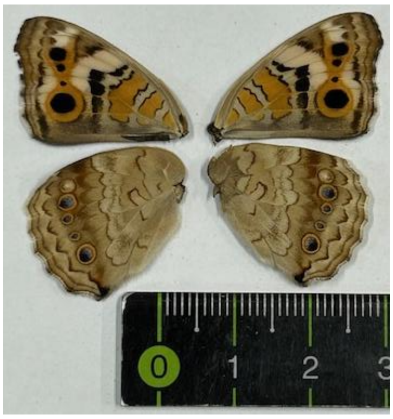

NO4

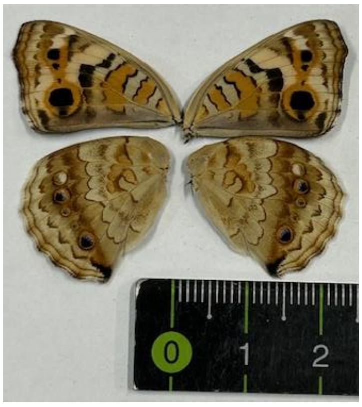

NO5

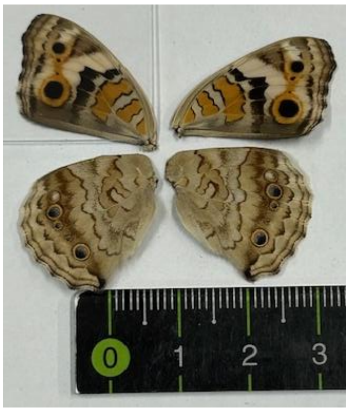

NO6

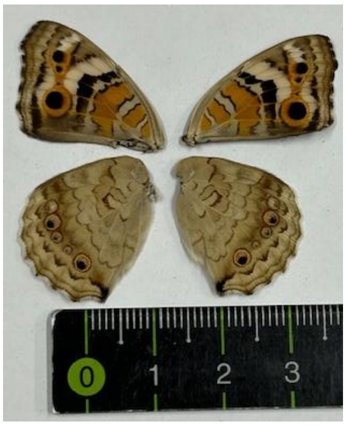

NO7

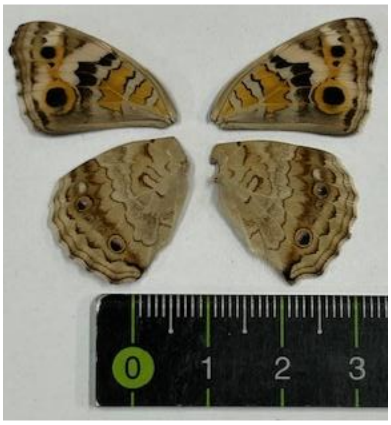

NO8

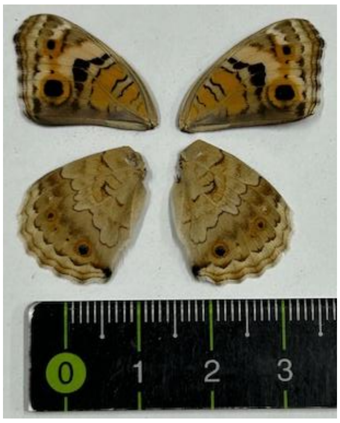

NO9

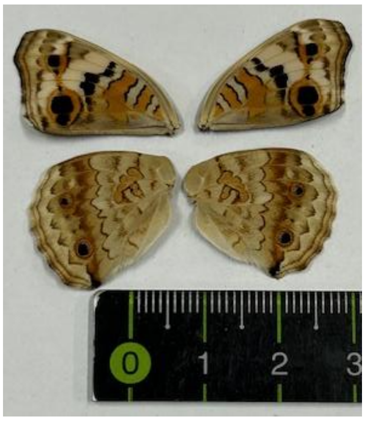

NO10

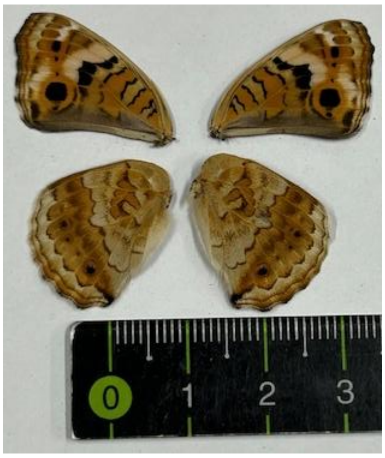

NO11

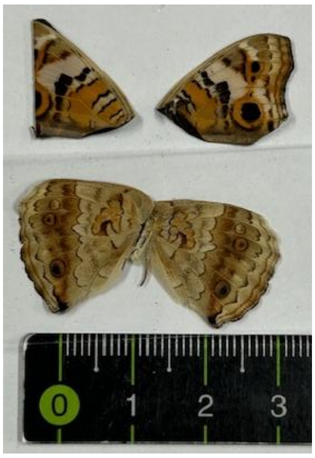

NO12

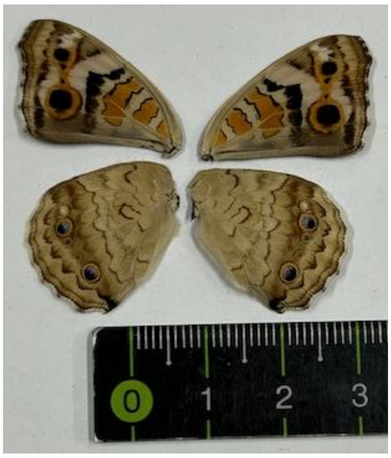

NO13

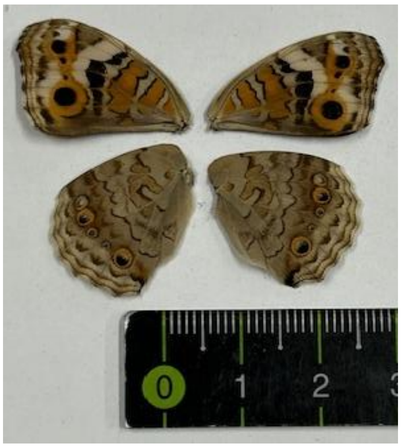

NO14

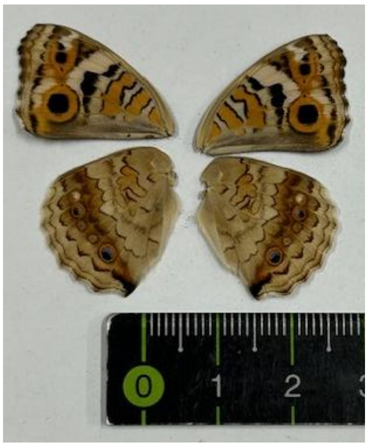

NO15

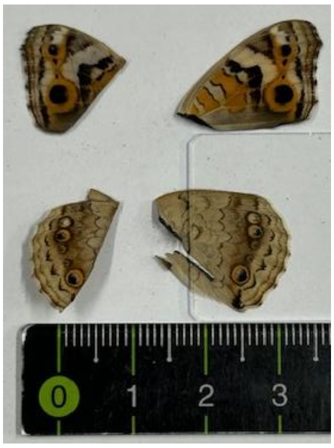

NO16

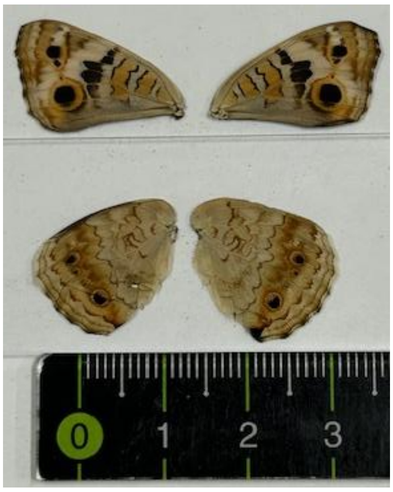

NO17

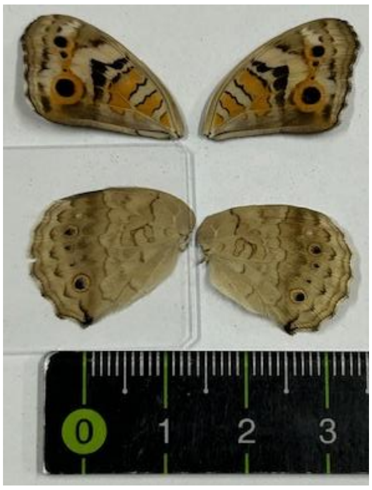

NO18

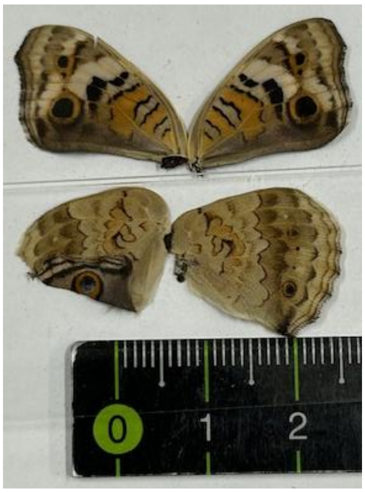

(i) VAS2870 injection (6.94 mM), male, dorsal side (n = 15)

NO1

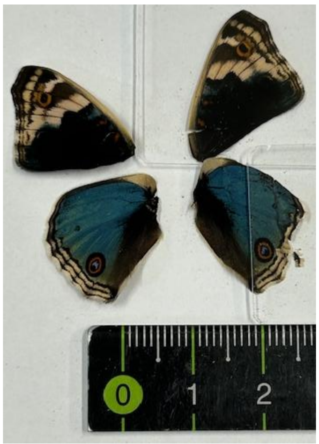

NO2

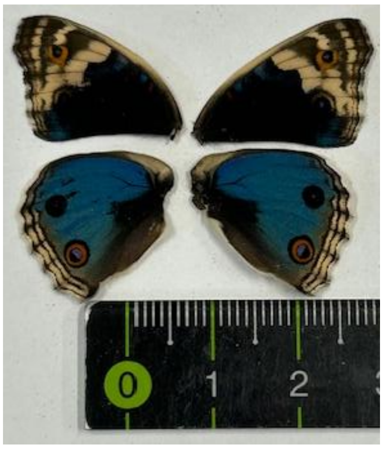

NO3

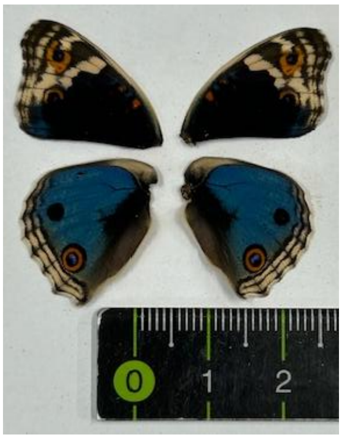

NO4

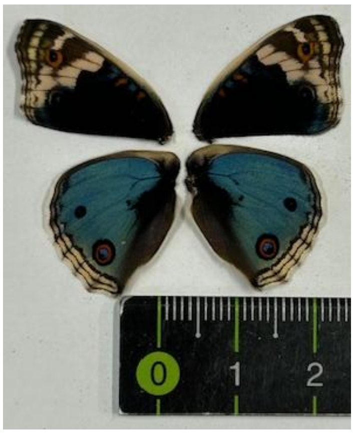

NO5

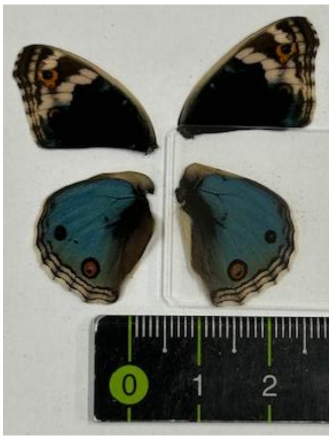

NO6

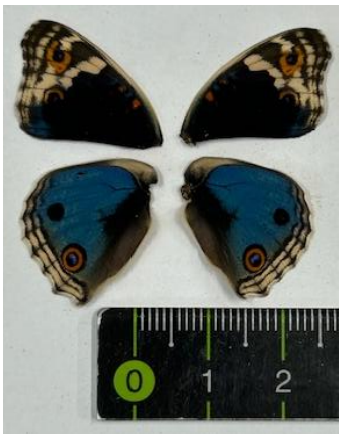

NO7

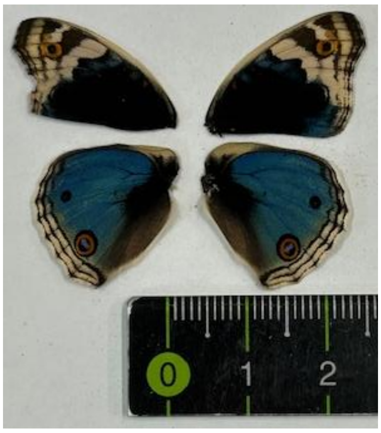

NO8

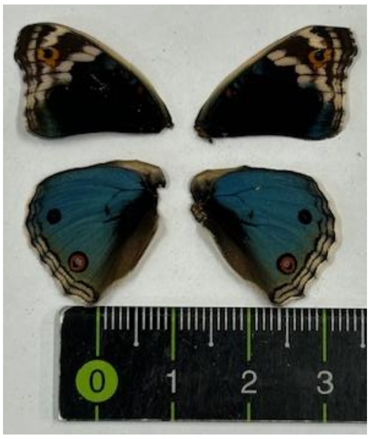

NO9

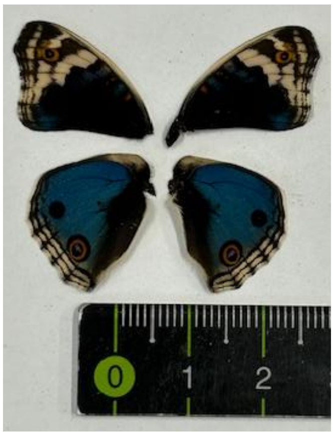

NO10

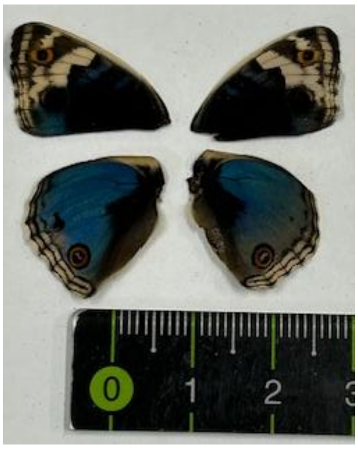

NO11

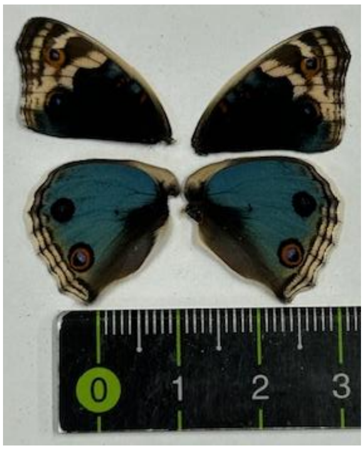

NO12

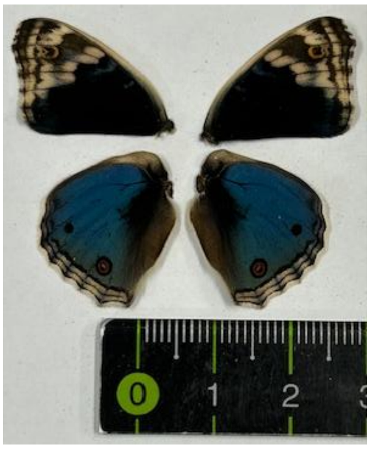

NO13

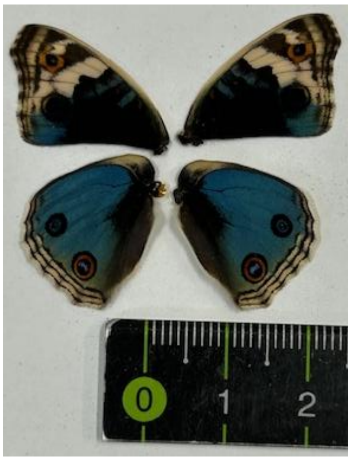

NO14

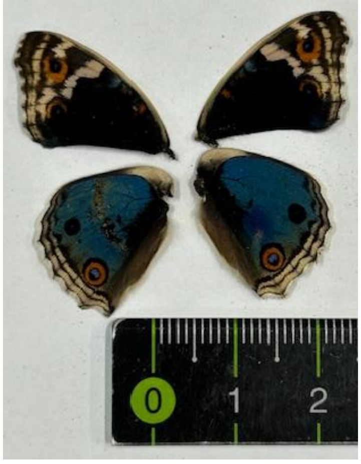

NO15

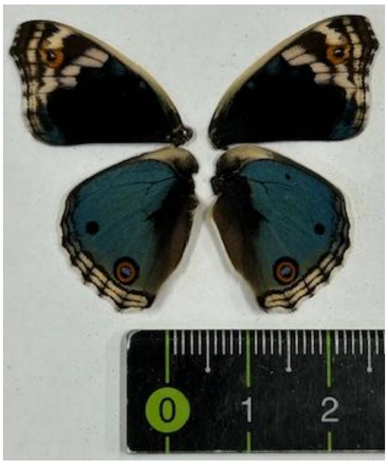

(j) VAS2870 injection (6.94 mM), male, ventral side (n = 15)

NO1

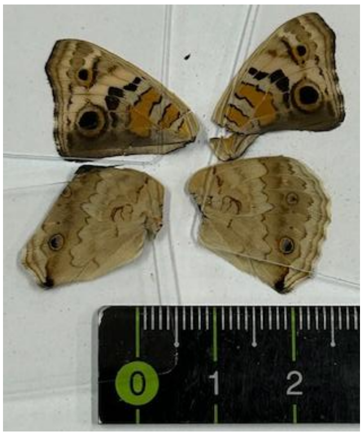

NO2

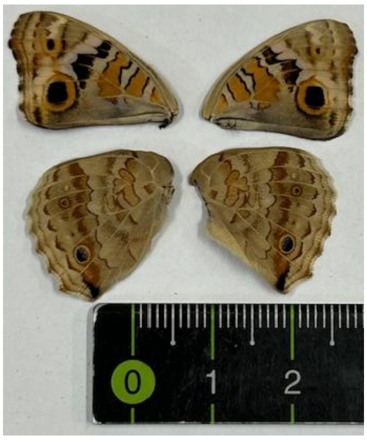

NO3

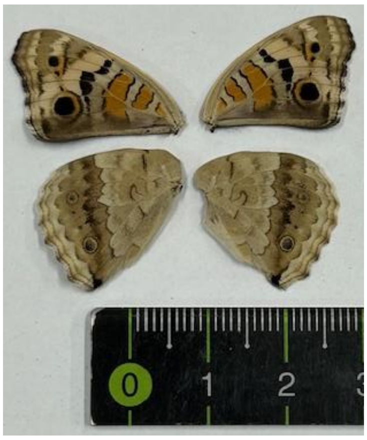

NO4

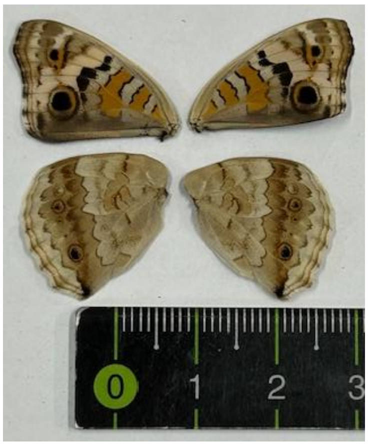

NO5

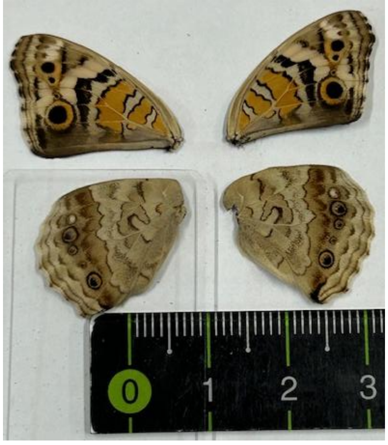

NO6

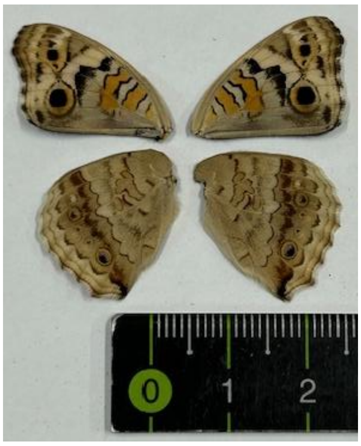

NO7

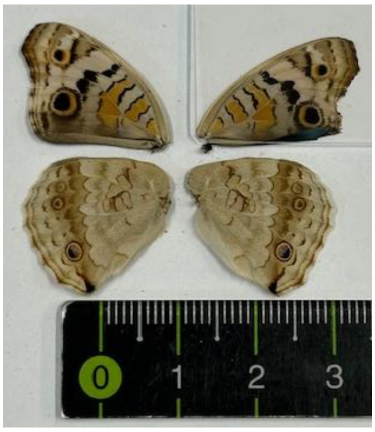

NO8

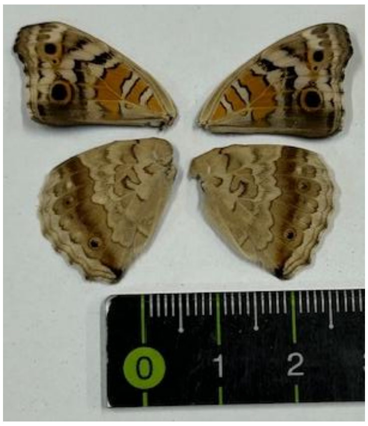

NO9

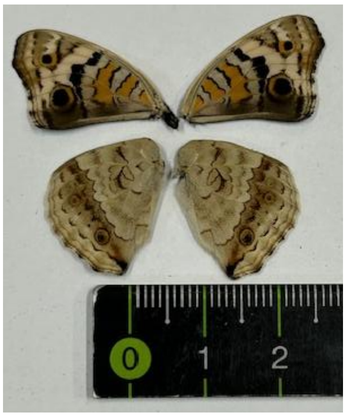

NO10

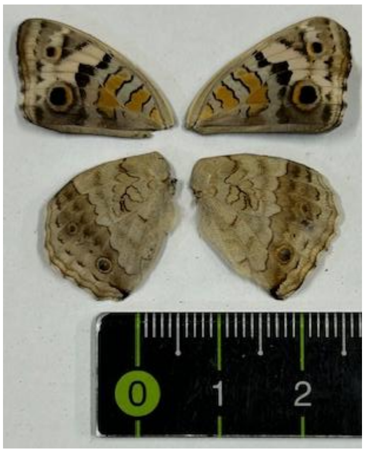

NO11

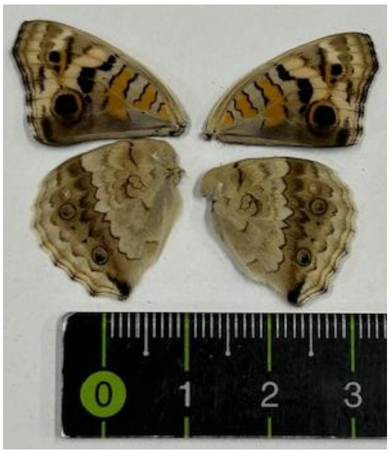

NO12

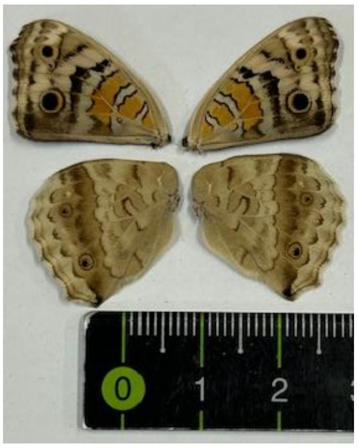

NO13

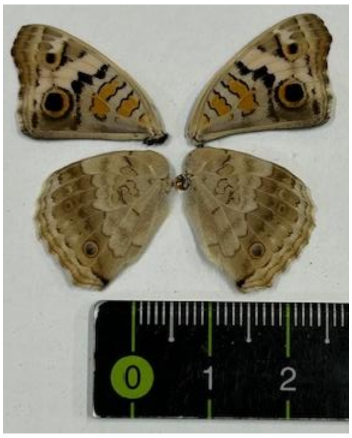

NO14

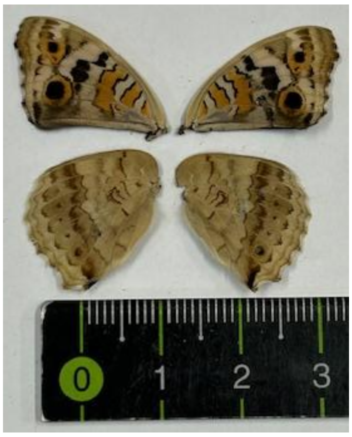

NO15

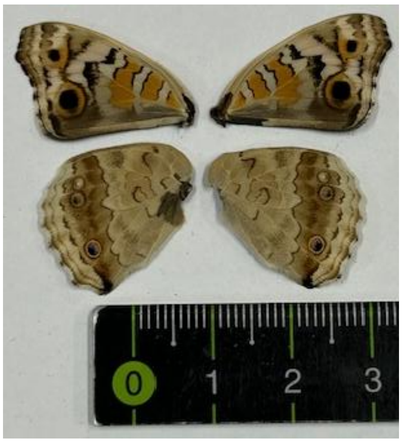

(k) VAS2870 injection (6.94 mM), female, dorsal side ($n = 15$)

NO1

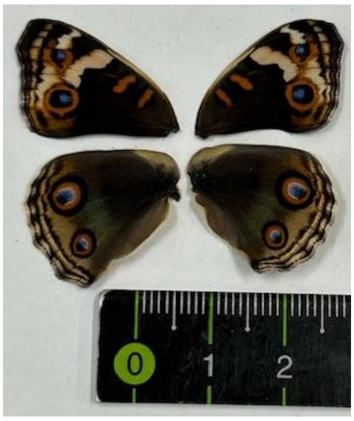

NO2

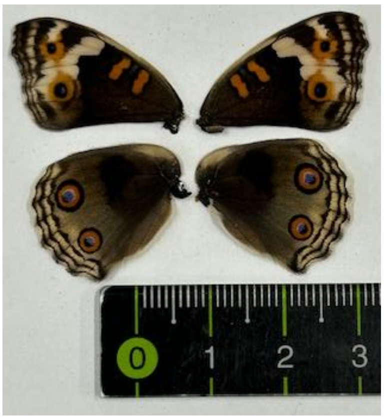

NO3

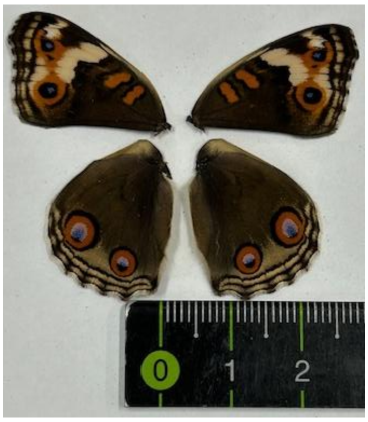

NO4

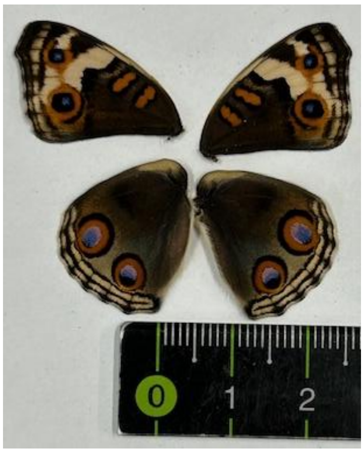

NO5

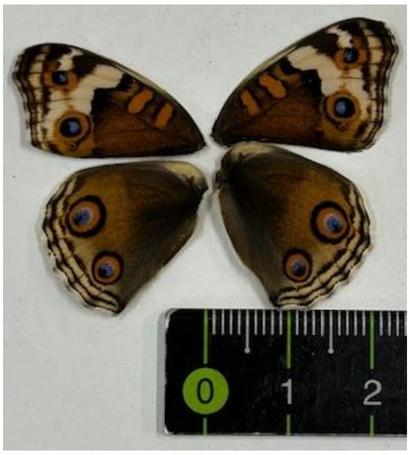

NO6

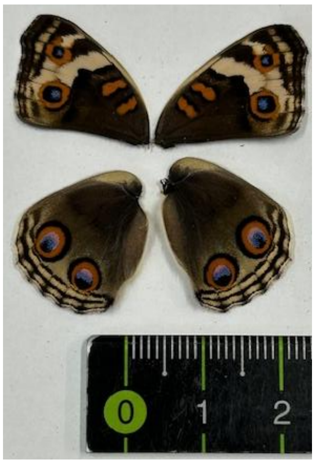

NO7

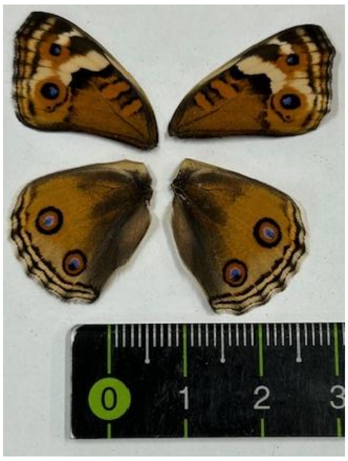

NO8

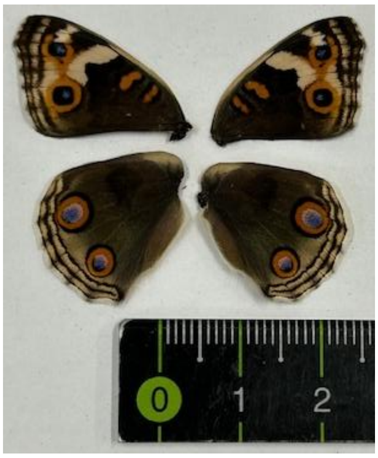

NO9

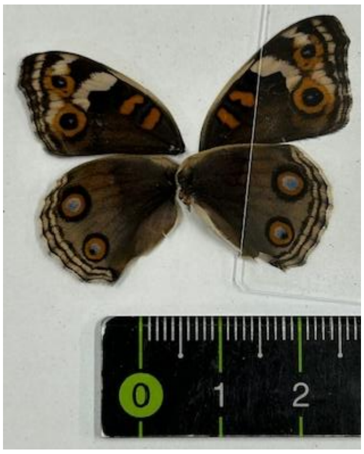

NO10

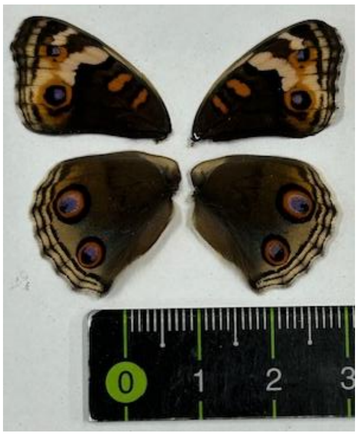

NO11

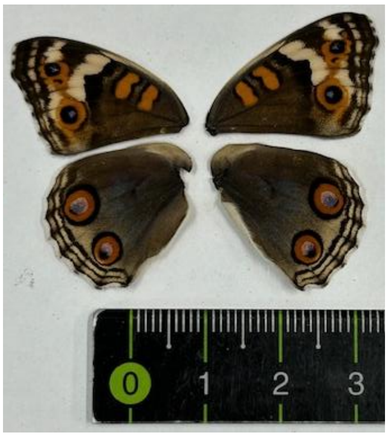

NO12

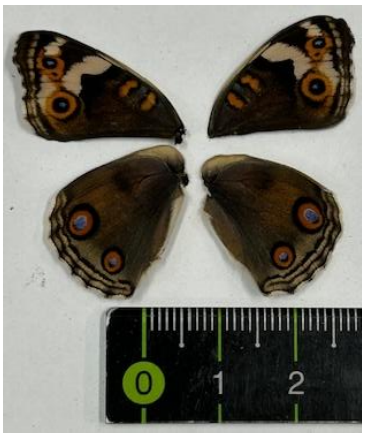

NO13

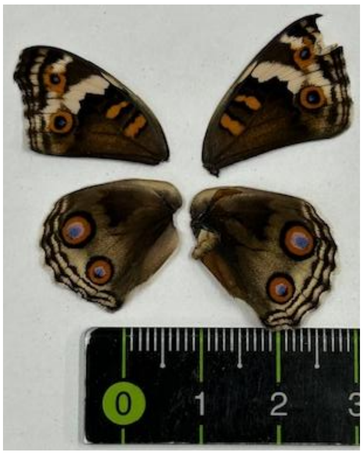

NO14

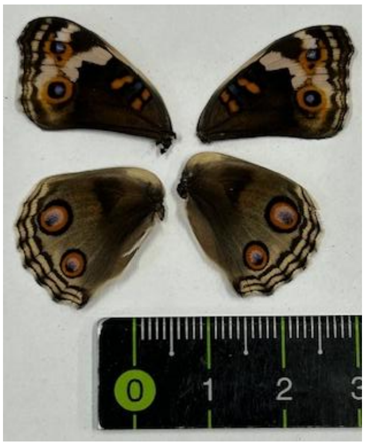

NO15

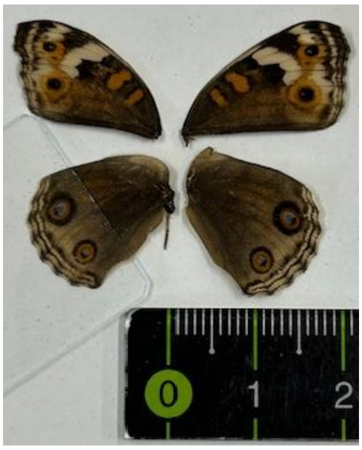

(l) VAS2870 injection (6.94 mM), female, ventral side (n = 15)

NO1

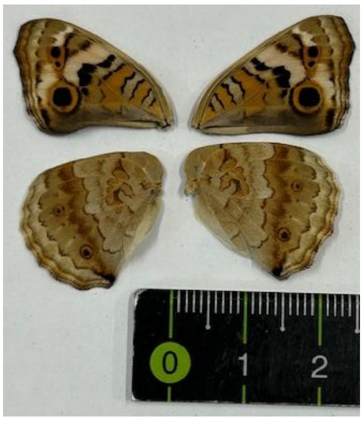

NO2

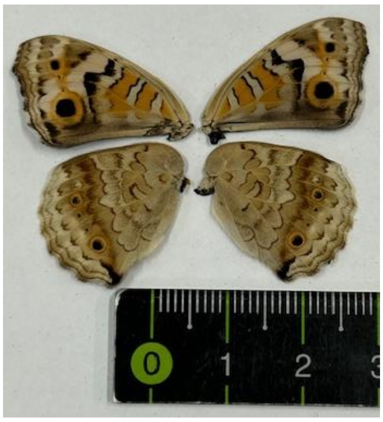

NO3

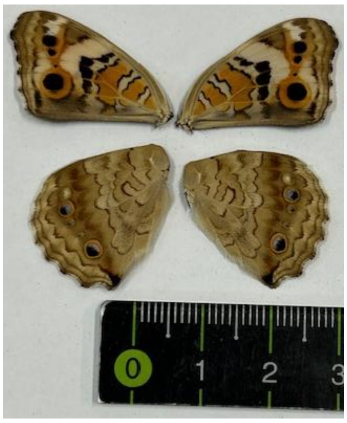

NO4

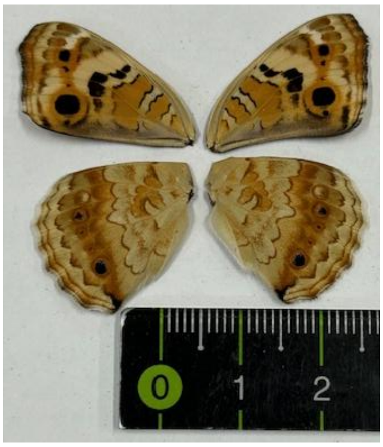

NO5

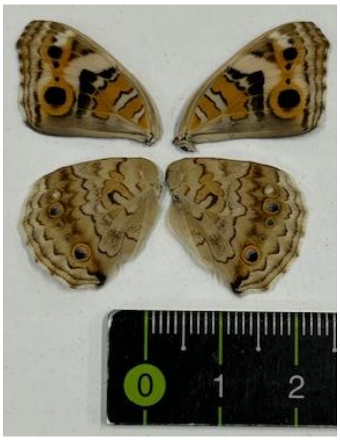

NO6

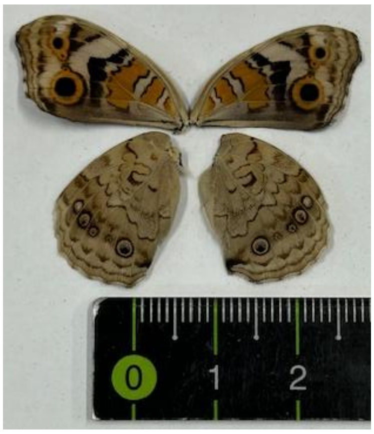

NO7

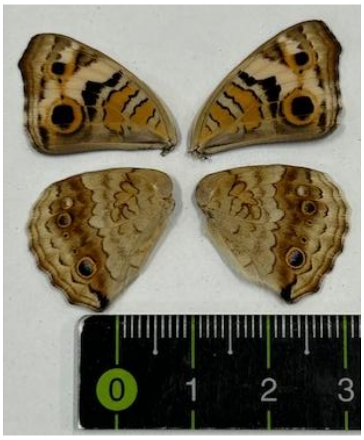

NO8

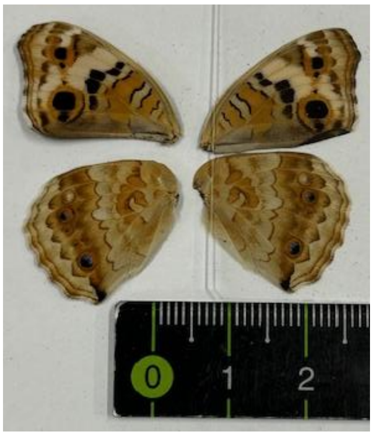

NO9

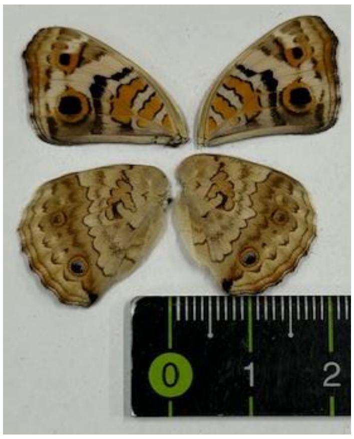

NO10

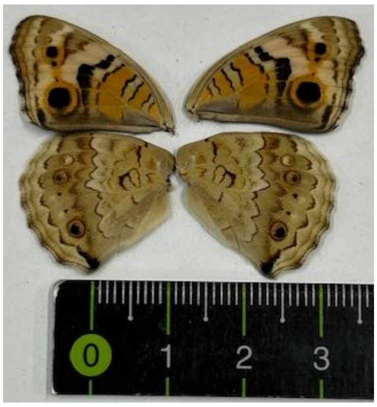

NO11

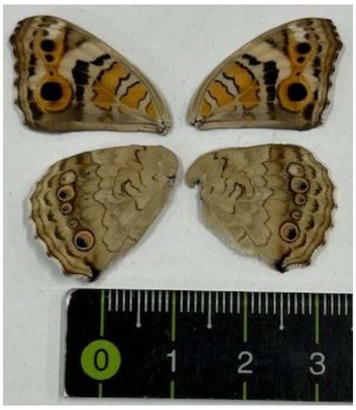

NO12

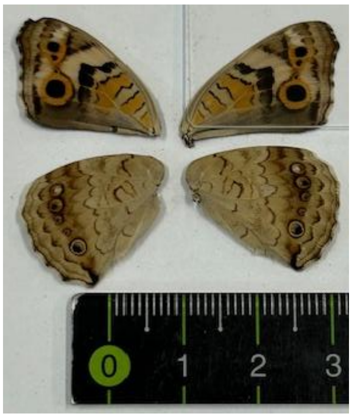

NO13

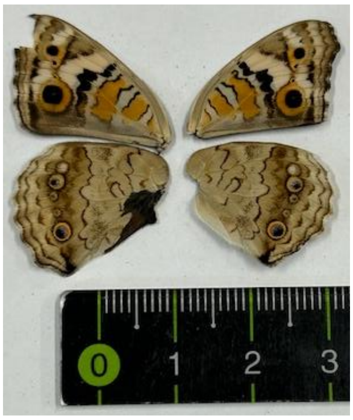

NO14

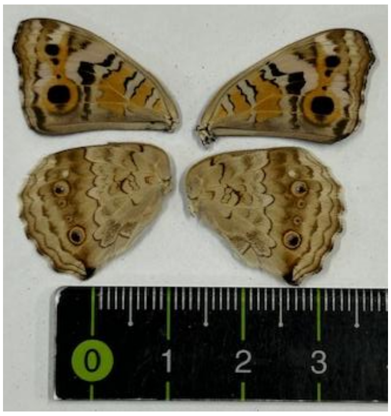

NO15

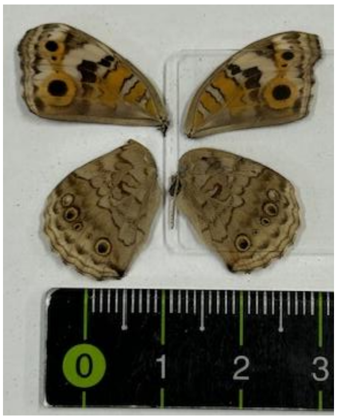

(m) VAS2870 injection (13.87 mM), male, dorsal side (n = 18)

NO1

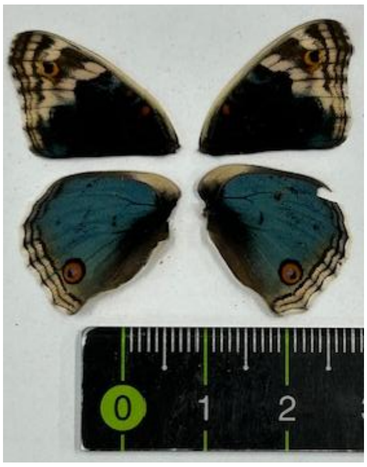

NO2

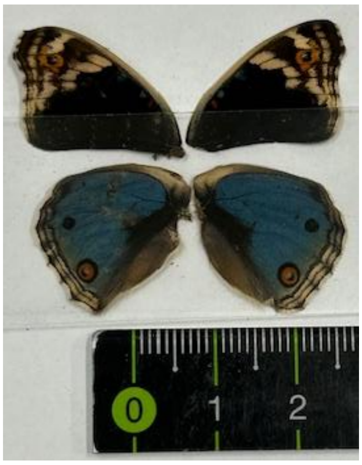

NO3

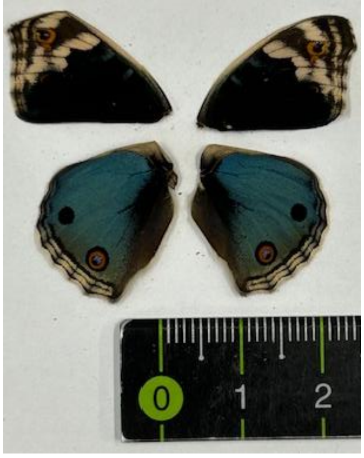

NO4

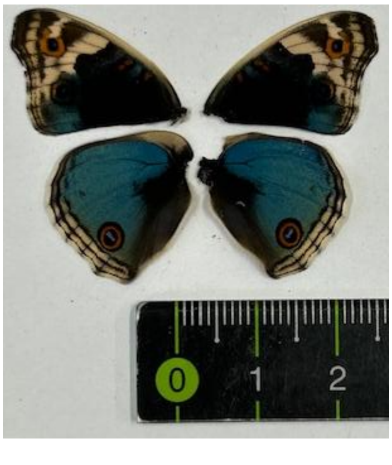

NO5

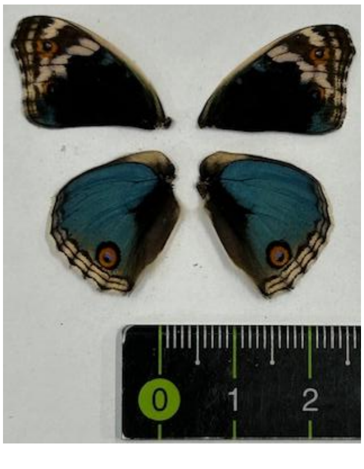

NO6

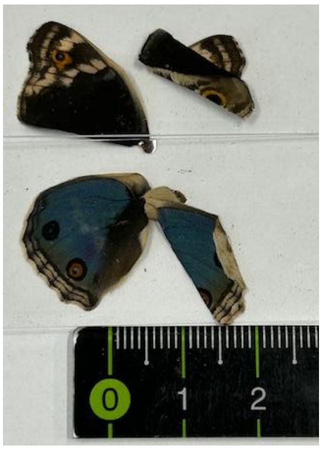

NO7

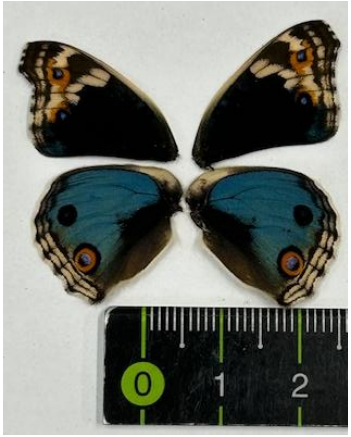

NO8

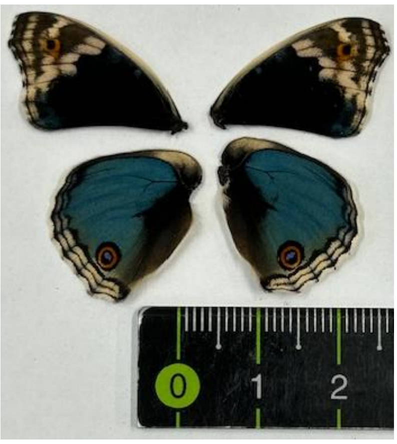

NO9

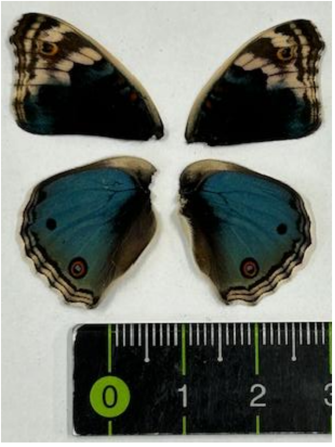

NO10

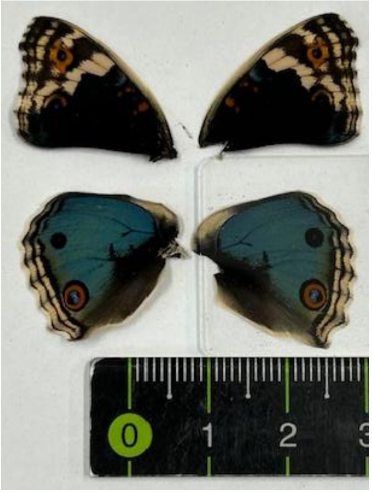

NO11

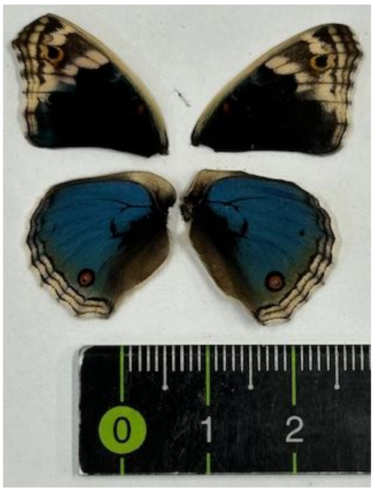

NO12

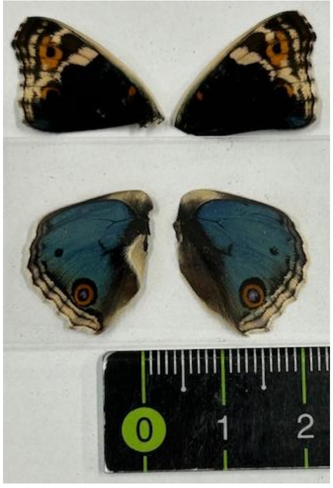

NO13

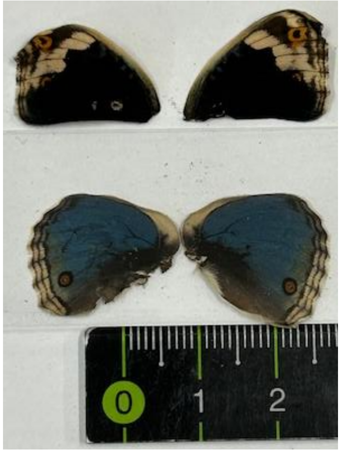

NO14

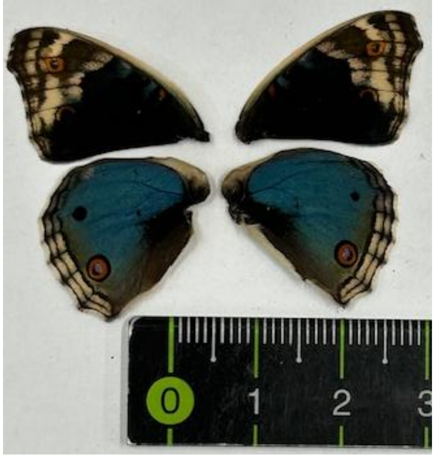

NO15

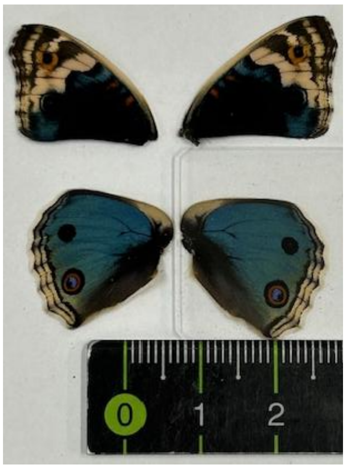

NO16

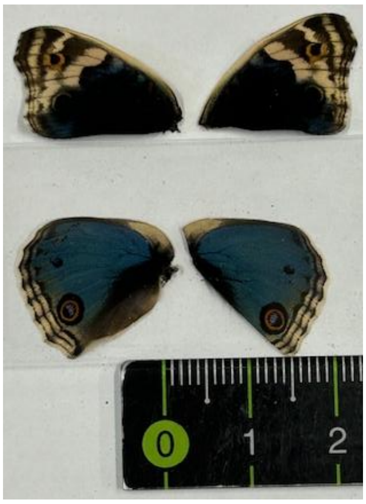

NO17

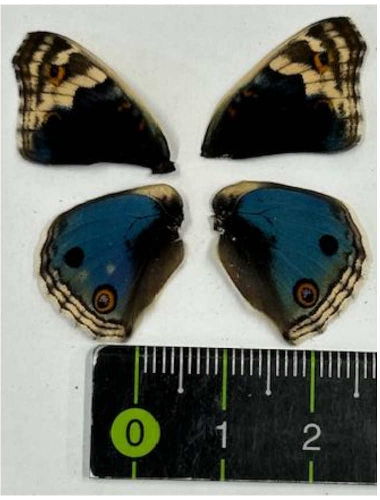

NO18

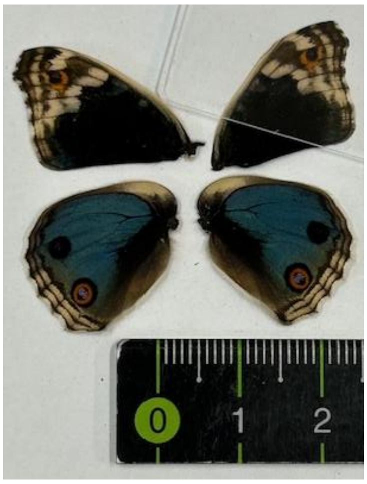

(n) VAS2870 injection (13.87 mM), male, ventral side (*n* =18)

NO1

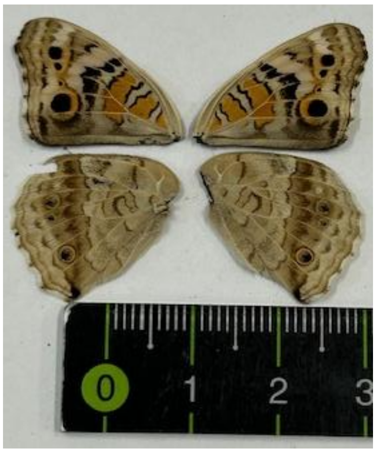

NO2

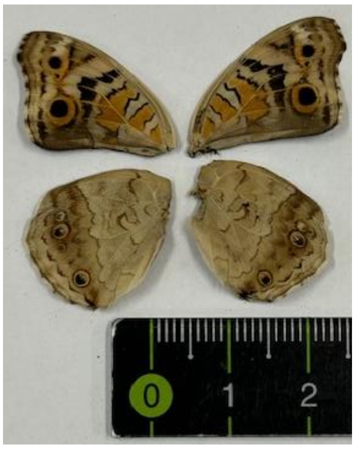

NO3

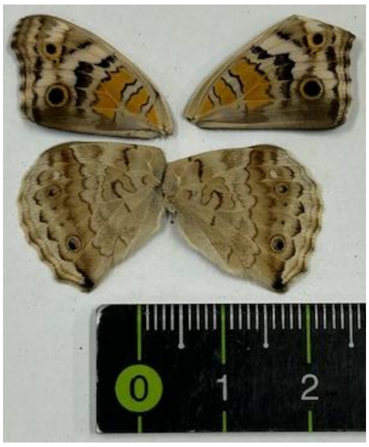

NO4

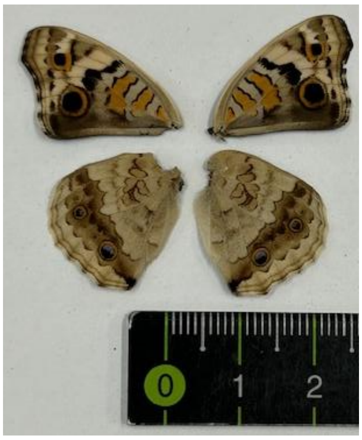

NO5

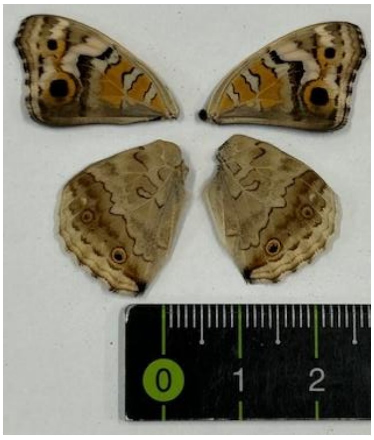

NO6

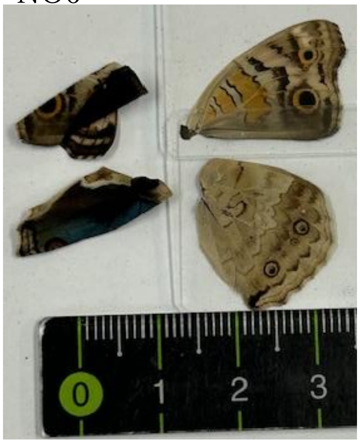

NO7

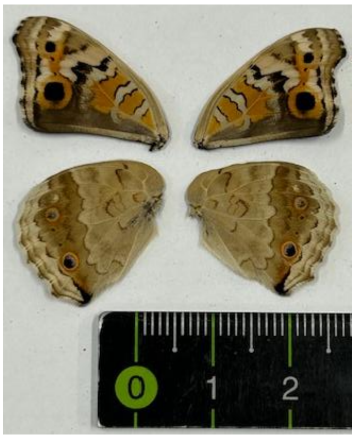

NO8

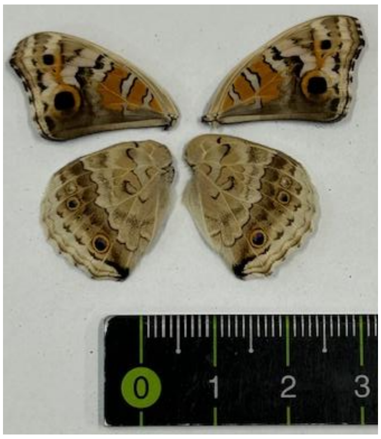

NO9

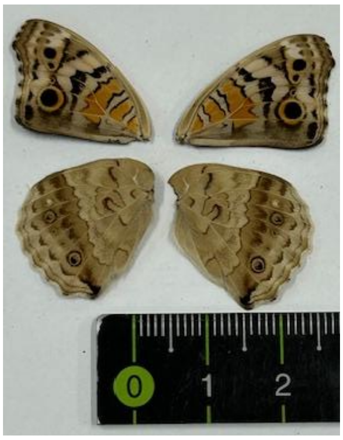

NO10

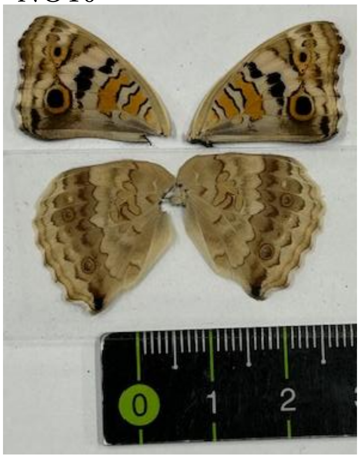

NO11

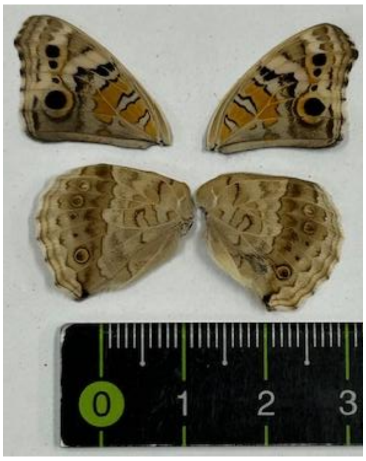

NO12

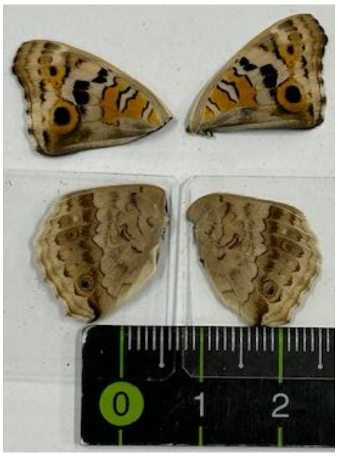

NO13

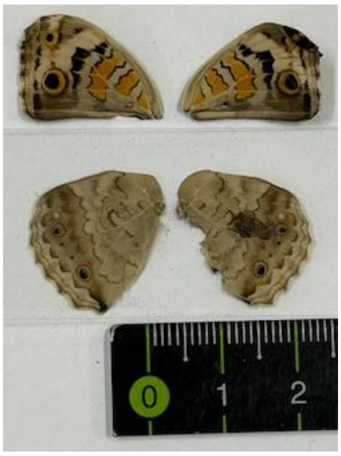

NO14

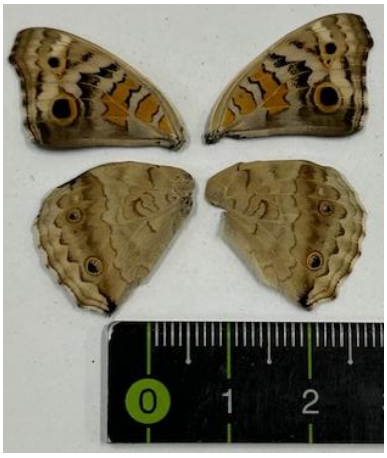

NO15

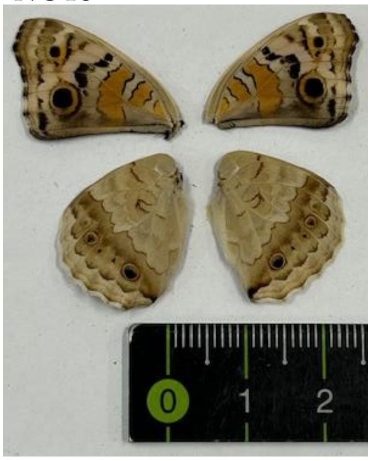

NO16

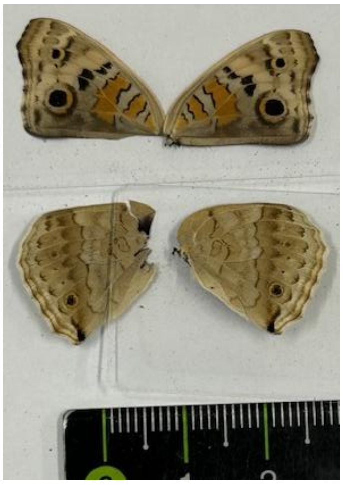

NO17

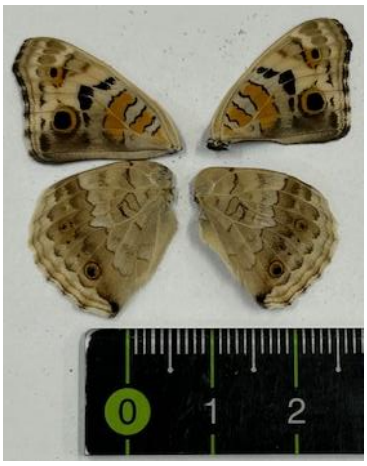

NO18

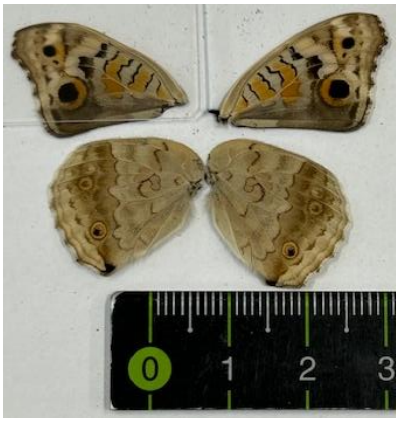

(o) VAS2870 injection (13.87 mM), female, dorsal side (n =13)

NO1

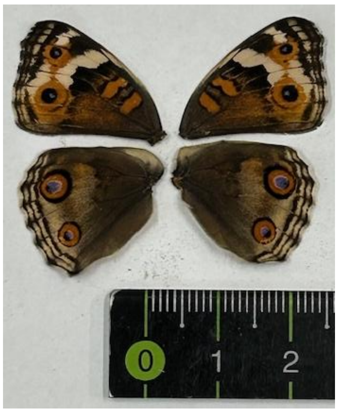

NO2

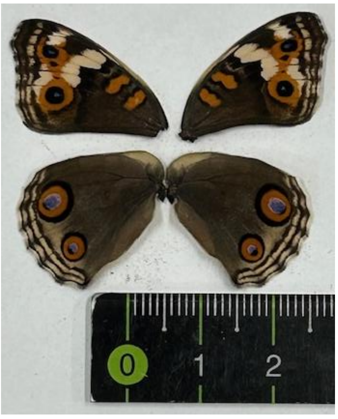

NO3

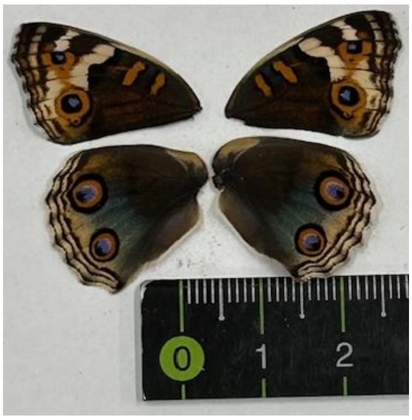

NO4

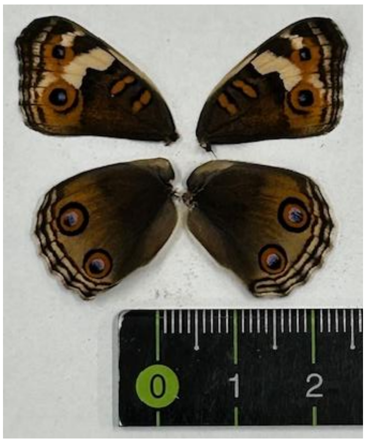

NO5

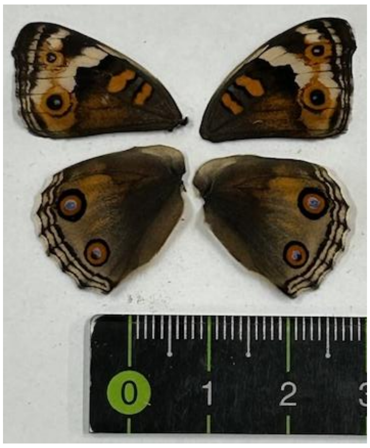

NO6

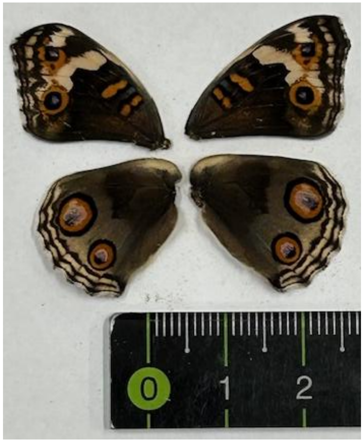

NO7

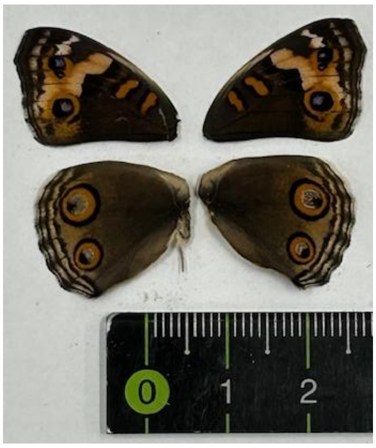

NO8

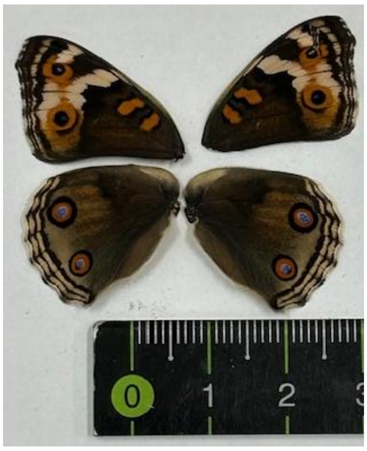

NO9

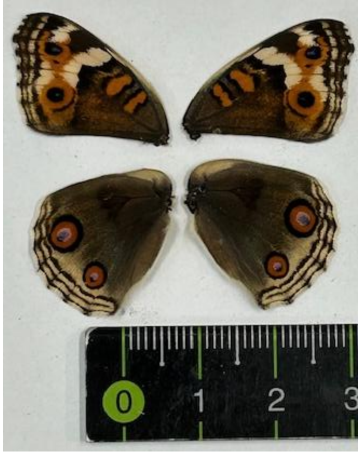

NO10

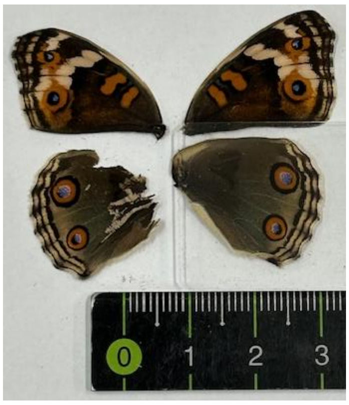

NO11

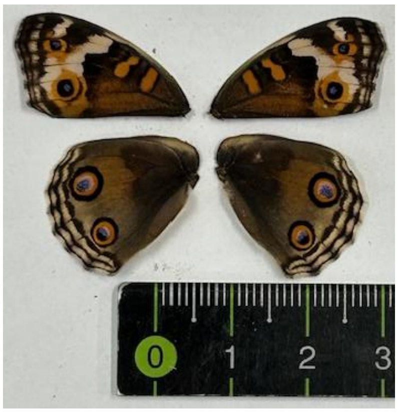

NO12

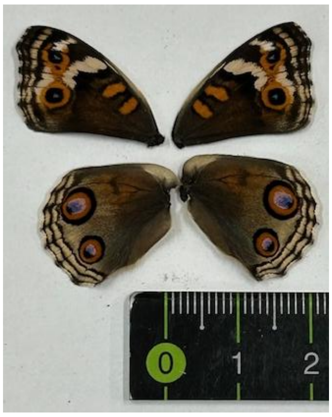

NO13

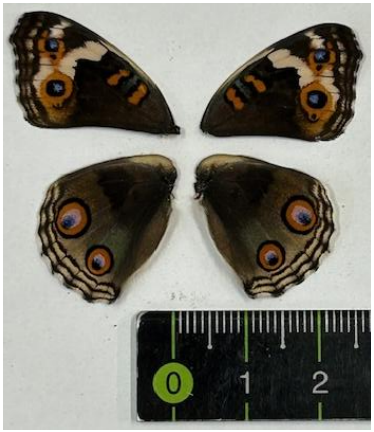

(p) VAS2870 injection (13.87 mM), female, ventral side (n = 13)

NO1

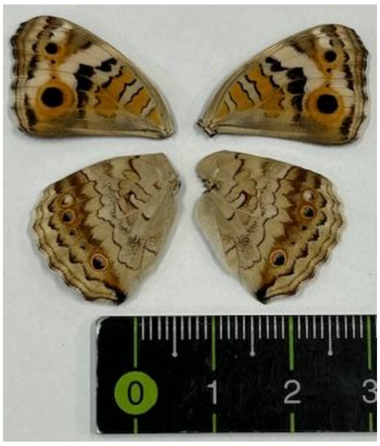

NO2

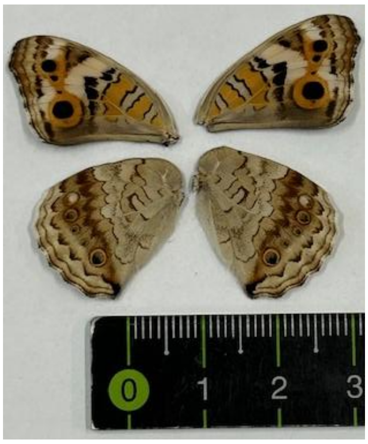

NO3

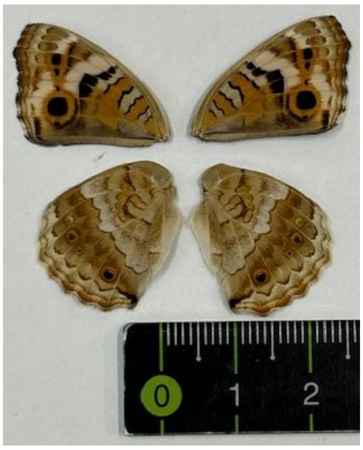

NO4

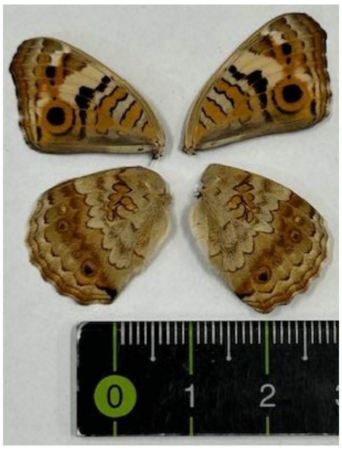

NO5

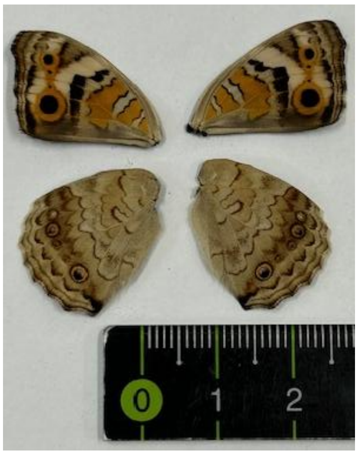

NO6

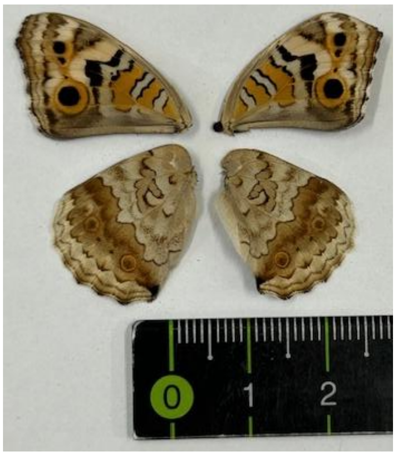

NO7

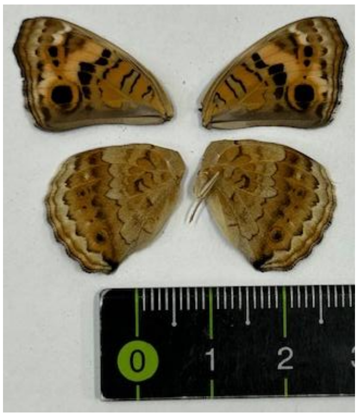

NO8

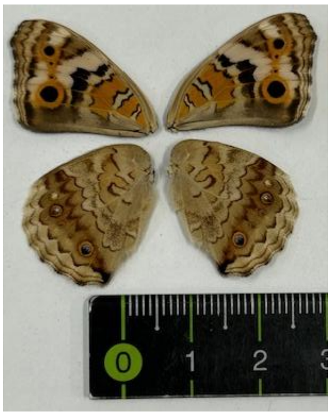

NO9

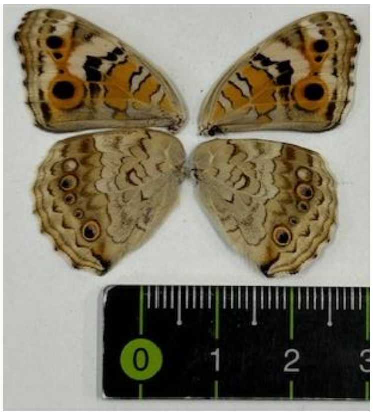

NO10

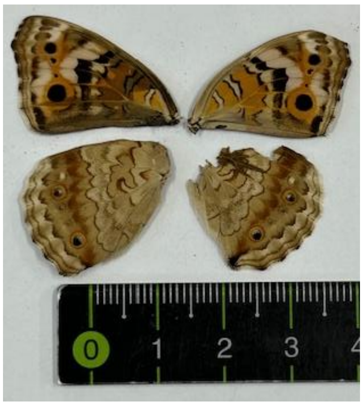

NO11

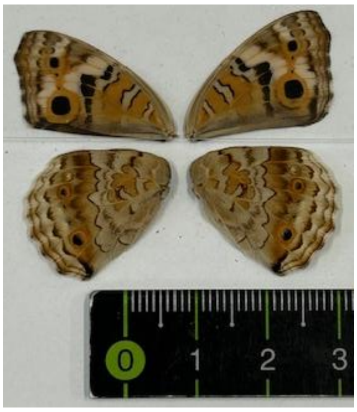

NO12

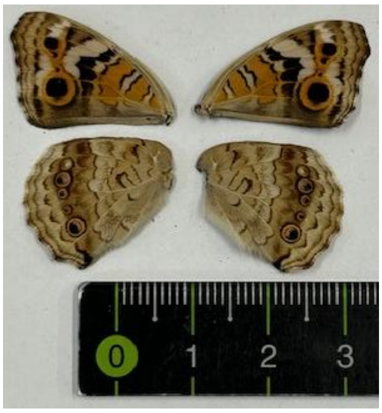

NO13

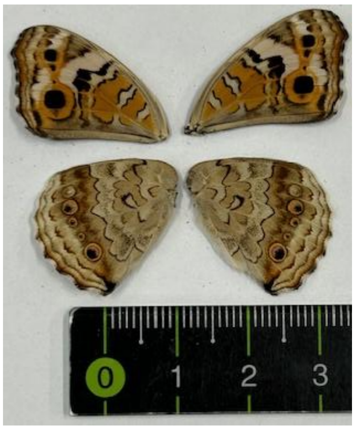

Supplement: Supplementary file 1 [file insects-17-00300-s001.zip › Supplementary Figure S2.pdf]
