# Supplementary material for: Pharmacological Effects of NADPH Oxidase Inhibitors on Butterfly Wing Morphogenesis and Color Pattern Formation in Junonia orithya
Source: Insects. 2026 Mar 10;17(3):300. doi: 10.3390/insects17030300 (PMC13026668; doi:10.3390/insects17030300)

Insects

Pharmacological Effects of the NADPH Oxidase Inhibitors on Butterfly Wing Morphogenesis and Color Pattern Formation in *Junonia orithya*

Yugo Nakazato, Momo Ozaki, Ryunosuke Suenaga, and Joji M. Otaki

The BCPH Unit of Molecular Physiology, Department of Chemistry, Biology and Marine Science, Faculty of Science, University of the Ryukyus, Nishihara, Okinawa 903-0213, Japan.

Supplementary Figure S3. Wings of all butterfly samples used for the VAS2870 injection experiment at 27.75 mM and 69.37 mM (a single sibling group).

(a) No treatment, male, dorsal side ($n = 24$)

NO1

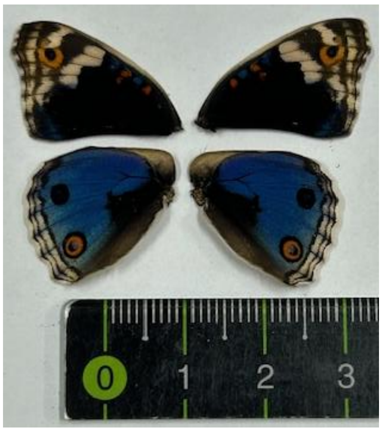

NO2

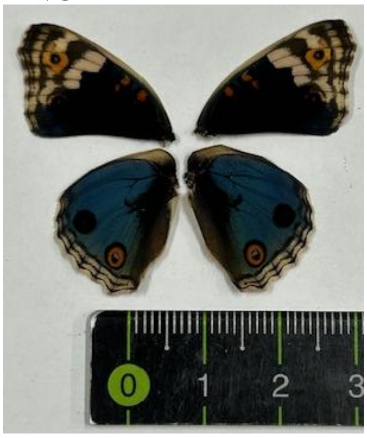

NO3

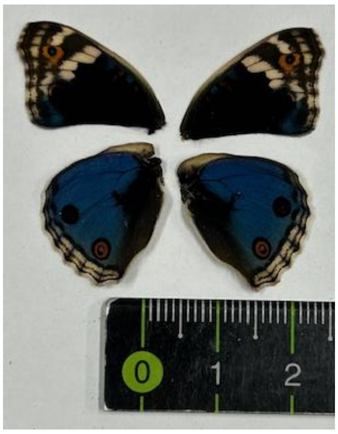

NO4

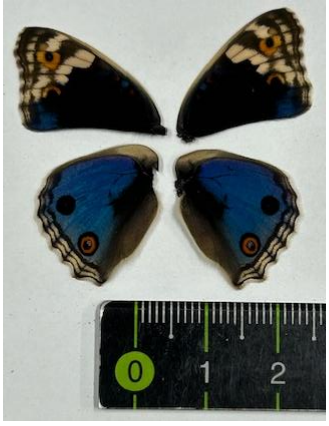

NO5

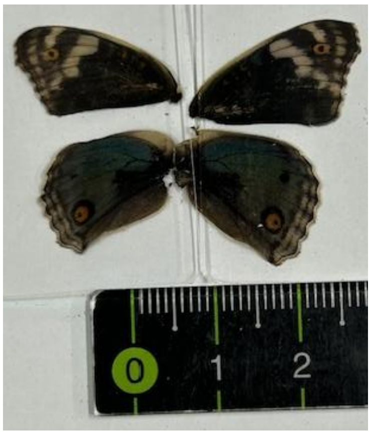

NO6

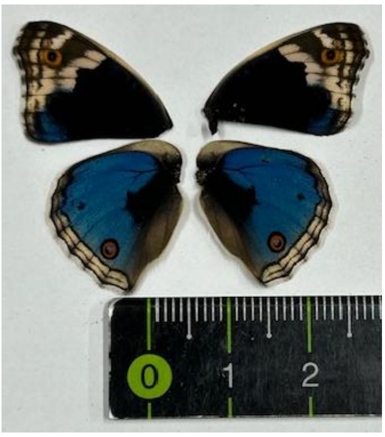

NO7

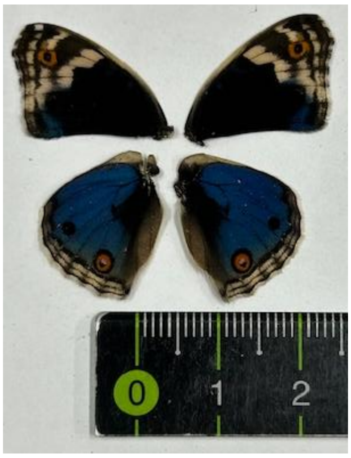

NO8

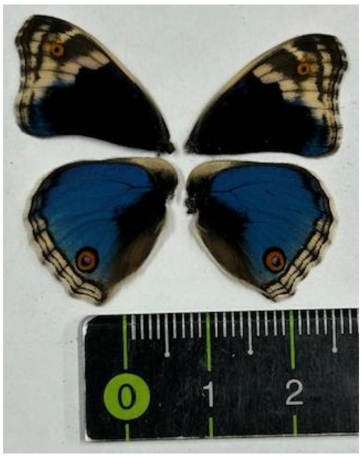

NO9

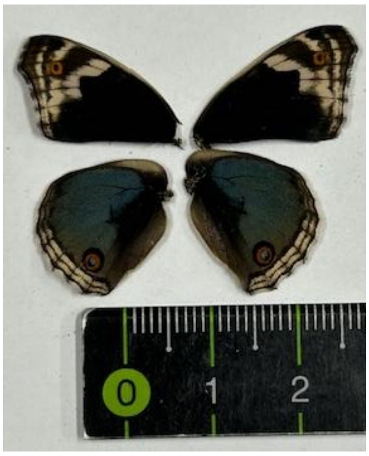

NO10

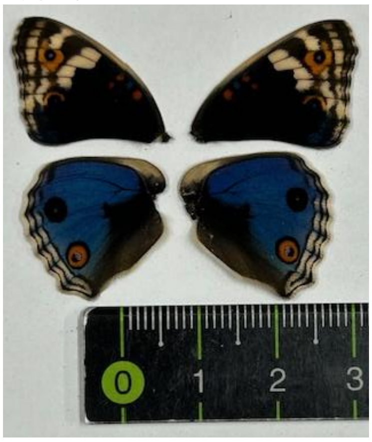

NO11

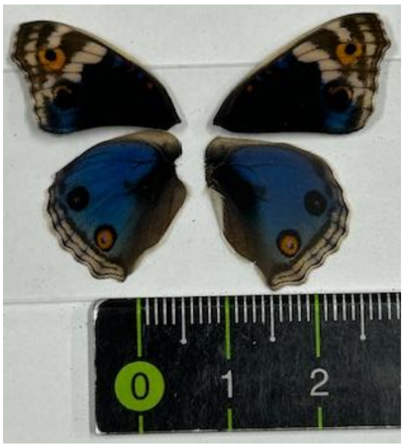

NO12

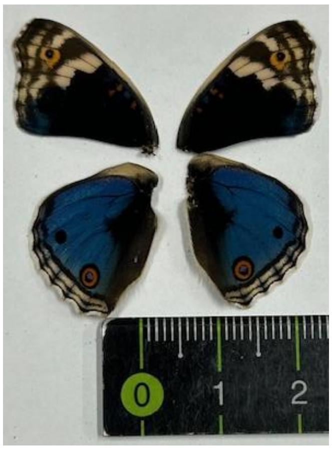

NO13

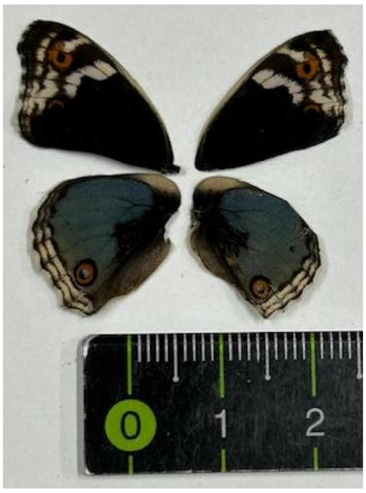

NO14

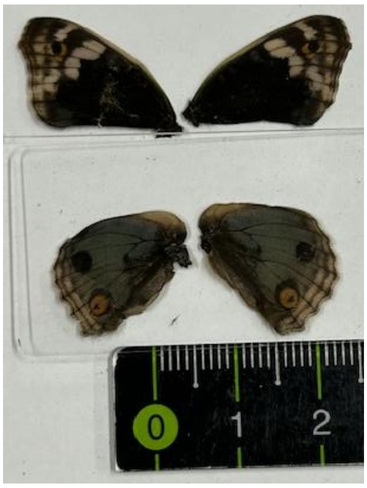

NO15

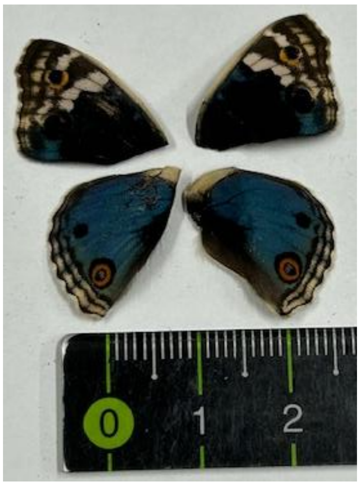

NO16

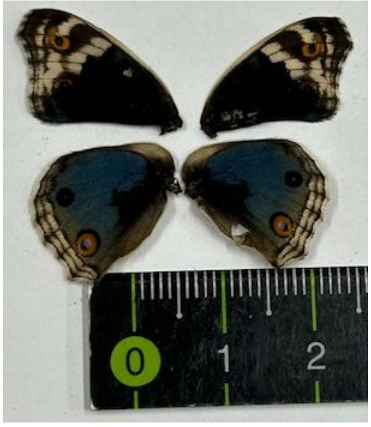

NO17

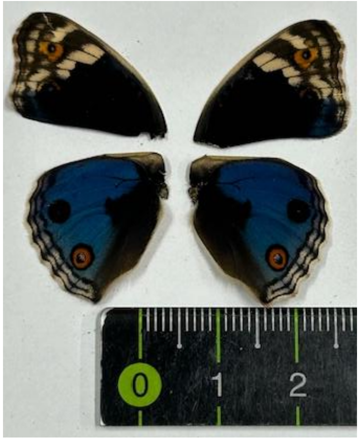

NO18

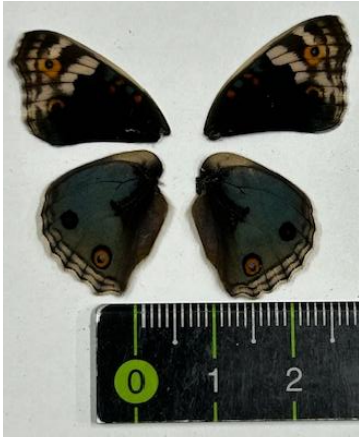

NO19

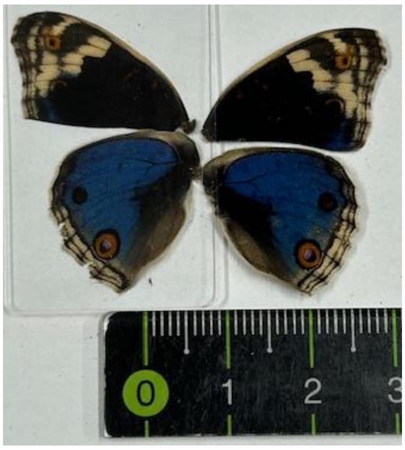

NO20

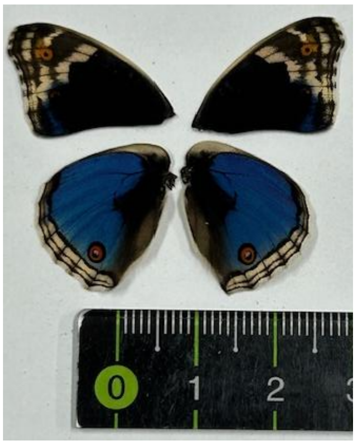

NO21

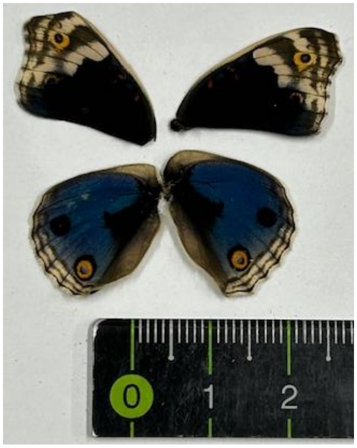

NO22

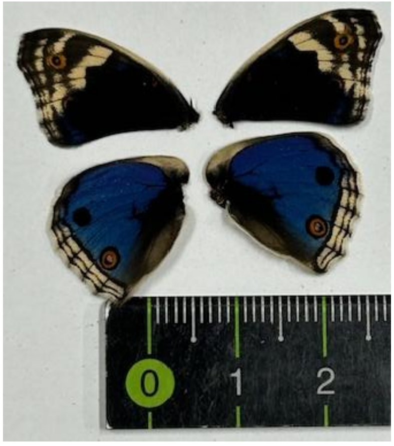

NO23

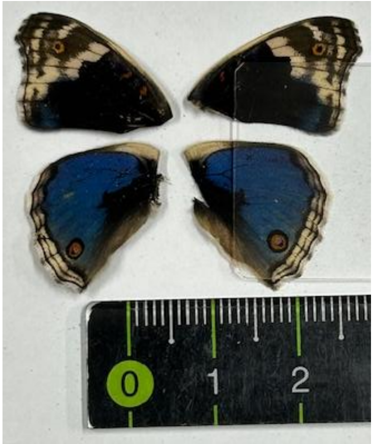

NO24

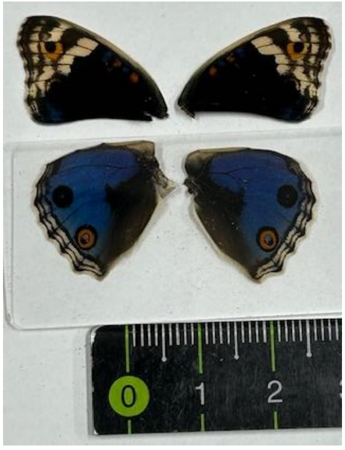

(b) No treatment, male, ventral side (n = 24)

NO1

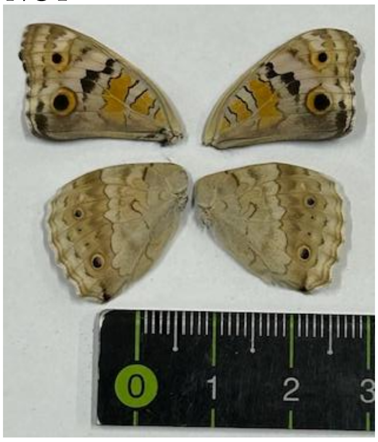

NO2

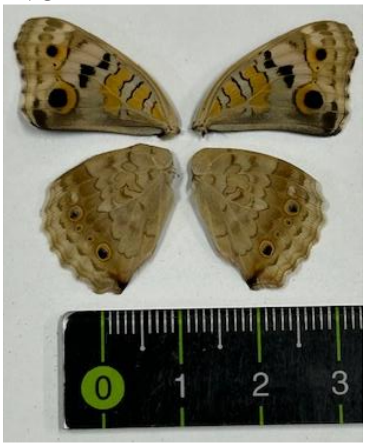

NO3

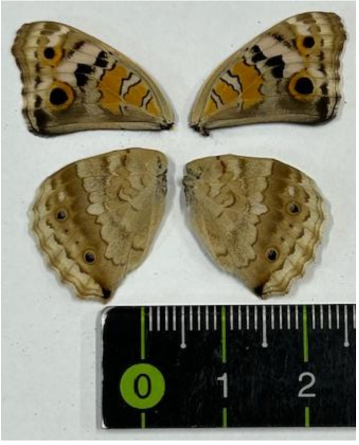

NO4

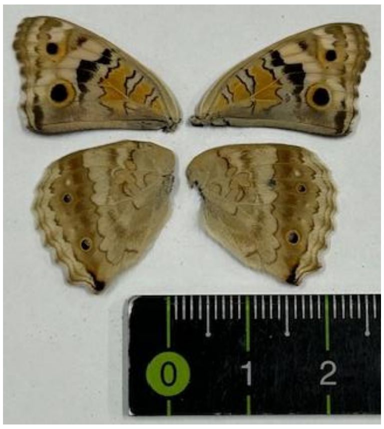

NO5

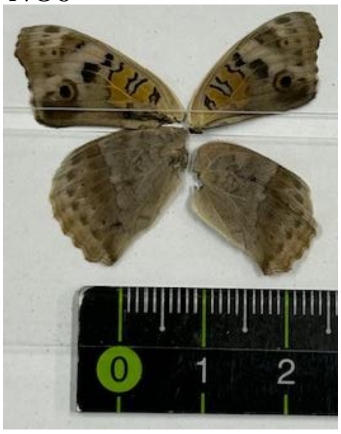

NO6

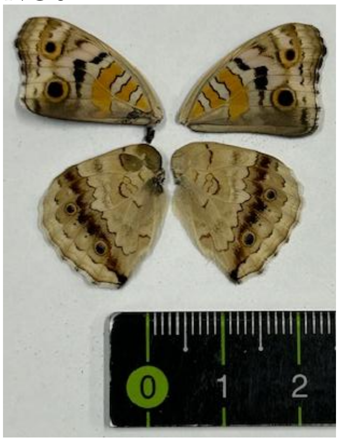

NO7

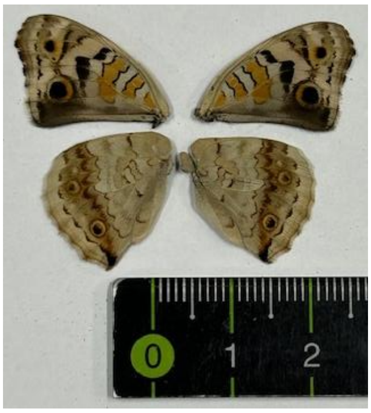

NO8

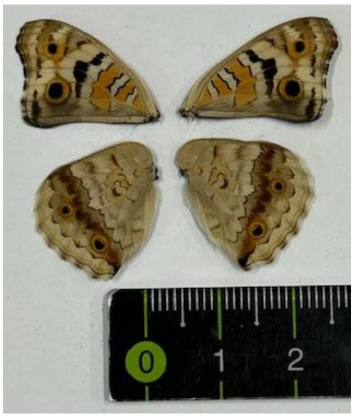

NO9

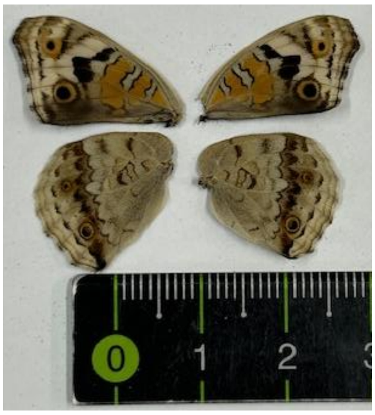

NO10

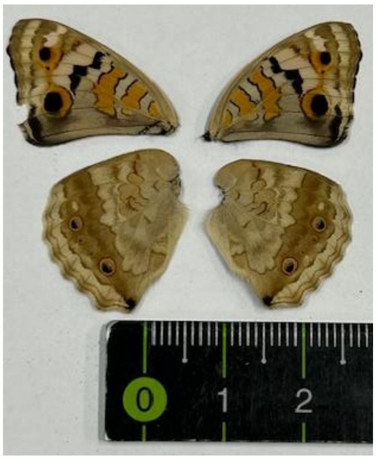

NO11

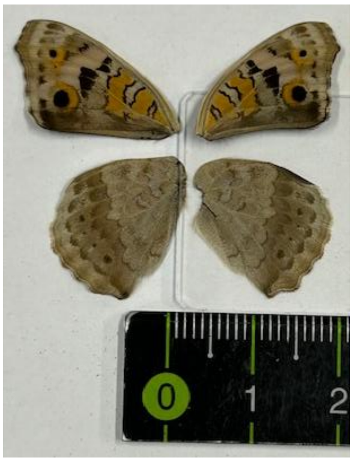

NO12

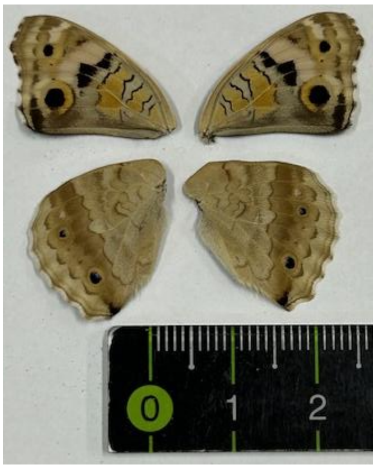

NO13

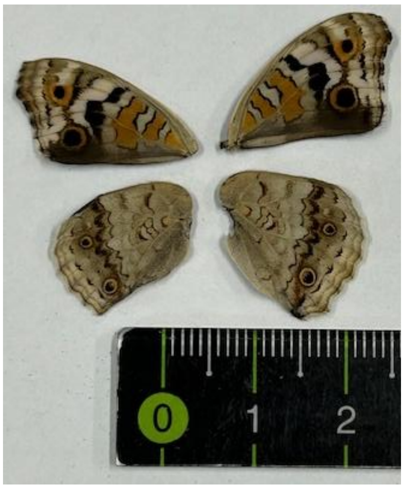

NO14

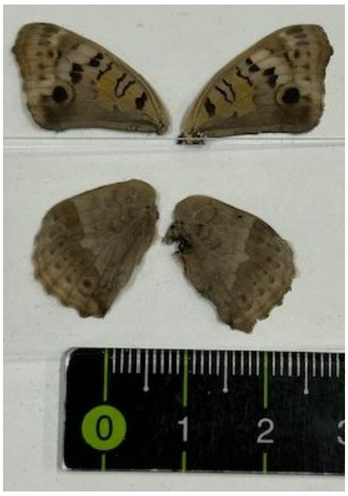

NO15

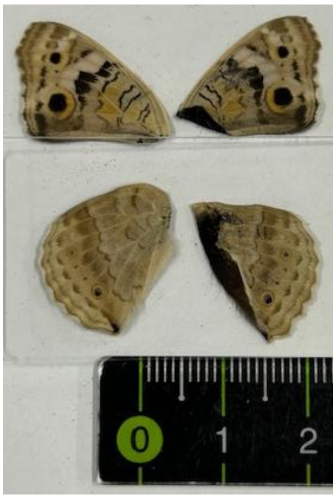

NO16

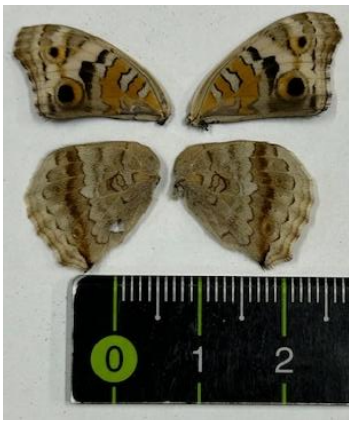

NO17

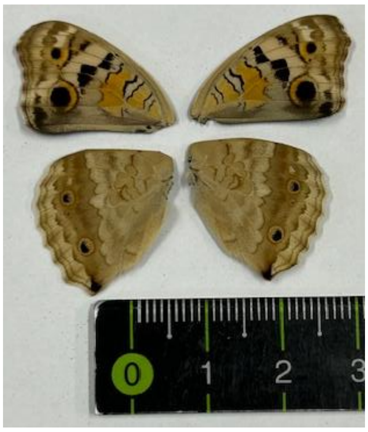

NO18

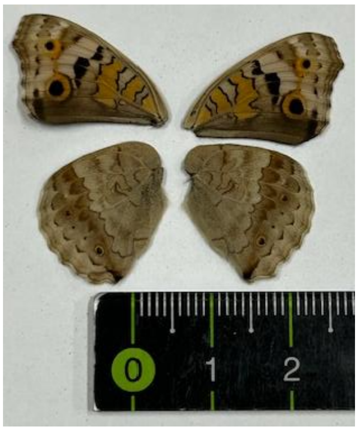

NO19

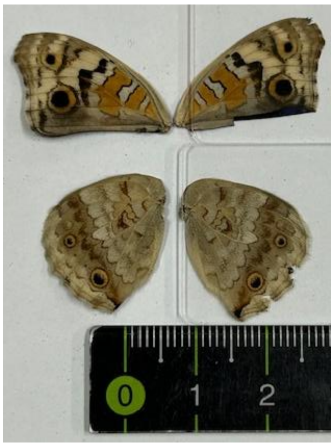

NO20

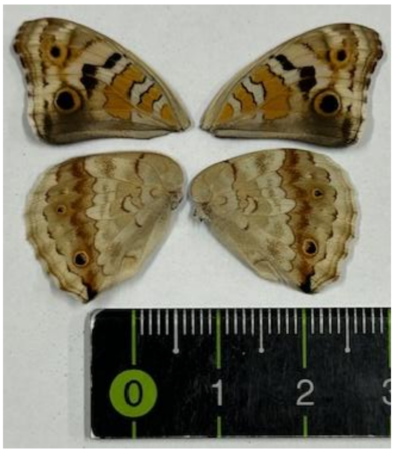

NO21

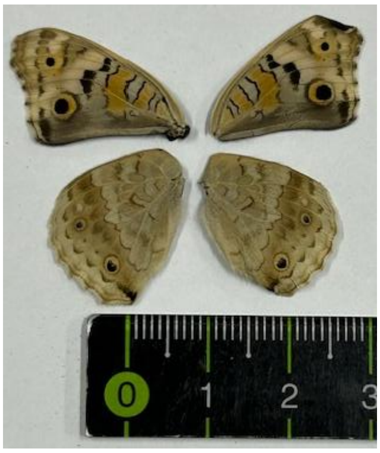

NO22

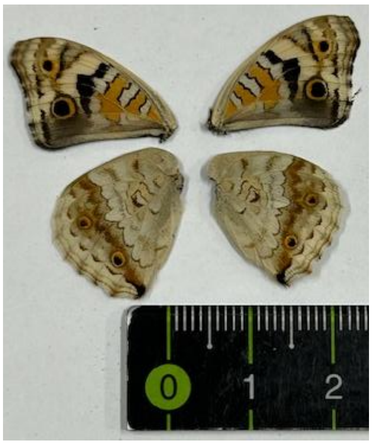

NO23

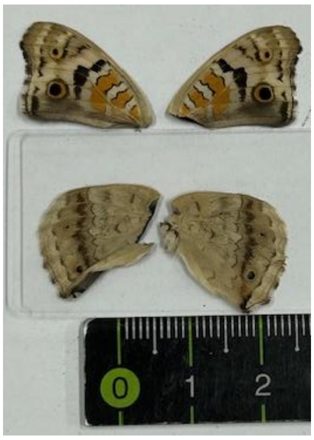

NO24

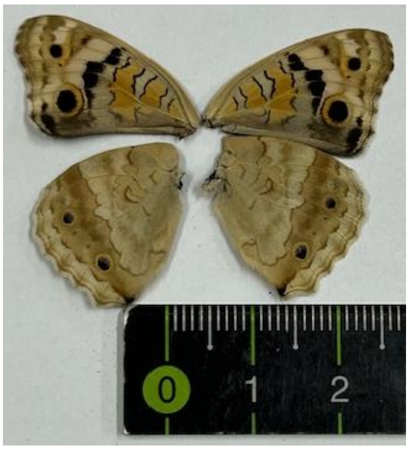

(c) No treatment, female, dorsal side ($n = 25$)

NO1

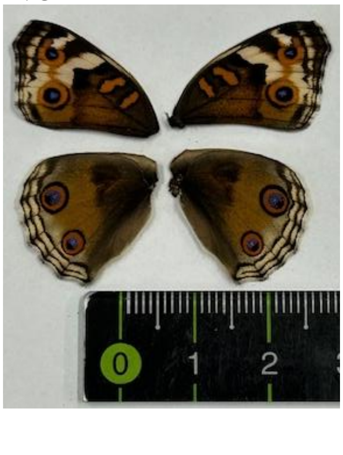

NO2

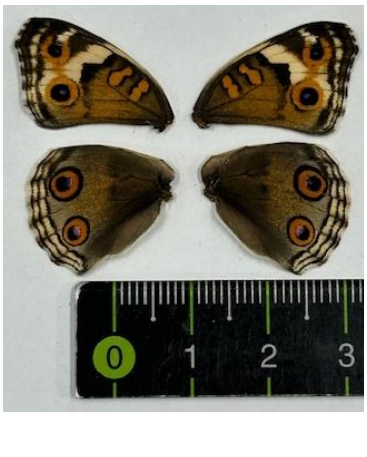

NO3

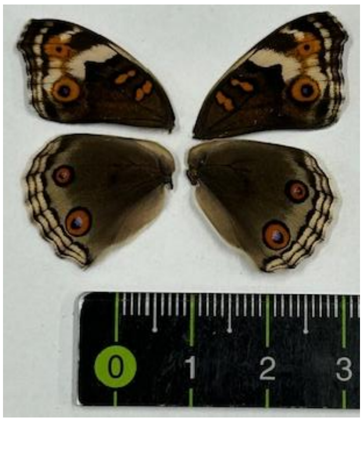

NO4

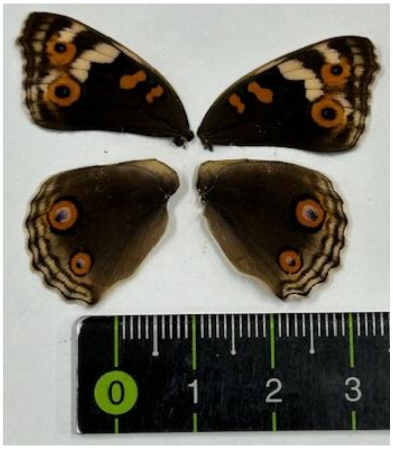

NO5

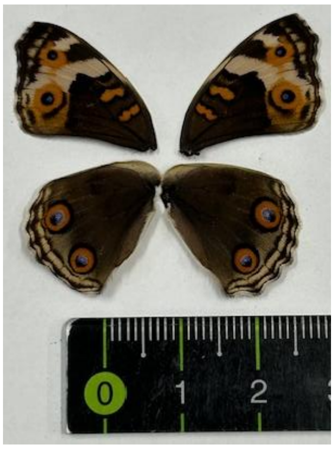

NO6

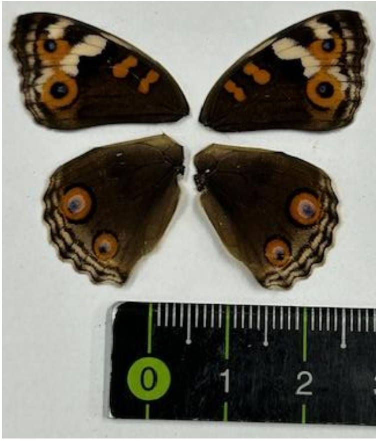

NO7

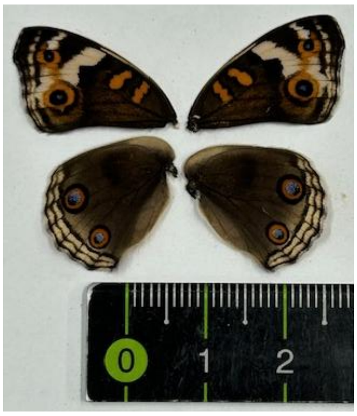

NO8

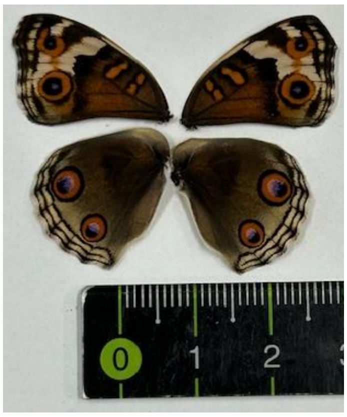

NO9

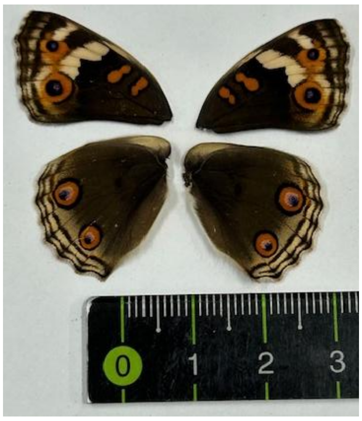

NO10

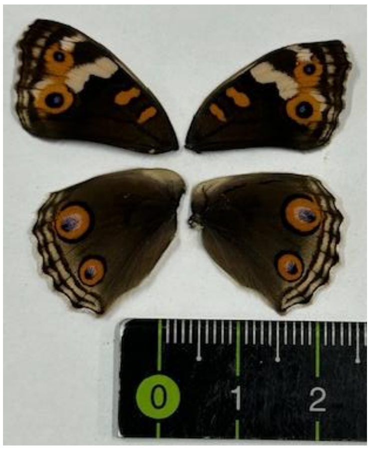

NO11

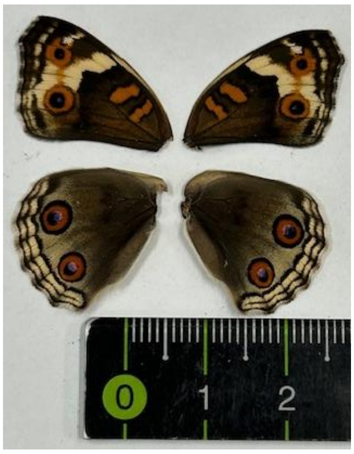

NO12

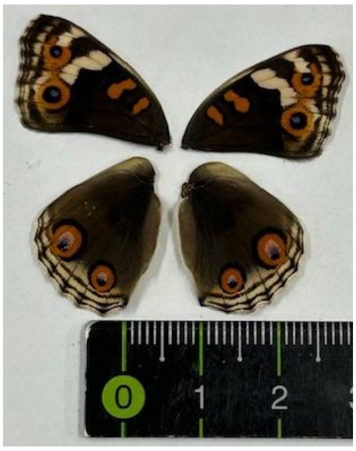

NO13

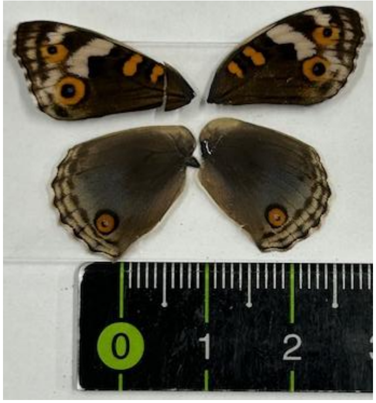

NO14

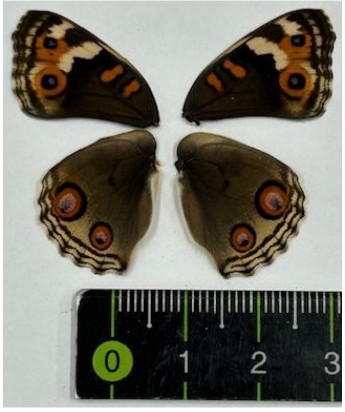

NO15

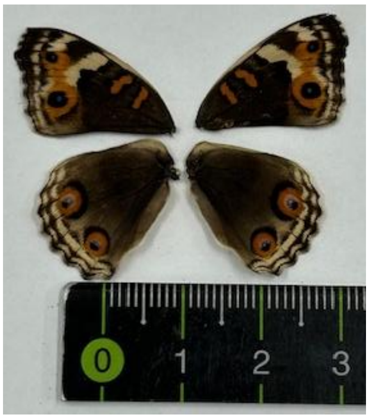

NO16

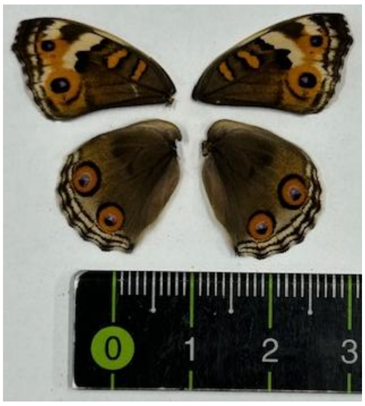

NO17

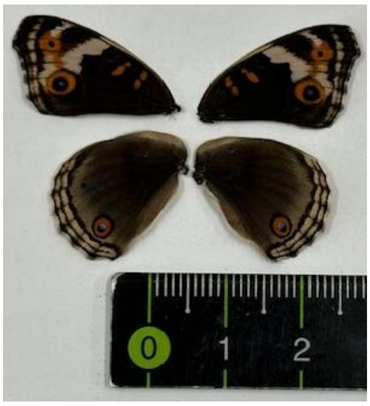

NO18

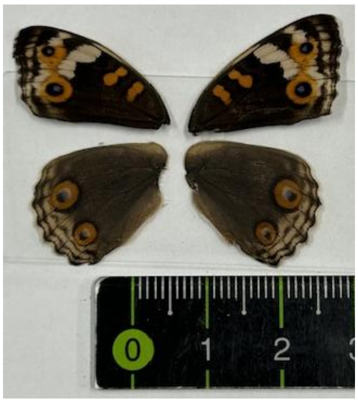

NO19

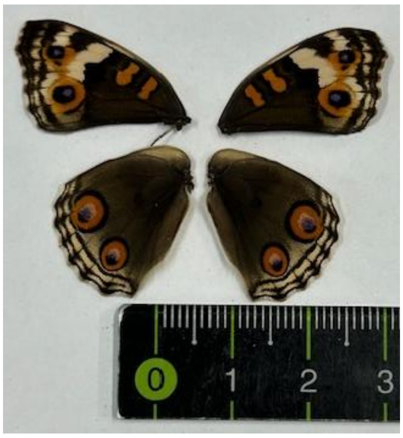

NO20

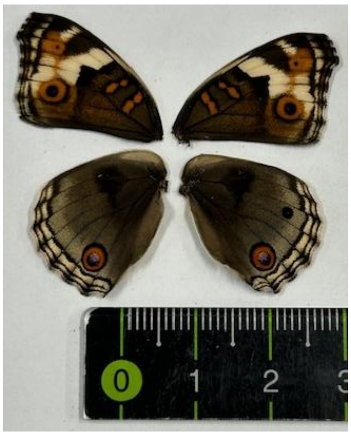

NO21

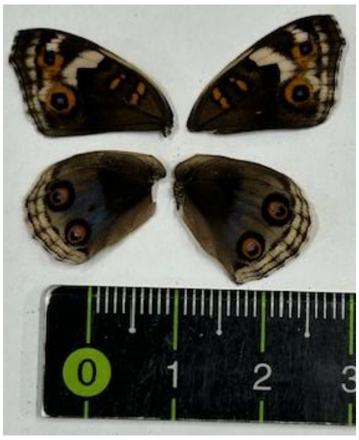

NO22

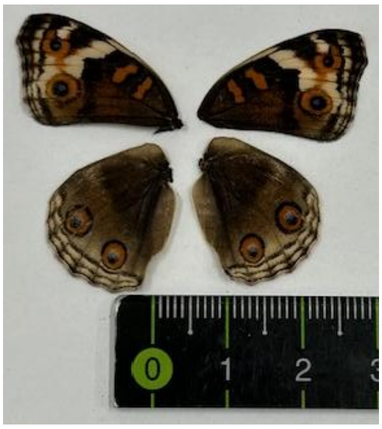

NO23

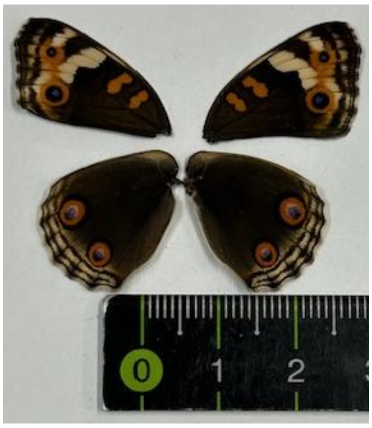

NO24

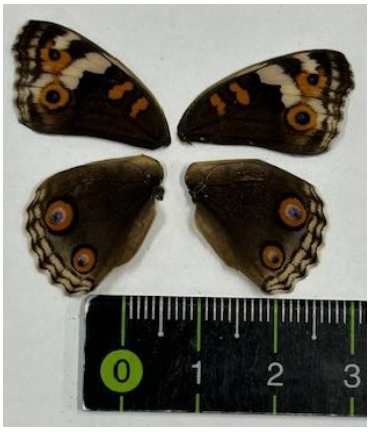

NO25

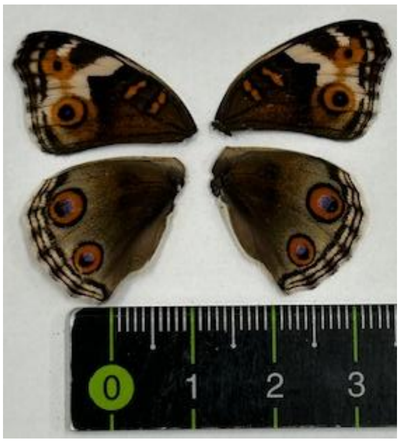

(d) No treatment, female, ventral side (n = 25)

NO1

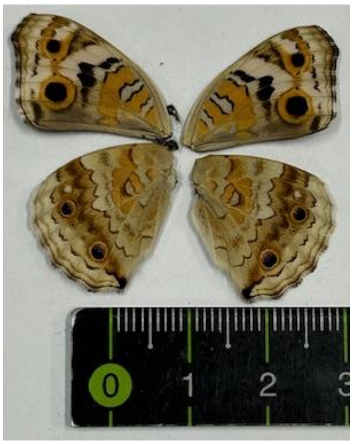

NO2

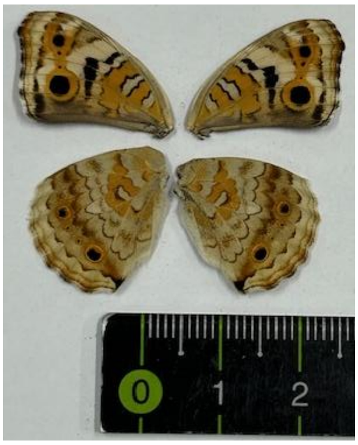

NO3

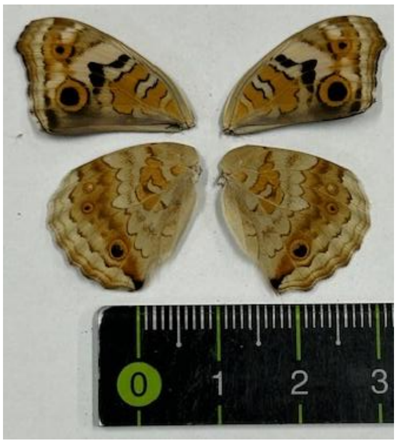

NO4

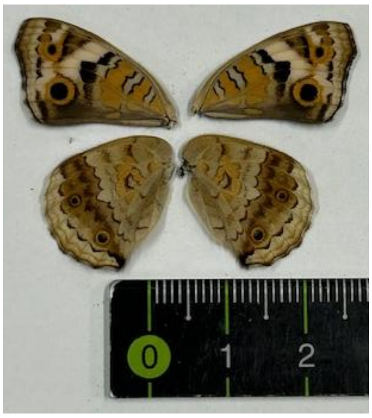

NO5

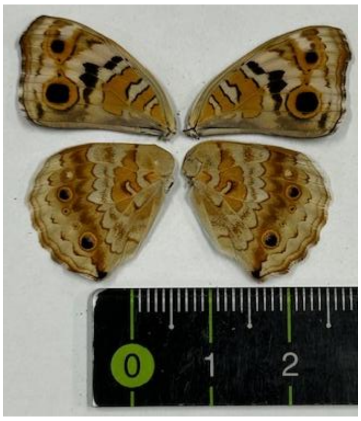

NO6

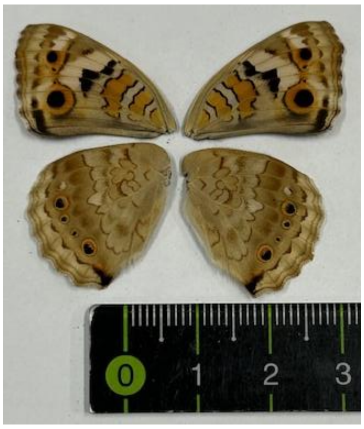

NO7

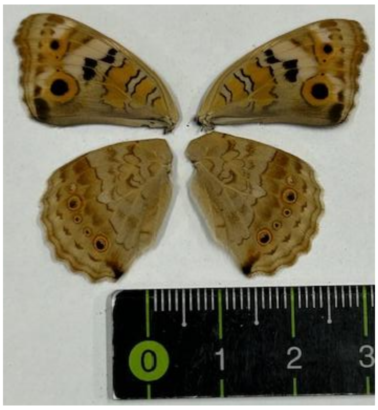

NO8

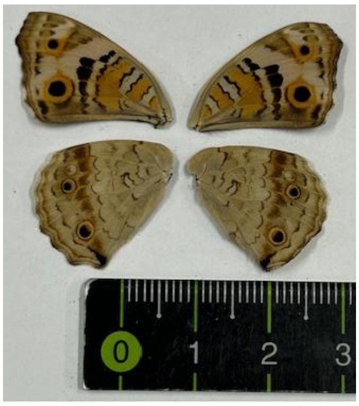

NO9

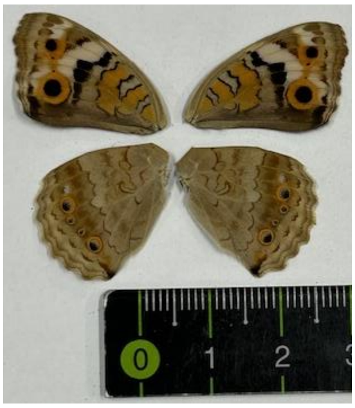

NO10

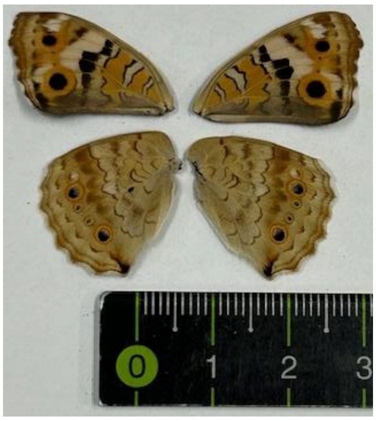

NO11

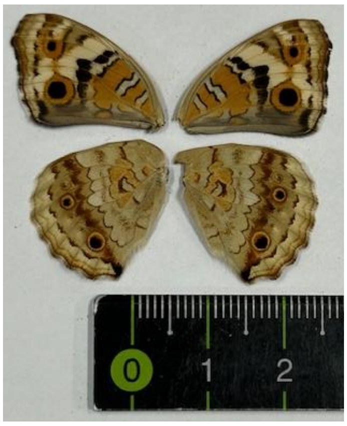

NO12

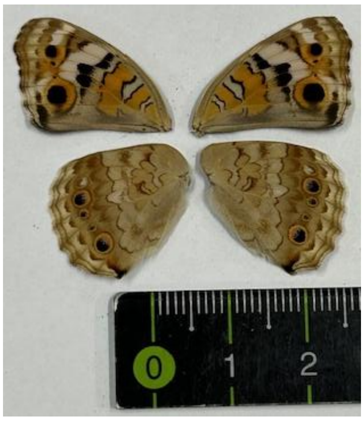

NO13

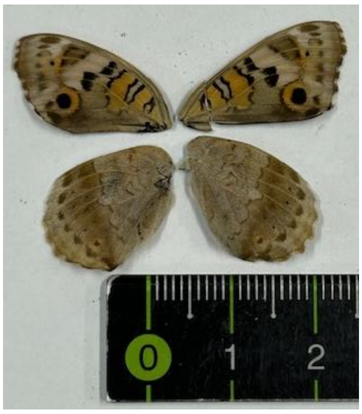

NO14

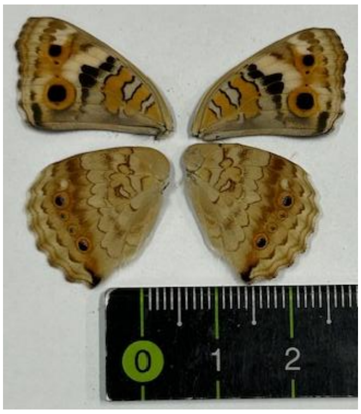

NO15

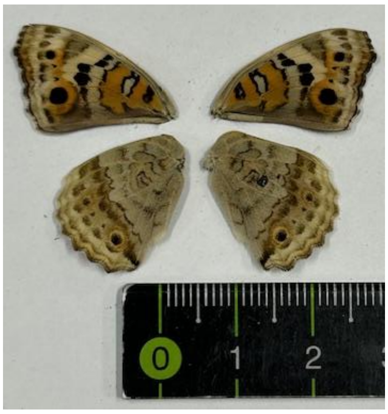

NO16

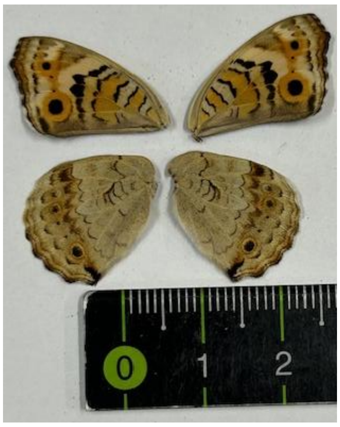

NO17

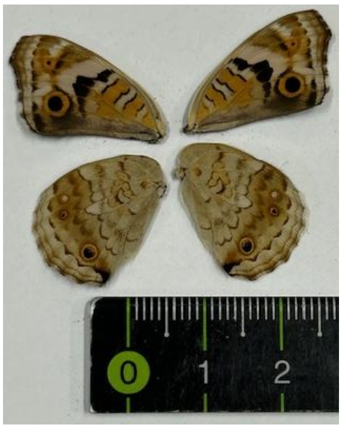

NO18

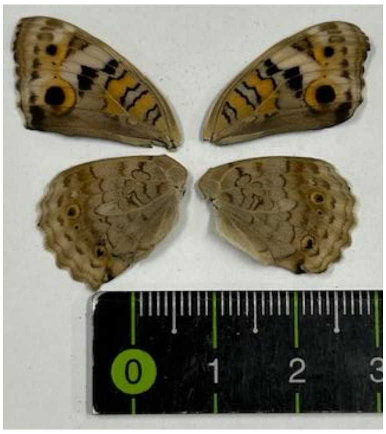

NO19

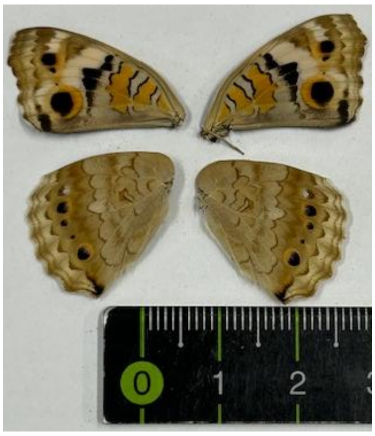

NO20

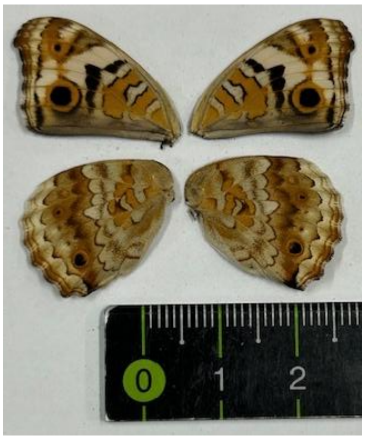

NO21

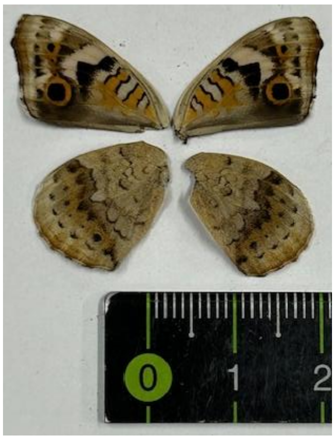

NO22

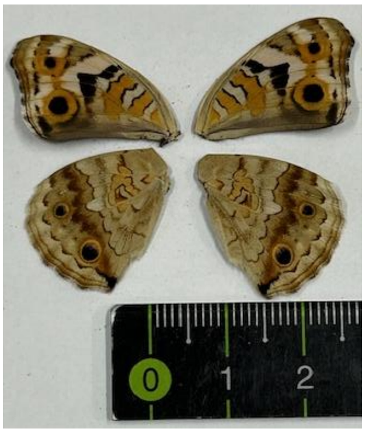

NO23

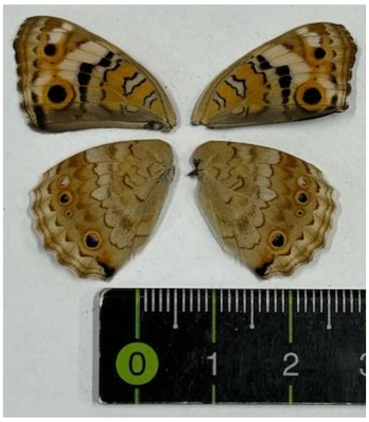

NO24

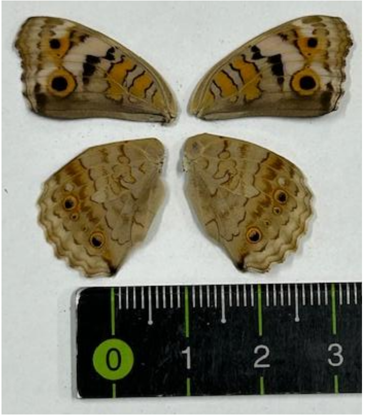

NO25

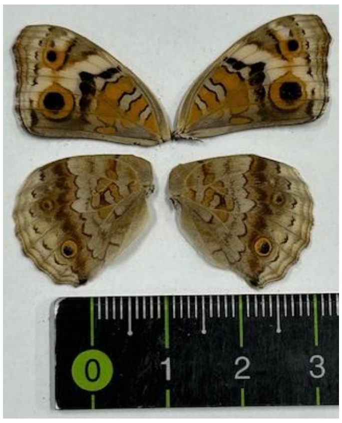

(e) VAS2870 injection (27.75mM), male, dorsal side (n = 9)

NO1

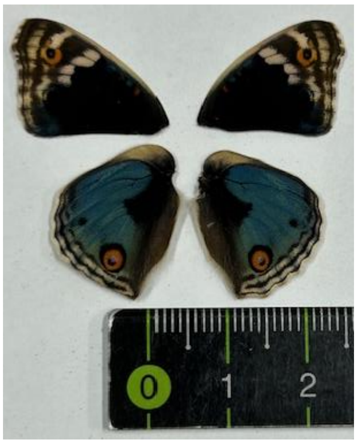

NO2

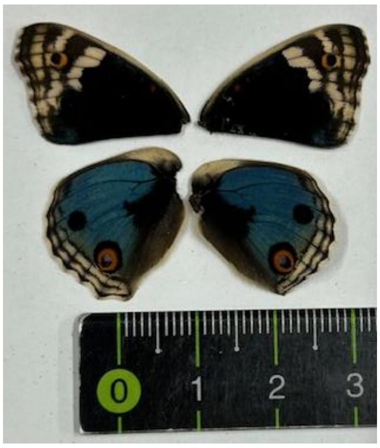

NO3

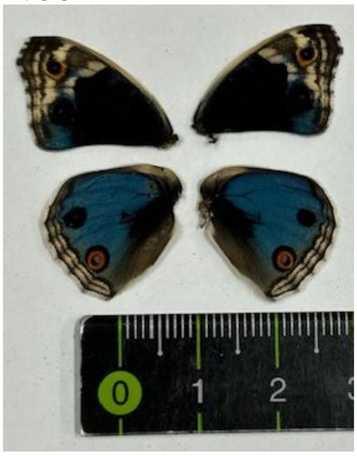

NO4

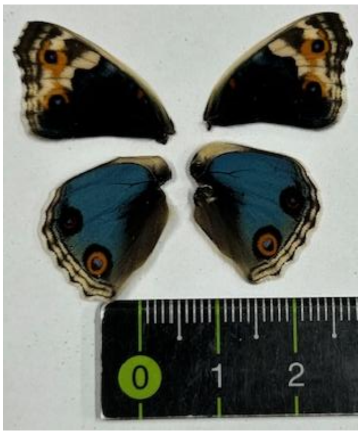

NO5

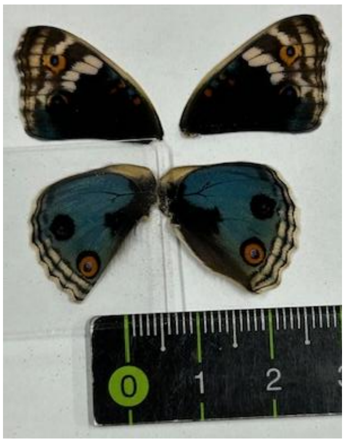

NO6

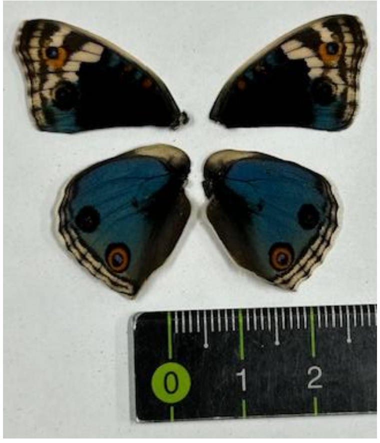

NO7

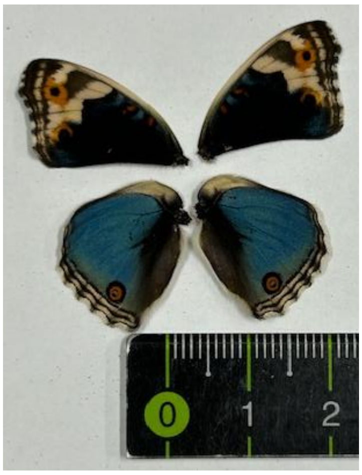

NO8

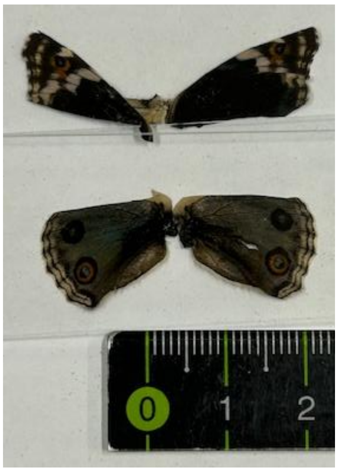

NO9

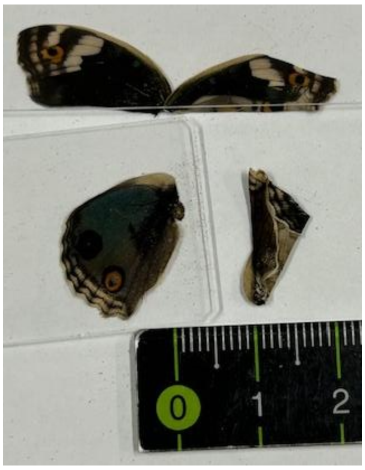

(f) VAS2870 injection (27.75 mM), male, ventral side ($n = 9$)

NO1

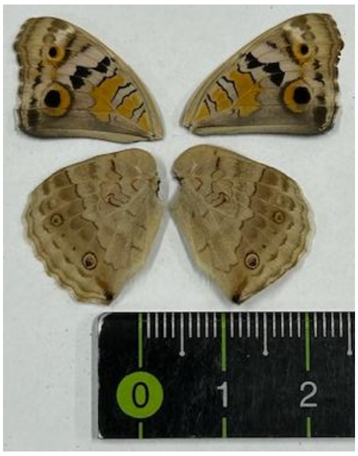

NO2

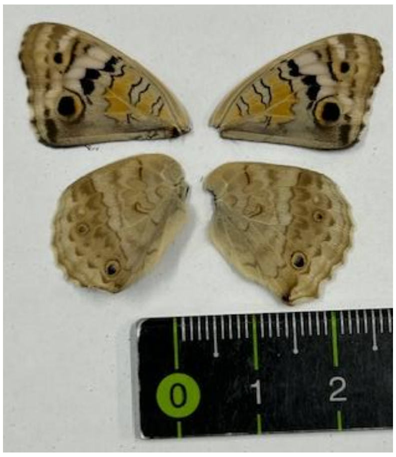

NO3

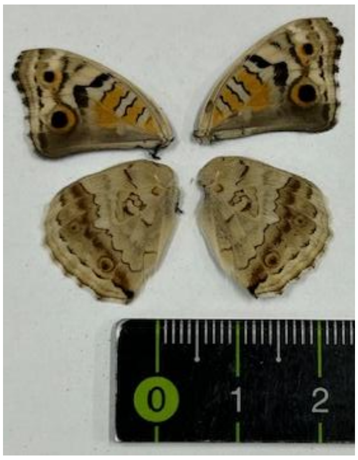

NO4

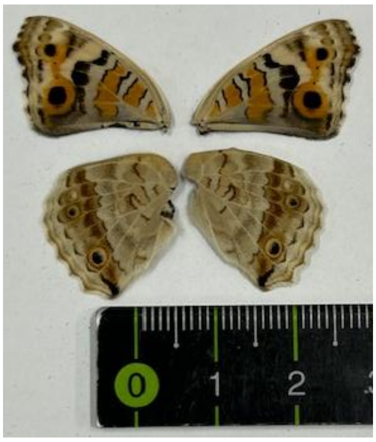

NO5

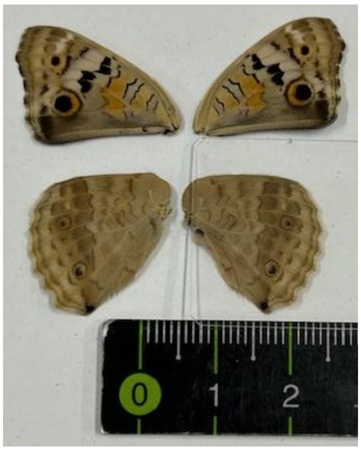

NO6

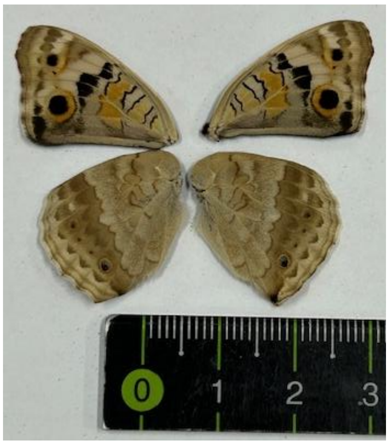

NO7

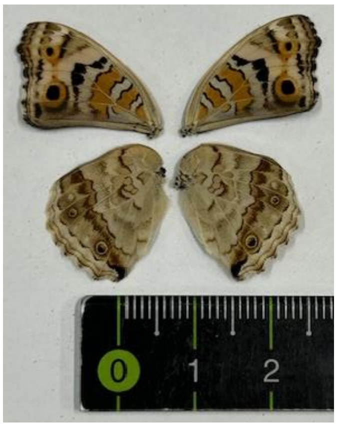

NO8

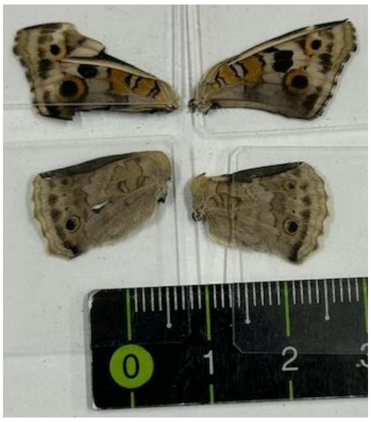

NO9

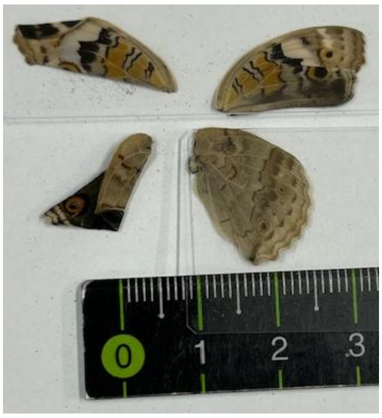

(g) VAS2870 injection (27.75mM), female, dorsal side ($n = 7$)

NO1

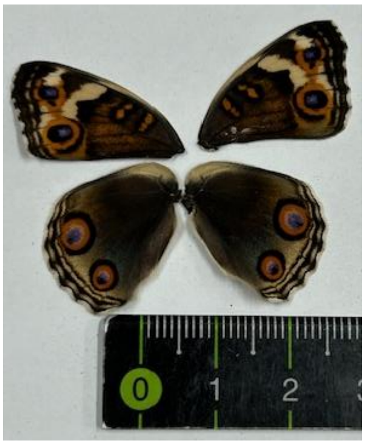

NO2

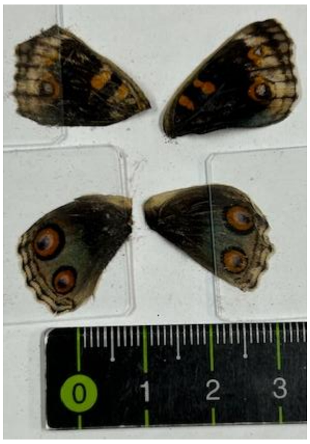

NO3

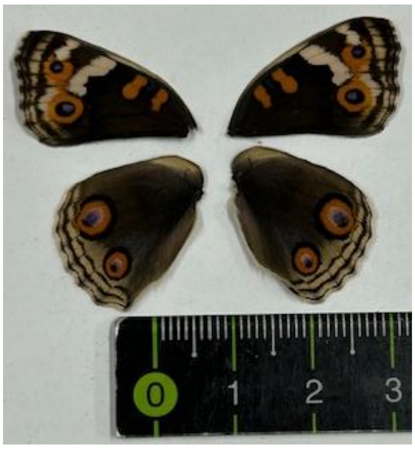

NO4

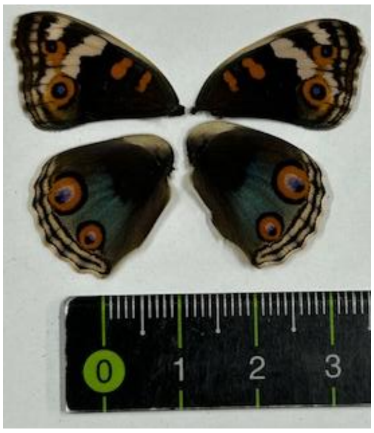

NO5

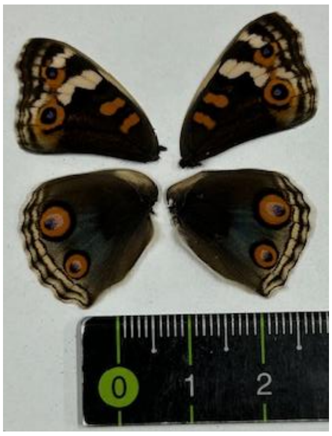

NO6

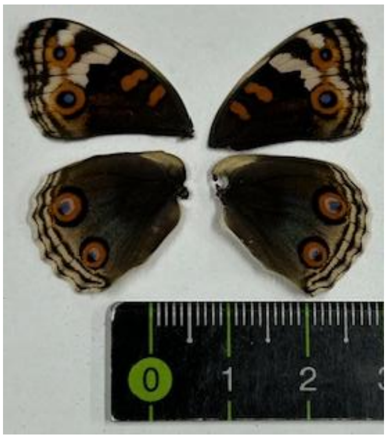

NO7

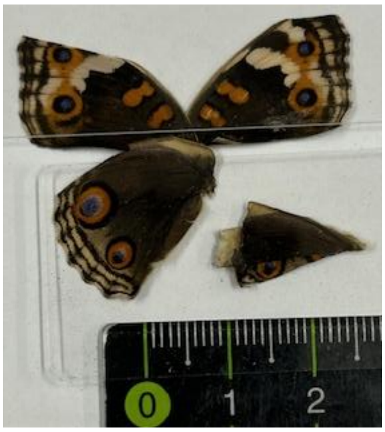

(h) VAS2870 injection (27.75mM), female, ventral side (n = 7)

NO1

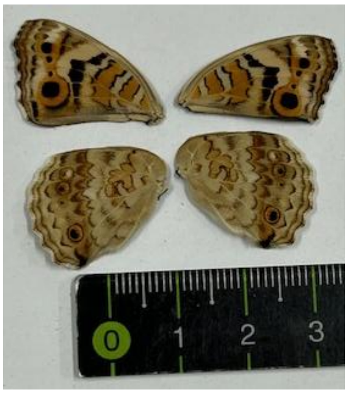

NO2

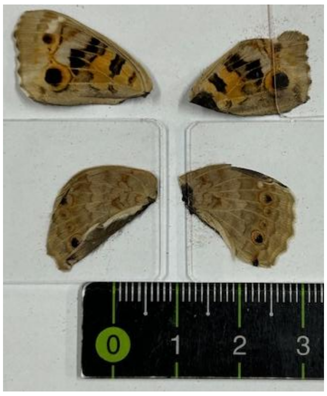

NO3

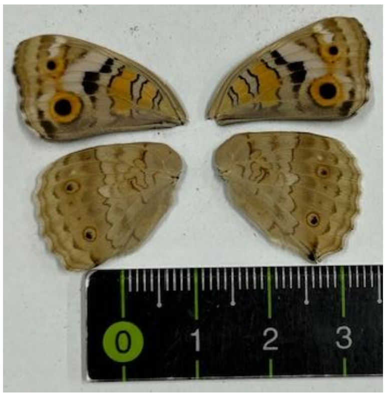

NO4

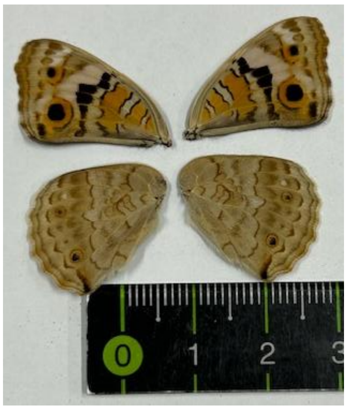

NO5

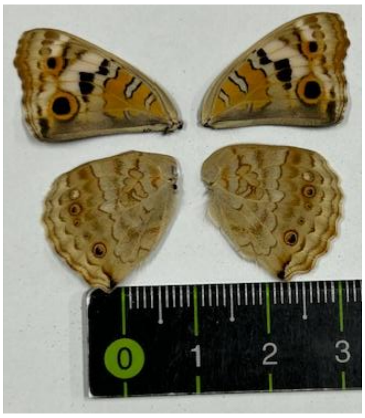

NO6

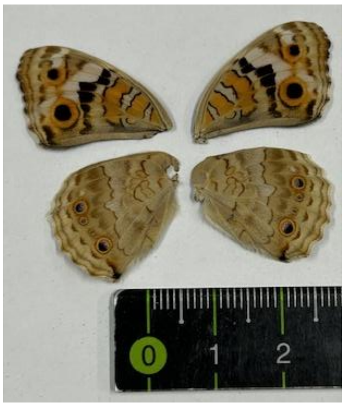

NO7

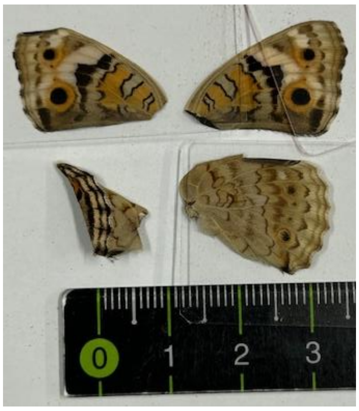

VAS2870 injection (27.75 mM), NO 2

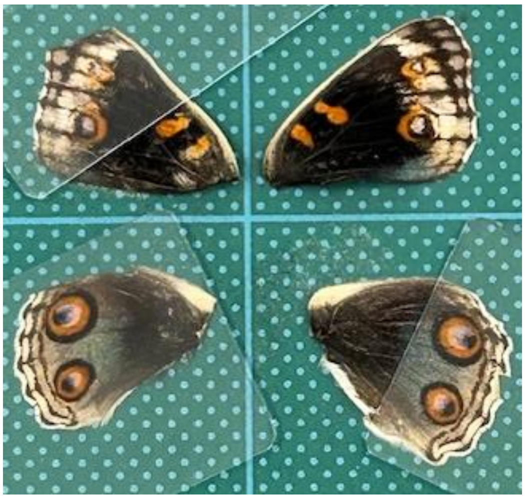

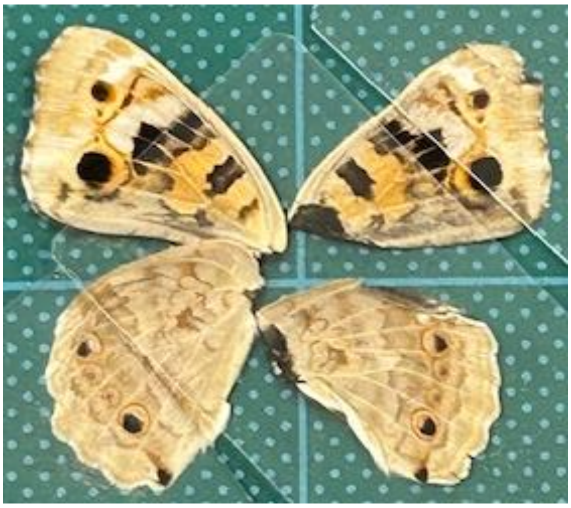

(i) VAS2870 injection (69.37mM), male ($n = 1$) and female ($n = 1$)

Male

NO1：Dorsal

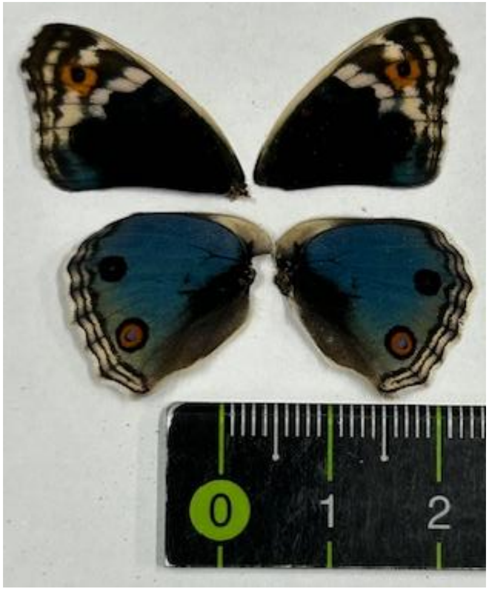

NO1：Ventral

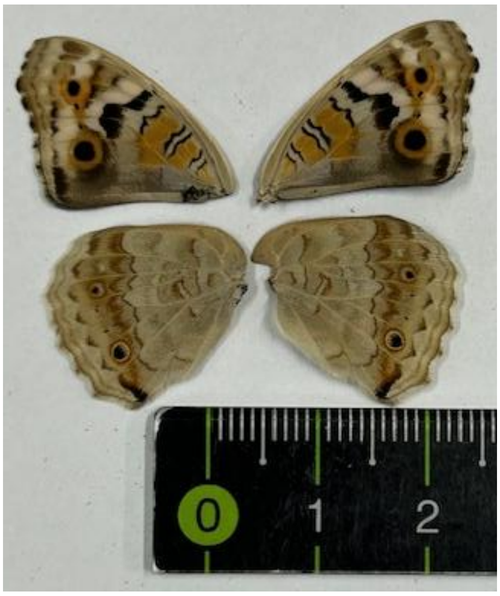

Female

NO1：Dorsal

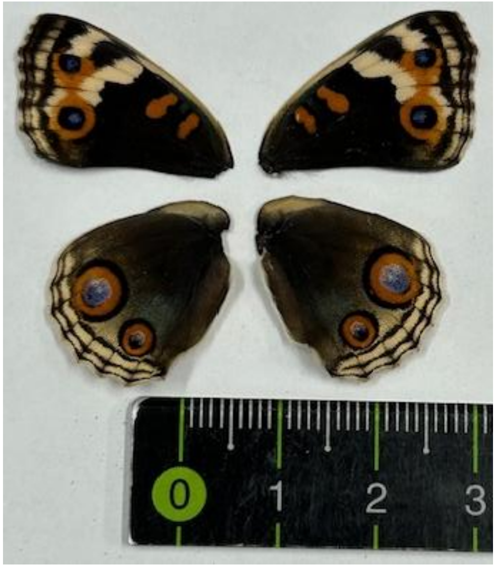

NO1：Ventral

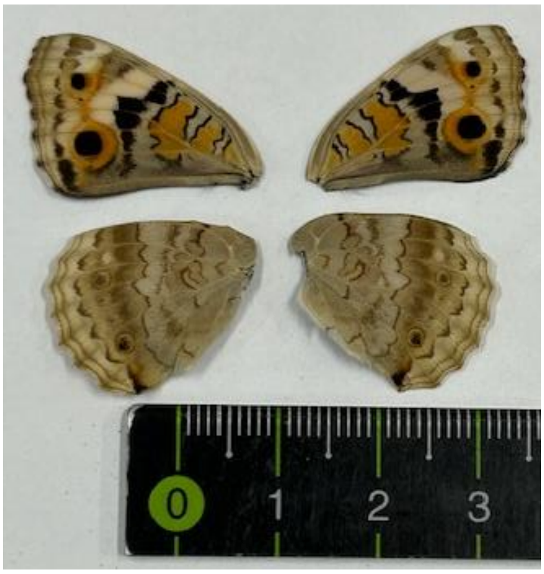

Supplement: Supplementary file 1 [file insects-17-00300-s001.zip › Supplementary Figure S3.pdf]
