# Supplementary material for: Pharmacological Effects of NADPH Oxidase Inhibitors on Butterfly Wing Morphogenesis and Color Pattern Formation in Junonia orithya
Source: Insects. 2026 Mar 10;17(3):300. doi: 10.3390/insects17030300 (PMC13026668; doi:10.3390/insects17030300)

Pharmacological Effects of the NADPH Oxidase Inhibitors on Butterfly Wing Morphogenesis and Color Pattern Formation in *Junonia orithya*

Yugo Nakazato, Momo Ozaki, Ryunosuke Suenaga, and Joji M. Otaki

The BCPH Unit of Molecular Physiology, Department of Chemistry, Biology and Marine Science, Faculty of Science, University of the Ryukyus, Nishihara, Okinawa 903-0213, Japan.

Supplementary Figure S5. DPI-treated wings. All wing samples (excluding 41.32 mM) are shown. Note that all wing samples treated with 41.32 mM are shown in Figure 10. Left wings were treated, and right wings were not treated. Only the dorsal hindwings are shown. (**a**) Males (4.13 mM, *n* = 22). (**b**) Females (4.13 mM, *n* = 14). (**c**) Males (0.41 mM, *n* = 6). (**d**) Females (0.41 mM, *n* = 6).

(**a**) Males (4.13 mM, *n* = 22).

No. 1

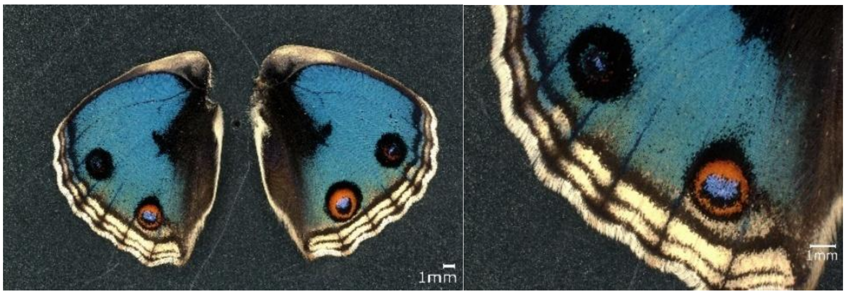

No. 2

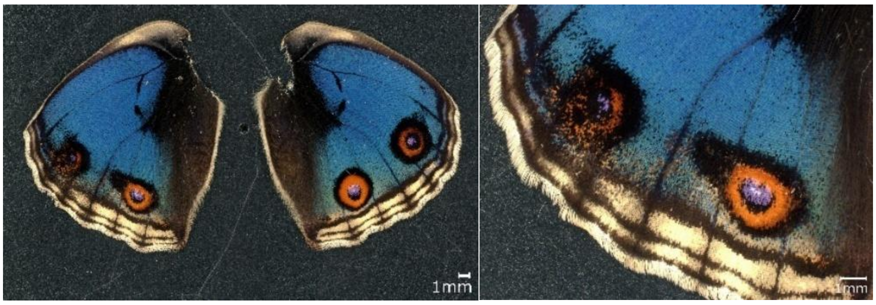

No. 3

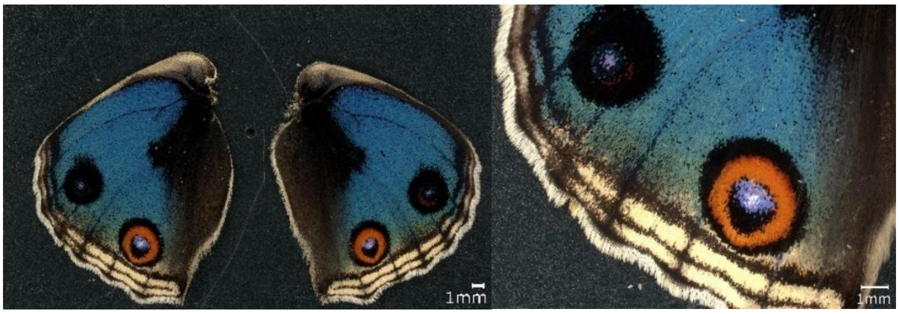

No. 4

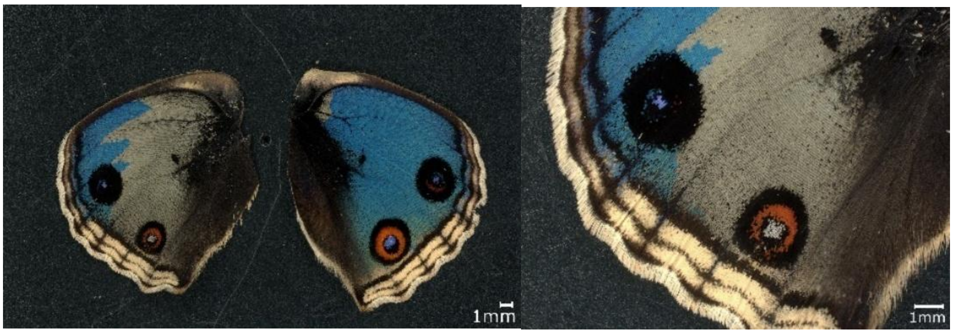

No. 5

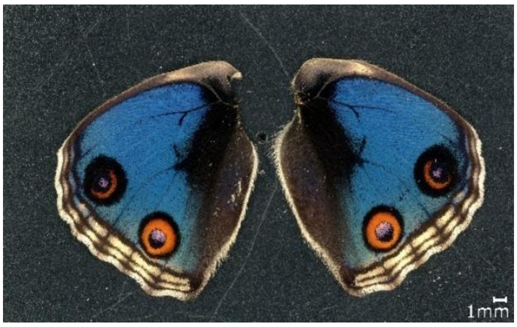

No. 6

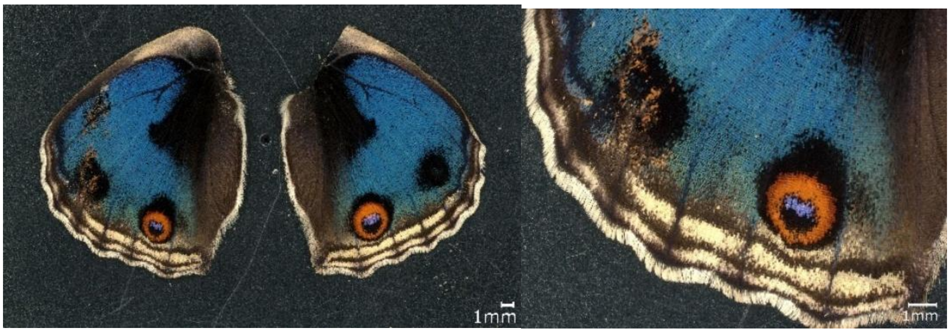

No. 7

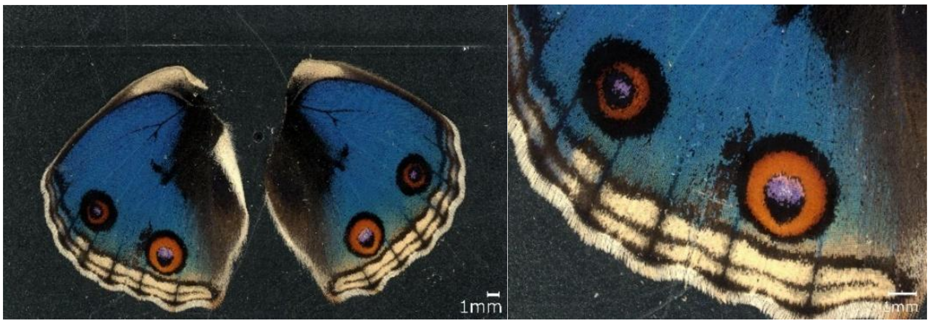

No. 8

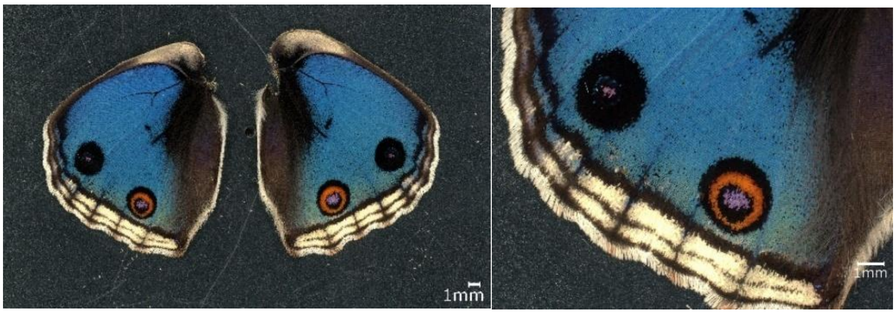

No. 9

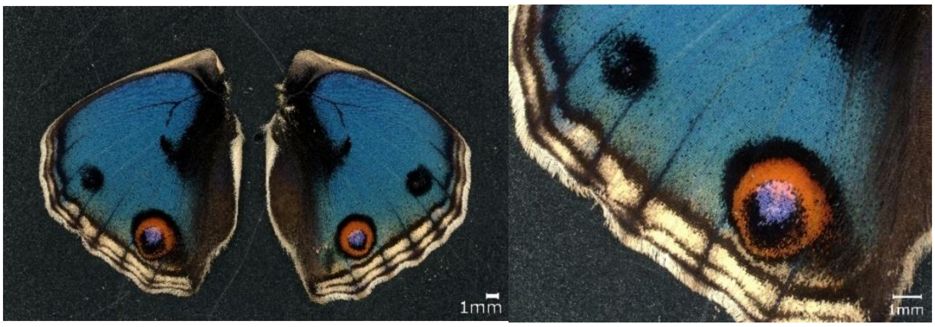

No. 10

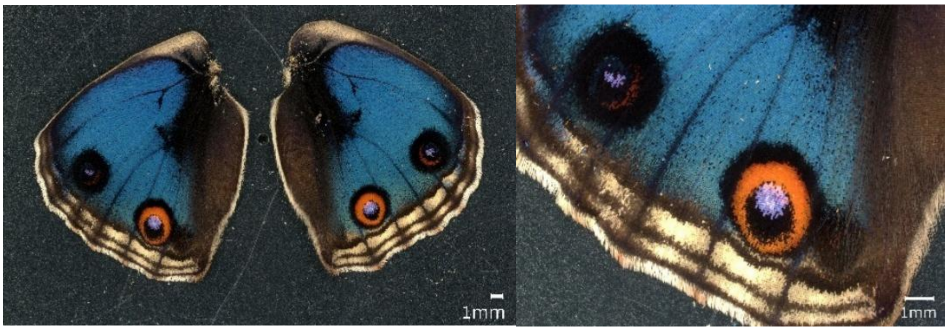

No. 11

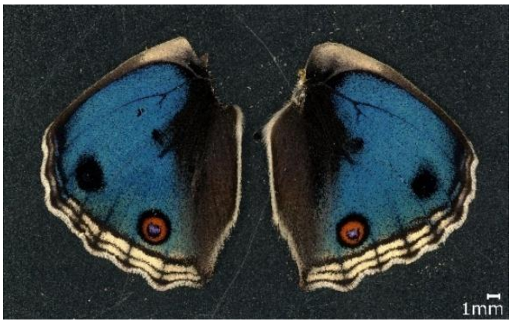

No. 12

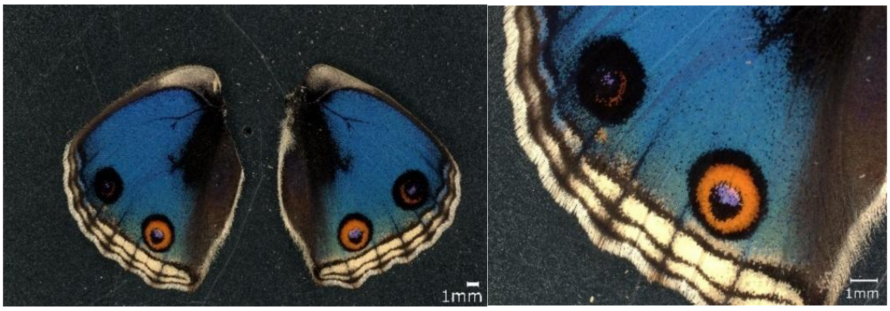

No. 13

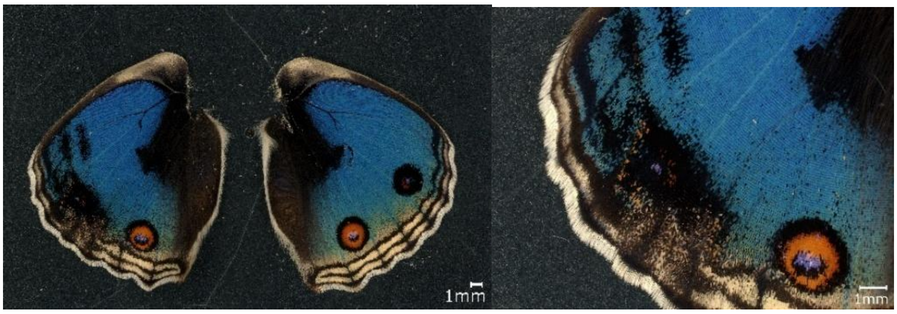

No. 14

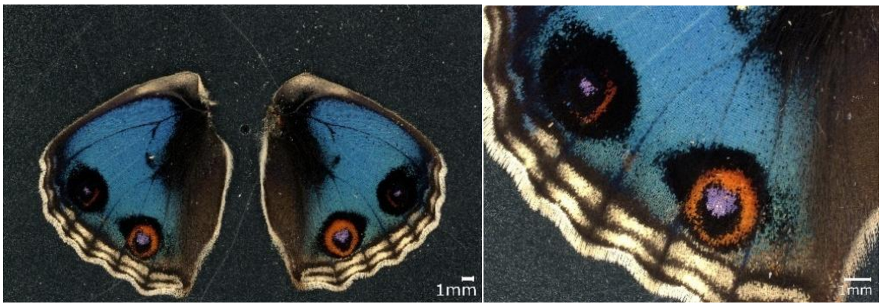

No. 15

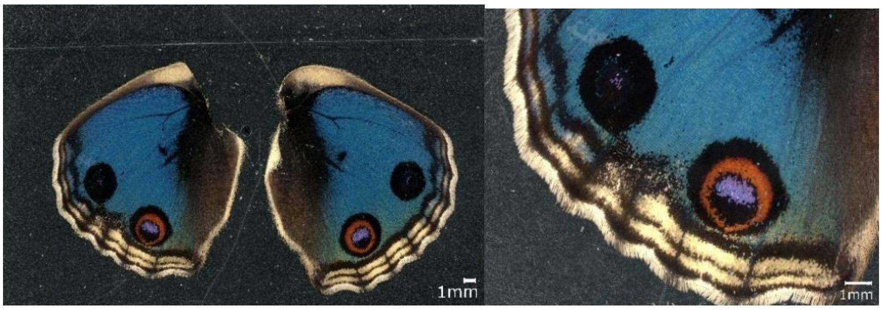

No. 16

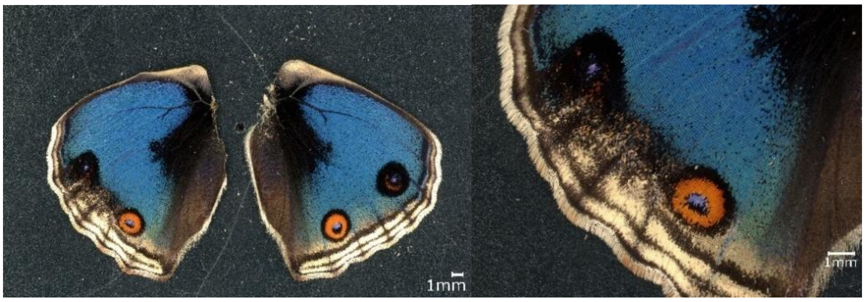

No. 17

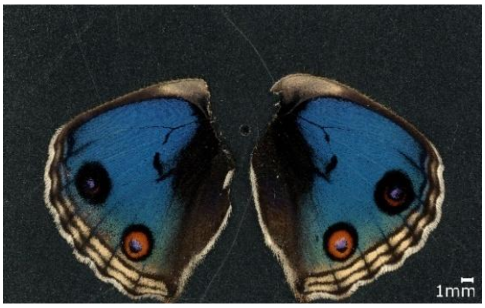

No. 18

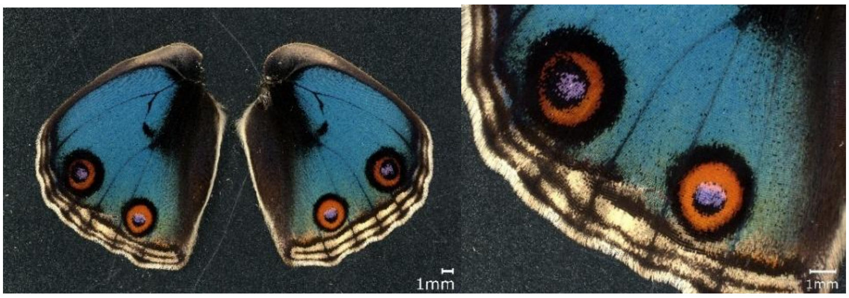

No. 19

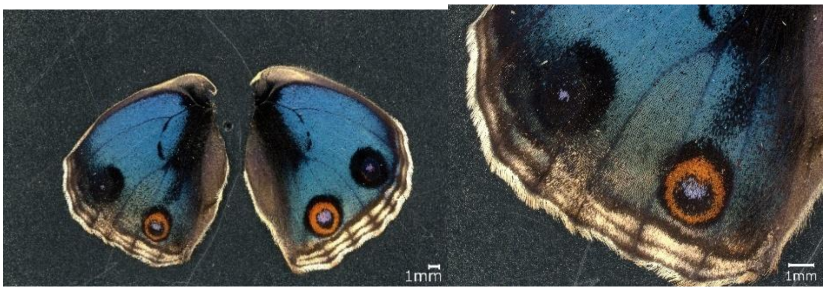

No. 20

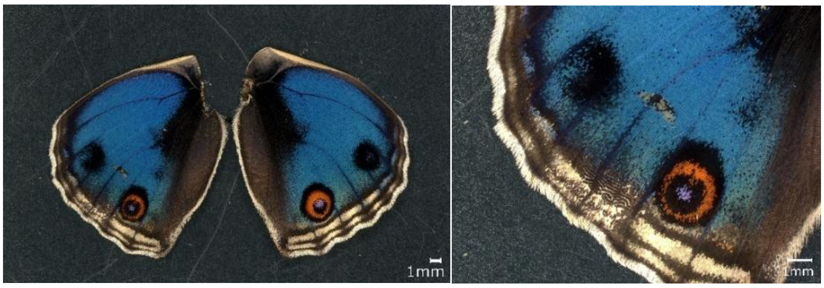

No. 21

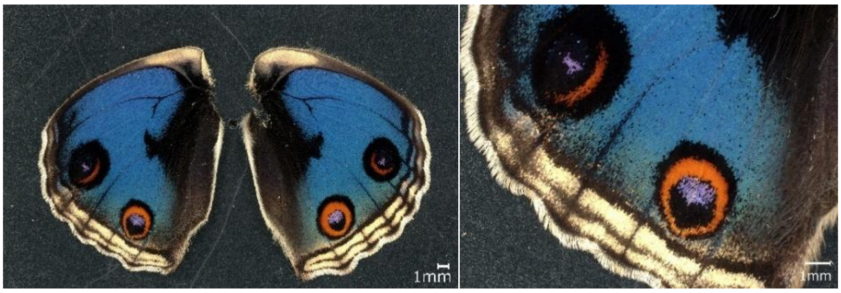

No. 22

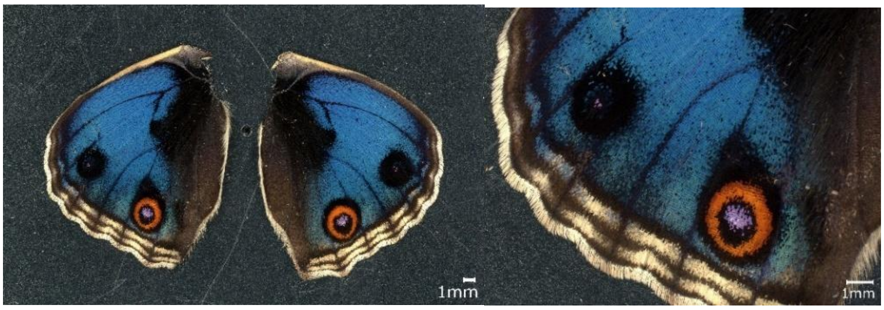

(**b**) Females (4.13 mM, *n* = 14).

No. 1

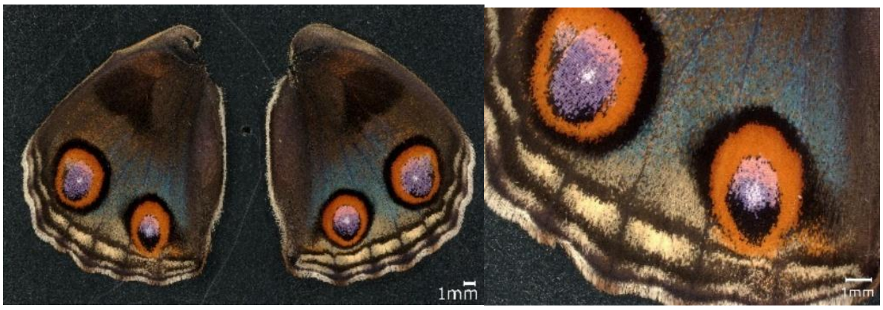

No. 2

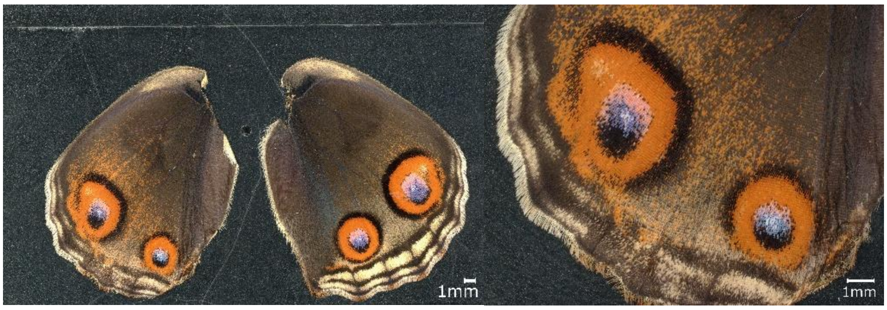

No. 3

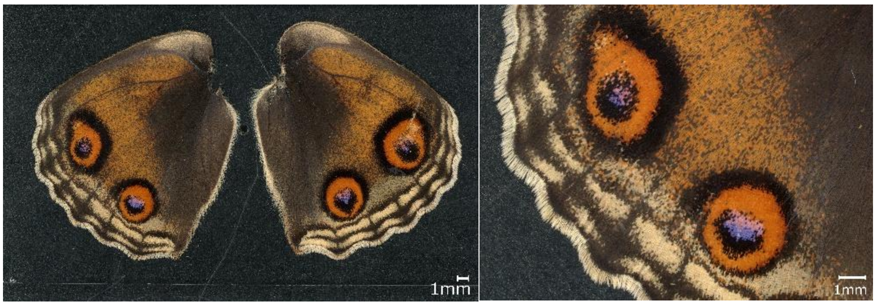

No.4

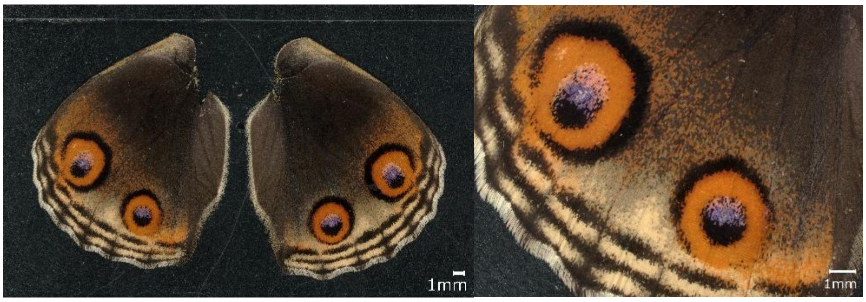

No.5

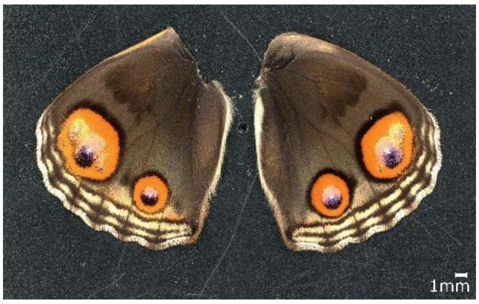

No. 6

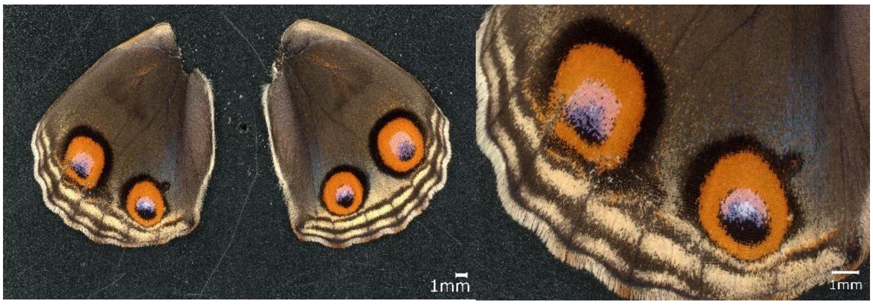

No. 7

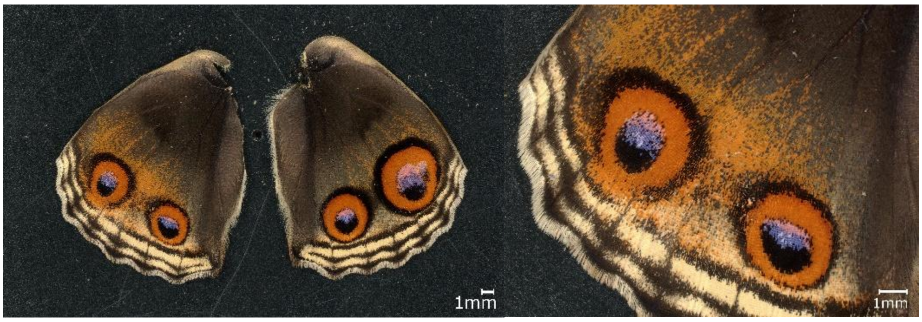

No.8

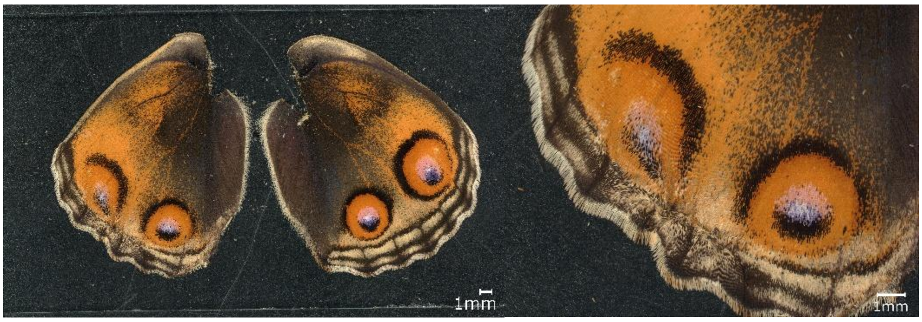

No. 9

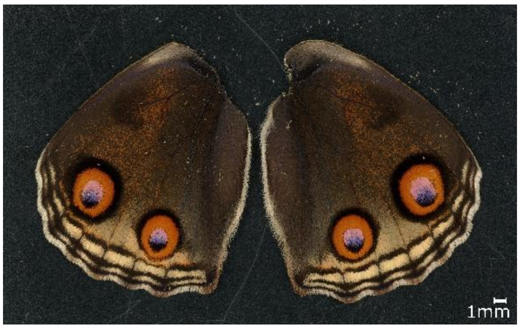

No. 10

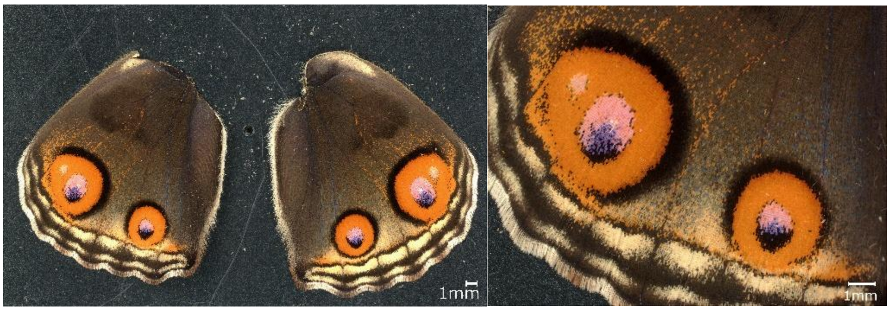

No. 11

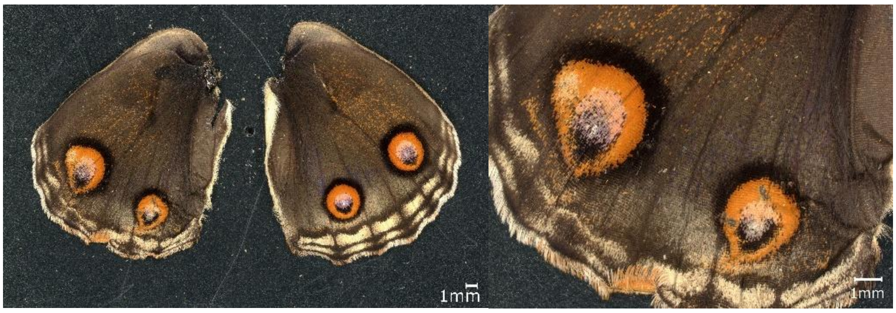

No. 12

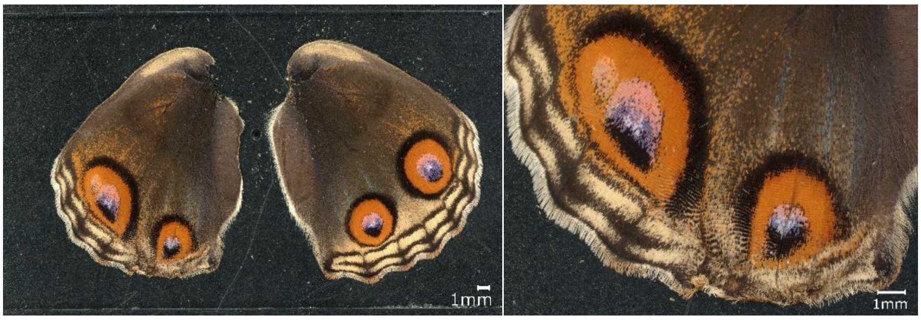

No. 13

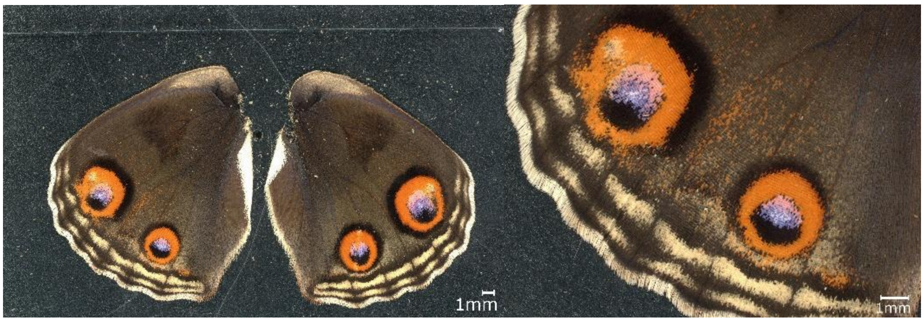

No. 14

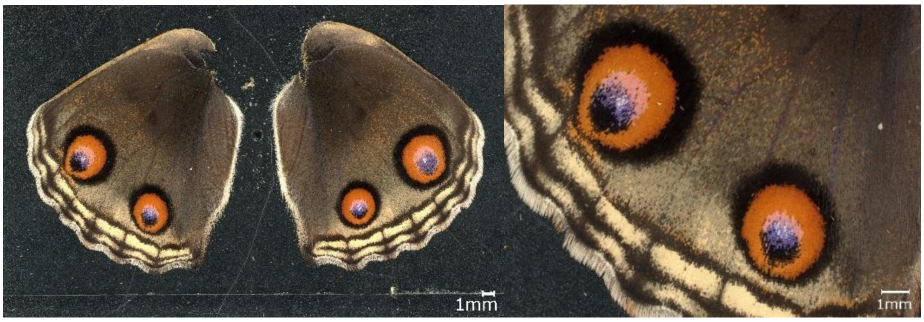

(**c**) Males (0.41 mM, *n* = 6).

No. 1

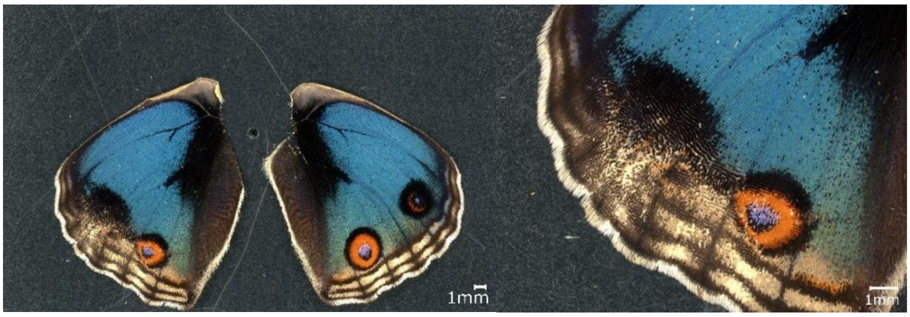

No.2

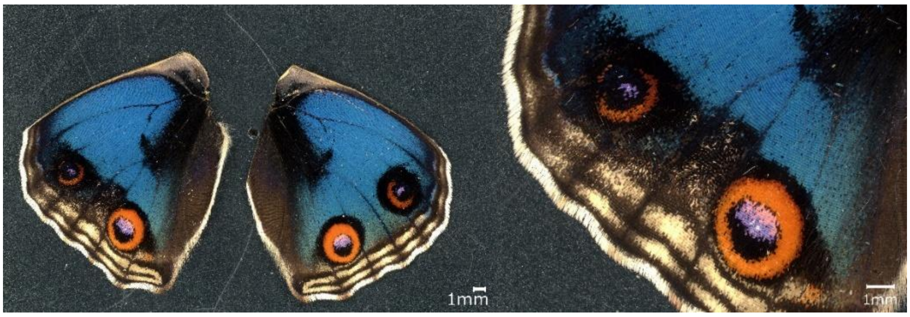

No. 3

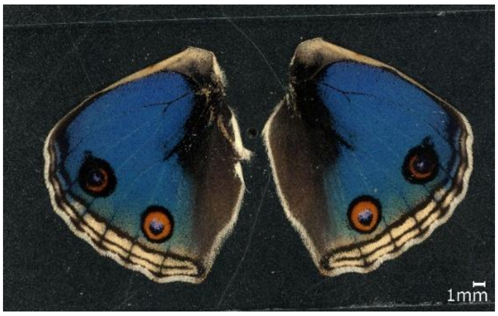

No. 4

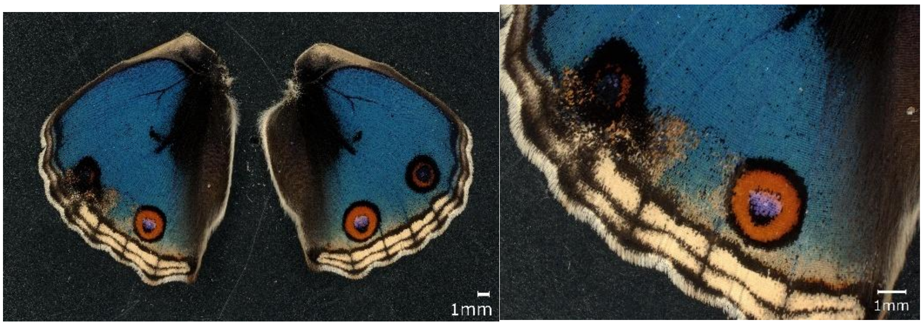

No. 5

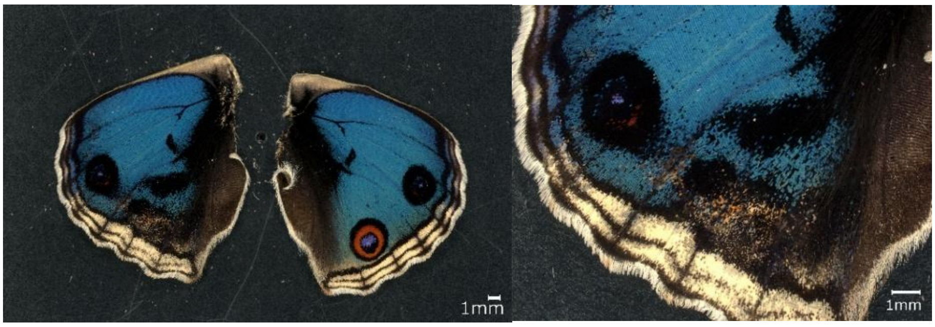

No. 6

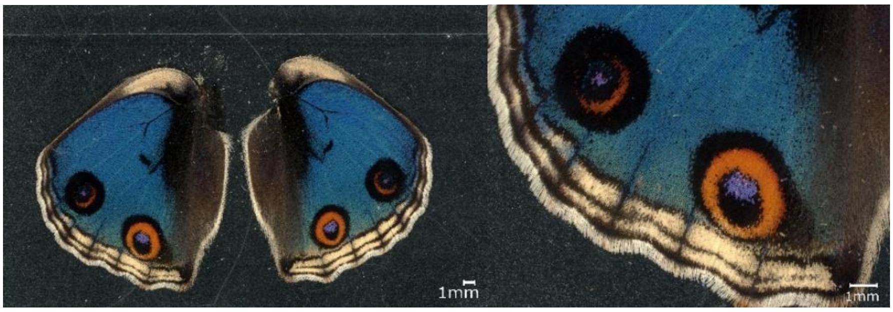

(**d**) Females (0.41 mM, n = 6).

No. 1

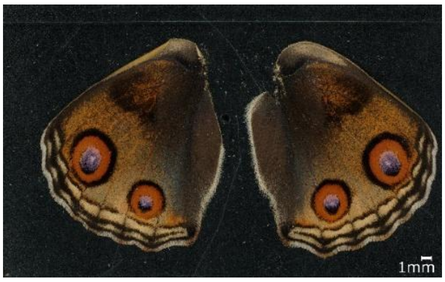

No.2

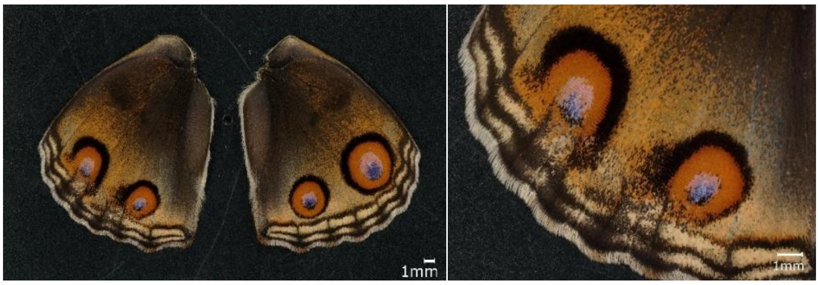

No. 3

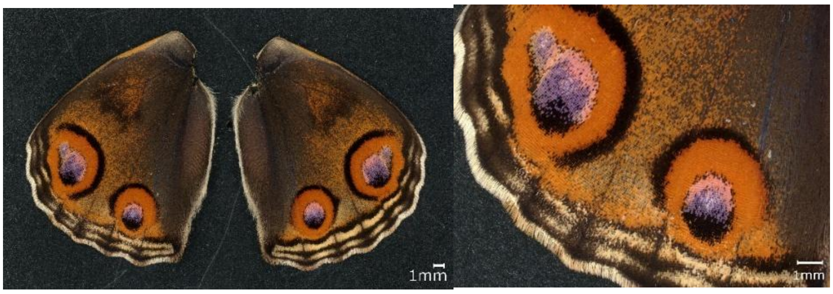

No. 4

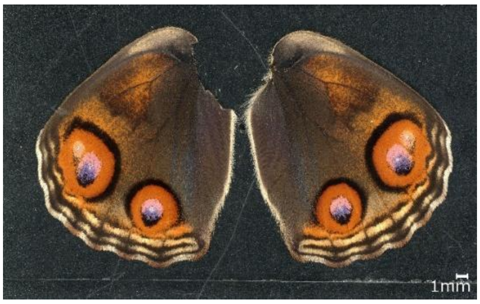

No. 5

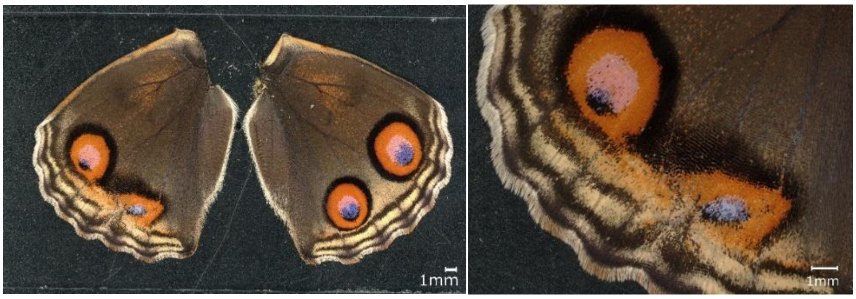

No. 6

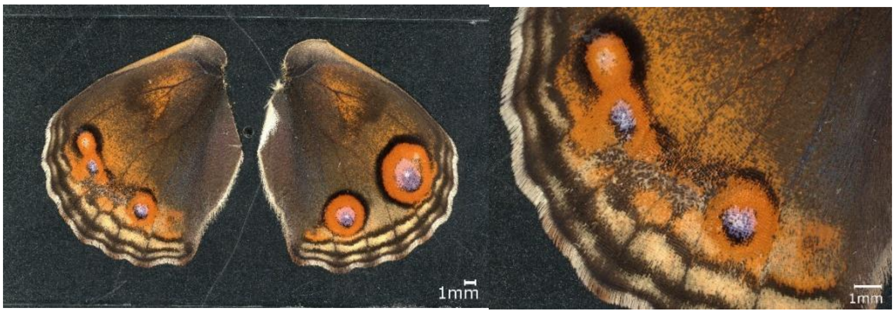

Supplement: Supplementary file 1 [file insects-17-00300-s001.zip › Supplementary Figure S5.pdf]
